# Prediction of perinatal death using machine learning models: a birth registry-based cohort study in northern Tanzania

**DOI:** 10.1136/bmjopen-2020-040132

**Published:** 2020-10-19

**Authors:** Innocent B Mboya, Michael J Mahande, Mohanad Mohammed, Joseph Obure, Henry G Mwambi

**Affiliations:** 1 School of Mathematics, Statistics, and Computer Science, University of KwaZulu-Natal, Pietermaritzburg, KwaZulu-Natal, South Africa; 2 Department of Epidemiology and Biostatistics, Institute of Public Health, Kilimanjaro Christian Medical University College, Moshi, Tanzania; 3 Department of Obstetrics and Gynecology, Kilimanjaro Christian Medical Center, Moshi, Tanzania

**Keywords:** reproductive medicine, neonatology, prenatal diagnosis, perinatology, epidemiology

## Abstract

**Objective:**

We aimed to determine the key predictors of perinatal deaths using machine learning models compared with the logistic regression model.

**Design:**

A secondary data analysis using the Kilimanjaro Christian Medical Centre (KCMC) Medical Birth Registry cohort from 2000 to 2015. We assessed the discriminative ability of models using the area under the receiver operating characteristics curve (AUC) and the net benefit using decision curve analysis.

**Setting:**

The KCMC is a zonal referral hospital located in Moshi Municipality, Kilimanjaro region, Northern Tanzania. The Medical Birth Registry is within the hospital grounds at the Reproductive and Child Health Centre.

**Participants:**

Singleton deliveries (n=42 319) with complete records from 2000 to 2015.

**Primary outcome measures:**

Perinatal death (composite of stillbirths and early neonatal deaths). These outcomes were only captured before mothers were discharged from the hospital.

**Results:**

The proportion of perinatal deaths was 3.7%. There were no statistically significant differences in the predictive performance of four machine learning models except for bagging, which had a significantly lower performance (AUC 0.76, 95% CI 0.74 to 0.79, p=0.006) compared with the logistic regression model (AUC 0.78, 95% CI 0.76 to 0.81). However, in the decision curve analysis, the machine learning models had a higher net benefit (ie, the correct classification of perinatal deaths considering a trade-off between false-negatives and false-positives)—over the logistic regression model across a range of threshold probability values.

**Conclusions:**

In this cohort, there was no significant difference in the prediction of perinatal deaths between machine learning and logistic regression models, except for bagging. The machine learning models had a higher net benefit, as its predictive ability of perinatal death was considerably superior over the logistic regression model. The machine learning models, as demonstrated by our study, can be used to improve the prediction of perinatal deaths and triage for women at risk.

**Strengths and limitations of this study**

- The Kilimanjaro Christian Medical Centre (KCMC) Medical Birth Registry cohort data provide a rich source of information for monitoring trends, inform clinical and administrative decisions, and enables complex modelling of the key predictors of perinatal deaths, among other adverse pregnancy outcomes.
- While standard regression models such as logistic regression are extensively applied in the literature to predict adverse pregnancy outcomes such as perinatal deaths, the application of machine learning models is limited.
- Machine learning algorithms may improve the prediction ability of perinatal deaths, and enable triage of women at high risk of experiencing adverse perinatal outcomes.
- The birth registry only captured deaths occurring in the KCMC hospital hence might have underestimated the proportion of perinatal deaths.

## Introduction

Neonatal survival is at the heart of Sustainable Development Goals agenda.^1 2^ The Every Newborn Action Plan to end Preventable Deaths set a goal for all countries to reach the target of ten or less newborn deaths per 1000 live births and 10 or less stillbirths per 1000 total births by the year 2035.^3^ Furthermore, the United Nations set the target of reducing neonatal mortality to 12 deaths per 1000 live births or fewer by 2030.^1^ Globally, neonatal deaths declined by 51% from 5 million in 1990 to 2.5 million in 2017. But this decline has not been realised in low-income and middle-income countries, which carries the highest burden of neonatal deaths, with south Asia and sub-Saharan Africa accounting for 79% of the total burden of neonatal deaths in 2017.^4^ Furthermore, the under-5 mortality rate has decreased almost across the world, but the proportions of neonatal deaths remained high in this group.^5 6^ Neonatal deaths accounted for 47% of all under-5 deaths in 2018, and it has increased from 40% in 1990, with sub-Saharan Africa bearing the highest burden.^6^ Globally 2.5 million children died in the first month of life in 2018, with approximately 7000 newborn deaths every day.^6^ Nearly three-quarters of these deaths occur during the first week, with about one million dying on the first day and close to one million dying within the next 6 days.^6^

Globally, more than five million perinatal deaths occur each year.^2^ The majority (95%) of these deaths occur in sub-Saharan Africa and Southern Asia.^7^ According to the Tanzania Demographic and Health Survey, the perinatal mortality rate has slightly increased from 36 to 39 deaths per 1000 live births between 2010–11 and 2015–16 survey rounds, respectively, relative to under-5 mortality.^8^ In addition, perinatal mortality rate in Tanzania is the highest in East Africa.^7^

Early identification of pregnant women at risk for adverse maternal and perinatal outcomes during the prenatal period and timely provision of high-quality healthcare services have been reported to improve maternal and newborn survival.^9^ Machine learning (hereafter denoted as ‘ML’) models are methodologies for developing algorithms that learn from existing data to make predictions on new data.^9^ ML models have shown better predictive performance over the classical or conventional regression models,^10^ and they can better handle a significant number of potential predictors. However, there is conflicting evidence of the performance of these models. Previous investigators have demonstrated that, compared with the classical regression models, ML models have superior performance for early differentiation of sepsis and non-infectious systemic inflammatory response syndrome in critically ill children,^11^ in predicting neonatal and under-5 mortality,^12–16^ and critical care and hospitalisation outcomes.^10 17 18^ In contrast, other studies have shown no predictive performance benefit of the ML models in prediction of clinical outcomes.^9 19^

The first step in addressing high perinatal mortality is the accurate capture and classification of the causes of those deaths across all settings.^20^ WHO International Classification of Diseases (ICD-10) is a standardised tool used for the classification of deaths occurring during the perinatal period: ICD-PM.^2 20 21^ ML models may be an essential tool in the assessment of risk factors for deaths during the perinatal period and triage pregnant women at high risk of experiencing adverse perinatal outcomes, especially in low-resourced settings where the majority of perinatal deaths occur at home.^22–25^ Capturing the chain of events that led to the perinatal mortality, from both the maternal and the perinatal side, informs the design and development of preventative and therapeutic measures.^2^

Using data from the medical birth registry at Kilimanjaro Christian Medical Centre (KCMC) referral hospital in northern Tanzania, we aimed to determine the key predictors of perinatal death using ML models. Previous studies using the same data^26–31^ applied standard regression models to assess risk factors for adverse perinatal outcomes. A major weakness of conventional regression analysis, as opposed to ML models, is that many covariates are excluded based on specific model assumptions. In contrast, ML techniques which are non-parametric in nature find the most predictive groupings of factors based on their frequency and strength of association, with no particular model assumptions.^32^ In this study, we compared the predictive performance of the ML models with the conventional regression analysis, particularly logistic regression (Lreg).

## Methods

### Study design, setting and population

We conducted a secondary analysis of birth cohort data from the KCMC referral hospital, situated in the Moshi Municipality of Kilimanjaro region, Northern Tanzania. The hospital receives deliveries from nearby communities and referral cases from other healthcare facilities inside the region and the neighbouring regions.^33^ The hospital has an average annual delivery rate of 4000 births.^31 33 34^ The study population was women who delivered singleton babies. We included 42 319 deliveries with complete records between 2000 and 2015. We excluded records with missing values on the outcome (perinatal status) and the covariates as well as pregnancies with multiple gestations to avoid over-representation of high-risk pregnancies.^31^

### Data source

We used data from the KCMC referral hospital medical birth registry between the years 2000 and 2015, which were collected among mothers who delivered at the department of obstetrics and gynaecology. More description of the KCMC medical birth registry is also available elsewhere.^26 28 31 35 36^ Briefly, the KCMC medical birth registry is within hospital grounds at the Reproductive and Child Health Centre. The birth registry has been in operation since the year 2000, established to serve both clinical, administrative and research purposes.^35 36^ Trained midwives collected data using a standardised questionnaire (within 24 hours after delivery or later in case a mother had recovered from complications), after which data are entered into a computerised database located at the birth registry. Also, additional data were abstracted from the antenatal care (ANC) cards and the hospital medical records of the mother.^28^

A unique hospital identification number was assigned to each woman at first admission and used to trace her medical records at later admissions, and further to link records of successive births of the same woman.^36^ Data captured information on the background characteristics of mother and father, mother’s health before and during present pregnancy, information about delivery including complications, and child characteristics including their status (ie, whether dead or alive).

### Study variables

The main outcome variable in this study was perinatal death which was defined as the number of stillbirths (pregnancy loss that occurs after 7 months of gestation) and early neonatal deaths (deaths of live births within the first 7 days of life).^8 37^ The perinatal death was coded as binary, that is, ‘yes’ if death occurred during the perinatal period and ‘no’ if otherwise. This outcome only captured deaths that occurred within the hospital before the discharge of mothers. There are no follow-up mechanisms for deaths that occur outside the health facility (KCMC hospital).

We included a total of 32 predictor variables for the ML models. Previous literature informed the selection of these variables,^4 27 38–45^ most of which are available in the birth registry. These included maternal and paternal background characteristics; age in years, area of residence (rural vs urban), highest education level (none, primary, secondary and higher), marital status (single, married and widow/divorced) and occupation (unemployed, employed and others). Further, specific characteristics of the mother included referral status (whether referred for delivery or not), and the number of ANC visits (<4 and ≥4 visits).

We excluded maternal body mass index (BMI) and HIV status because they contributed to nearly 47% of all missing values in the dataset. Maternal health during pregnancy included; alcohol consumption, smoking, gestational diabetes, diabetes, hypertension, pre-eclampsia/eclampsia, bleeding (ie, the woman observed blood from the vagina at any time during the pregnancy), anaemia, malaria and systemic infections/sepsis. Variables with information concerning delivery included; induction of labour (yes or no), mode of delivery (vaginal vs caesarean section), presentation (breech vs cephalic), complications during birth, particularly premature rupture of the membranes, postpartum haemorrhage, placenta previa and placenta abruption, all categorised as yes and no. Gestational age at birth was estimated based on the date of the last menstrual period and recorded in full weeks. Preterm birth was defined as babies born alive before 37 weeks of pregnancy are completed.^46^ Child characteristics included sex (male or female), low birthweight defined as an infant birth weight of less than 2500 g^27 47^ and year of birth.

### Statistical and computational analysis

Data were cleaned and then analysed using Stata V.15.1.^48^ Categorical variables were summarised using frequencies and proportions. The χ^2^ test statistic was used to test the relationships between a set of independent variables and perinatal death. For the ML models (ie, from feature selection, training, testing and comparison of the predictive performance of the machines), we used R V.3.6.3.^49^ The training dataset contained 70% of randomly selected samples used to develop six different ML models to predict perinatal death. These are artificial neural networks (ANN), random forests (RF), Naïve Bayes (NB), bagged trees, boosting and the Lreg model. We used the *caret* package to implement these models in R.

Briefly, ANN is a method constructed from three layers of connected nodes: input, hidden and output.^50^ The input where each input variable appears as a node; the hidden layer contains several nodes determined during the model tuning phase. In contrast, the output layer contains several nodes equal to the number of classes to be predicted.^51^ Between these layers, there are weighted links,^9 50 51^ the hidden layer receives a sum of the multiplication of the input variables with associated weights values plus the bias.^50 51^ This value is entered into an activation function, such as a logistic or sigmoid function, to decide the class prediction. Outputs of the network are interpreted as class probabilities and sum to one.^51^ We used nnet package to construct the ANN model.

RF is an extension of classification and regression trees.^9 10 51 52^ RF performance is better compared with bagged trees because it decorrelates the trees,^53^ hence improves accuracy.^52^ Several forests of decision trees are grown using a random bootstrapped training sample. Also, instead of using all the variables/features in each tree, a random sample of variables are selected and tested at each split in each tree.^10 51 52^ The prediction is made for unobserved data by taking a majority vote of the individual trees.^51 52^ We used *randomForest* package to construct the RF model. NB is an effective classifier^50^ due to its simplicity, exhibiting a surprisingly competitive predictive accuracy.^54^ NB uses probability theory to find the most possible sample class in a classification problem. NB has two assumptions: (1) each attribute is conditionally independent of the other attributes given the class and (2) all the attributes have an impact on the class.^51 54^ We used naivebayes package to construct the NB model.

Lreg is a standard multivariate classification method. It arises from the desire to model the posterior probabilities via linear functions in covariates, such that besides predicting class labels, it provides a probabilistic interpretation of this labeling.^53 55 56^ Lreg uses a sigmoid function instead of a linear function to map predictions to probabilities between 0 and 1.^53^ We used glm method to construct the Lreg model. Bagging, or bootstrap aggregation and boosting are general techniques for improving prediction rules and accuracy of the resulting predictions, by reducing the associated variance of prediction.^53 57^ Bagging divides the available data into many bootstrap samples and then train a separate model for each bootstrap, and then make a final prediction by averaging and voting for regression and classification, respectively.^57^ Boosting, on the other hand, is a committee-based approach that uses a weighted average of prediction from various samples. The incorrectly predicted cases from a given step are given a higher weight during the next step. Thus, it is an iterative procedure, incorporating weights, as opposed to simple averaging of predictions.^57^ We used *treebag* method and gbm package to construct the bagging and boosting models, respectively.

In the training set, parameter tuning and cross-validation aim to find a balance between building a model that can classify the training data effectively without overfitting to the random fluctuations.^51^ For each ML model, we used 10-fold cross-validation as a resampling method, where the training set is divided equally into 10 parts (folds). Therefore, every nine folds are used together for training the model and the remaining onefold for testing. This training-testing process is repeated ten times. We performed feature selection using the RF algorithm. After selecting the most important features, we retained in the dataset and used them for analysis in both the training and testing data for all models. We used the Synthetic Minority Over-sampling Technique (SMOTE) method^58 59^ to address the class imbalance in the outcome (ie, the low proportion of perinatal deaths), by specifying the additional sampling to be ‘smote’ on train control parameter specifications. SMOTE is a method that produces artificial minority samples by interpolating between existing minority samples and their nearest minority neighbors.^58 59^

Using the testing set (30% of the remaining randomly selected sample), we computed the predictive performance of the six models (including Lreg model) from the training set using the area under the receiver-operating-characteristics curve (AUC ROC). We used the *ROCR* package for plotting ROC curves, obtaining the AUC values and comparison of models using AUC values. We also used measures from the confusion matrix results (ie, accuracy, sensitivity, specificity, positive and negative predictive values (NPV)), and the net benefit through decision curve analysis^60 61^—which quantifies whether a machine provides a relevant improvement in the prediction. We used epiR package to obtain confidence intervals for the performance measures and DCA package (http://www.decisioncurveanalysis.org) for decision curve analysis. We further used ggplot2 package to plot the decision curves. A good model will have a higher net benefit.^60^ We used Delong’s test to compare the ROC between models, where, a p<0.05 was considered statistically significant. The variable importance is a scaled measure with a maximum value of 100.^17^ The R code used for this analysis is shown in online supplemental material 1.

10.1136/bmjopen-2020-040132.supp1 Supplementary data

### Patient and public involvement

There was no patient and public involvement.

## Results

### Characteristics of study participants

**Figure 1:**
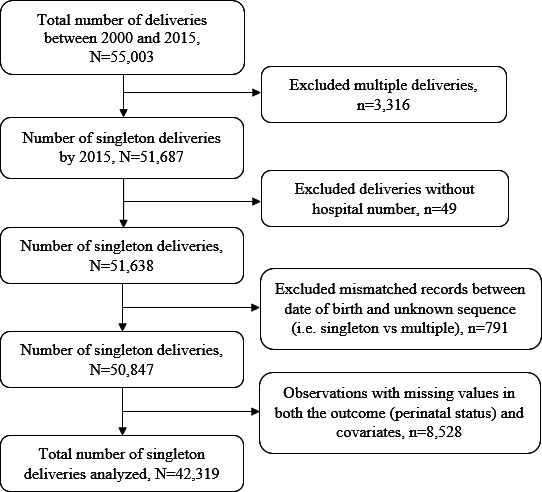
Schematic diagram showing the number of singleton deliveries analysed, KCMC medical birth registry data, 2000–2015. KCMC, Kilimanjaro Christian Medical Centre.

The characteristics of the participants are shown in table 1. A total of 55 003 total deliveries were recorded at the KCMC medical birth registry from 2000 to 2015. Of these, we excluded 3316 (6%) multiple gestations (to avoid over-representation of high-risk pregnancies),^31^ 49 (0.1%) records missing maternal identification numbers (hence could not be linked to child records), 791 (1.4%) records with a mismatch between the date of birth and unknown sequence (ie, singleton vs multiple births). We further excluded a total of 8528 (15.5%) observations with missing values in both the outcome (perinatal status) and covariates. We, therefore, analysed data for a total of 42 319 singleton deliveries with complete records (figure 1).

**Table 1:** Characteristics of study participants (n=42 319)

Characteristics	Total	Perinatal death	P value*	Characteristics	Total	Perinatal death	P value*
Maternal	n (%)	n (%)		Obstetrics	n (%)	n (%)	
**Age (years**)			<0.001	**Gestational diabetes**			0.89
15–19	3470 (8.2)	99 (2.9)		No	42 288 (99.9)	1560 (3.7)	
20–34	32 675 (77.2)	1158 (3.5)		Yes	31 (0.1)	1 (3.2)	
35–39	4984 (11.8)	235 (4.7)		**Diabetes**			0.002
40+	1190 (2.8)	69 (5.8)		No	42 240 (99.8)	1553 (3.7)	
**Education level**			<0.001	Yes	79 (0.2)	8 (10.1)	
None	567 (1.3)	32 (5.6)		**Hypertension**			<0.001
Primary	23 010 (54.4)	1019 (4.4)		No	42 241 (99.8)	1550 (3.7)	
Secondary	5275 (12.5)	159 (3.0)		Yes	78 (0.2)	11 (14.1)	
Higher	13 467 (31.8)	351 (2.6)		**Bleeding**			<0.001
**Occupation**			0.37	No	41 897 (99.0)	1528 (3.6)	
Unemployed	9316 (22.0)	365 (3.9)		Yes	422 (1.0)	33 (7.8)	
Employed	30 061 (71.0)	1085 (3.6)		**Anaemia**			0.004
Others	2942 (7.0)	111 (3.8)		No	41 661 (98.4)	1523 (3.7)	
**Marital status**			0.89	Yes	658 (1.6)	38 (5.8)	
Single	4954 (11.7)	186 (3.8)		**Malaria**			0.79
Married	37 300 (88.1)	1372 (3.7)		No	36 746 (86.8)	1352 (3.7)	
Widowed/divorced	65 (0.2)	3 (4.6)		Yes	5573 (13.2)	209 (3.8)	
**Area of residence**			<0.001	**Sepsis/infections**			0.43
Urban	25 056 (59.2)	725 (2.9)		No	41 588 (98.3)	1538 (3.7)	
Rural	17 263 (40.8)	836 (4.8)		Yes	731 (1.7)	23 (3.1)	
**Alcohol consumption during pregnancy**			0.001	**Complications**			
No	30 759 (72.7)	1191 (3.9)		**Pre-eclampsia/ eclampsia**			<0.001
Yes	11 560 (27.3)	370 (3.2)		No	40 668 (96.1)	1355 (3.3)	
**Smoking during pregnancy**			0.97	Yes	1651 (3.9)	206 (12.5)	
Yes	53 (0.1)	2 (3.8)		**Induction of labour**			<0.001
No	42 266 (99.9)	1559 (3.7)		No	32 732 (77.3)	1105 (3.4)	
**No of ANC visits**			<0.001	Yes	9587 (22.7)	456 (4.8)	
≥4	28 742 (67.9)	760 (2.6)		**PROM**			0.006
<4	13 577 (32.1)	801 (5.9)		No	41 416 (97.9)	1543 (3.7)	
**Referred for delivery**			<0.001	Yes	903 (2.1)	18 (2.0)	
No	32 762 (77.4)	819 (2.5)		**PPH**			<0.001
Yes	9557 (22.6)	742 (7.8)		No	42 091 (99.5)	1516 (3.6)	
**Paternal characteristics**				Yes	228 (0.5)	45 (19.7)	
**Age (years**)			0.001	**3–4 degree tear**			0.49
<25	3938 (9.3)	122 (3.1)		No	42 305 (99.9)	1560 (3.7)	
25–29	10 593 (25.0)	346 (3.3)		Yes	14 (0.1)	1 (7.1)	
30–34	12 303 (29.1)	457 (3.7)		**Abruption placenta**			<0.001
35+	15 485 (36.6)	636 (4.1)		No	42 193 (99.7)	1490 (3.5)	
**Education level**			<0.001	Yes	126 (0.3)	71 (56.3)	
None	281 (0.7)	27 (9.6)		**Placenta previa**			0.04
Primary	18 987 (44.9)	868 (4.6)		No	42 245 (99.8)	1555 (3.7)	
Secondary	4565 (10.8)	154 (3.4)		Yes	74 (0.2)	6 (8.1)	
Higher	18 486 (43.7)	512 (2.8)		**Presentation**			<0.001
**Occupation**			<0.001	Cephalic	41 833 (98.9)	1459 (3.5)	
Unemployed	5710 (13.5)	323 (5.7)		Breach/ Transverse	486 (1.1)	102 (21.0)	
Employed	36 102 (85.3)	1218 (3.4)		**Gestational age at birth**			<0.001
Others	507 (1.2)	20 (3.9)		Term birth (≥37 weeks)	37 764 (89.2)	914 (2.4)	
				Preterm birth (<37 weeks)	4555 (10.8)	647 (14.2)	
				**Birth weight**			<0.001
				Normal birth weight	37 991 (89.8)	801 (2.1)	
				Low birth weight	4328 (10.2)	760 (17.6)	
				**Child’s sex**			0.42
				Female	20 430 (48.3)	738 (3.6)	
**Total**	42 319	1561 (3.7%)		Male	21 889 (51.7)	823 (3.8)	

*P value based on the χ^2^ test.

ANC, antenatal care; PPH, postpartum haemorrhage; PROM, premature rupture of the membranes.

The overall proportion of perinatal death among 42 319 singleton deliveries in this study was 3.7%. The proportion of perinatal deaths among mothers aged 20–34, 35–39 and 40+ years was 3.5%, 4.7% and 5.8%, respectively. Mothers with no education (5.6%) and those with primary education level (4.4%), who resided in rural areas (4.8%), had less than four ANC visits (5.9%), and those referred for delivery (7.8%) had a higher proportion of perinatal death. Among fathers, a higher proportion of perinatal death is among those aged 35+years (4.1%), with no (9.6%) or with primary education level (4.6%) as well as those who were unemployed (5.7%), table 1.

Furthermore, the most common obstetric care and complications in this birth cohort included induction of labour (22.7%), malaria (13.2%), preterm birth (10.8%) and LBW (10.2%). About 4% of mothers in this cohort experienced pre-eclampsia/eclampsia during pregnancy. Less than half of all children were females. The proportion of perinatal death among women who experienced induction of labour, with malaria, delivered preterm, delivered LBW baby and experienced pre-eclampsia/eclampsia during pregnancy was 4.8%, 3.8%, 14.2%, 17.6% and 12.5%, respectively. The proportion of perinatal death is almost similar among males (3.8%) compared with females (3.6%) children in this cohort (table 1).

**Figure 2:**
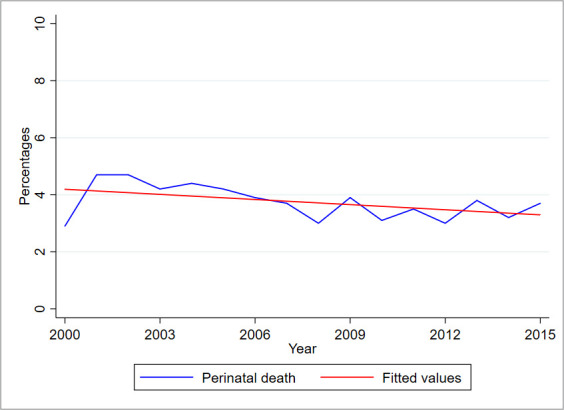
Trends of perinatal death, KCMC medical birth registry data, 2000–2015. KCMC, Kilimanjaro Christian Medical Centre.

The trends in the proportion of perinatal deaths that occurred at KCMC between the years 2000–2015 are shown in figure 2. Overall, the proportion of perinatal deaths has slightly declined over the years by 6% (95% CI, 0.3% to 12.3%), though this decline was not statistically significant (p=0.06).

### Variable importance

**Figure 3:**
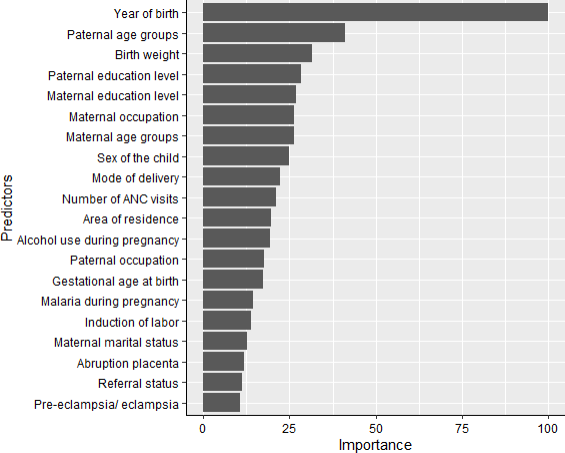
Variable importance of predictors for perinatal death in the random forest model scaled to have a maximum value of 100. ANC, antenatal care.

We used the RF algorithm for feature/variable selection. This model selected a total of 20 important predictors (figure 3) based on its threshold measure of importance out of the 32 variables. We used these 20 variables in all the subsequent analysis for all models in both training and testing sets.

### Predicting perinatal deaths

**Figure 4:**
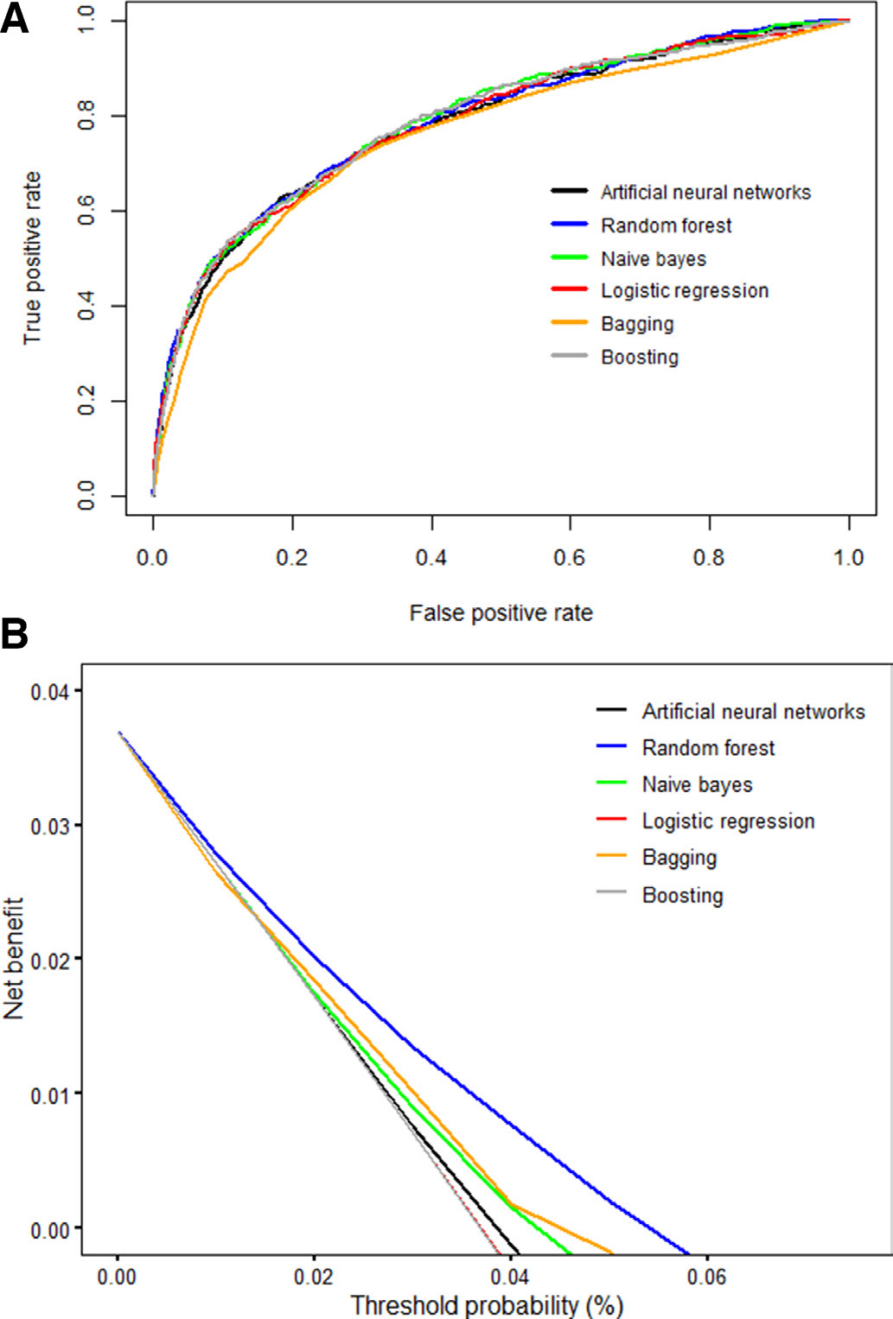
Prediction ability of perinatal deaths comparing different machine learning models in the test set: (A) Receiver operating characteristics curves. The corresponding values of the area under the receiver operating characteristics curve for each model are in table 2. (B) Decision curve analysis. The net benefit of the machine learning models (except for boosting) is larger over a range of threshold probability values compared with that of the logistic regression model.

The discriminatory abilities of all models for the prediction of perinatal death are in figure 4A and table 2. There were no significant differences (p>0.05) in the AUC the ROC curve between Lreg with RF, ANN, boosting and NB. However, bagging had significantly lower predictive performance (AUC 0.76, 95% CI 0.74 to 0.79, p=0.006) compared with the Lreg model (AUC 0.78, 95% CI 0.76 to 0.81). Furthermore, the ANN model (sensitivity 0.60, 95% CI 0.55 to 0.64) and NB model (Sensitivity 0.57, 95% CI 0.52 to 0.62) had slightly higher sensitivity compared with Lreg (sensitivity 0.56, 95% CI 0.51 to 0.60) while boosting (Specificity 0.89 95% CI 0.88 to 0.89) and RF (Specificity 0.88, 95% CI 0.88 to 0.89) had slightly higher specificity compared with Lreg (specificity 0.87, 95% CI 0.86 to 0.88). Due to the low prevalence of perinatal deaths (3.7%), all models had high (NPV 0.98, 95% CI 0.98 to 0.98).

**Table 2:** Prediction performance of the reference and machine learning models in the test set

Model	Logistic regression	Artificial neural network	Random forests	Naïve bayes	Bagging	Boosting
ACC	0.86 (0.85 to 0.86)	0.83 (0.82 to 0.83)	0.87 (0.86 to 0.87)	0.84 (0.83 to 0.85)	0.82 (0.81 to 0.83)	0.87 (0.87 to 0.88)
AUC	0.78 (0.76 to 0.81)	0.78 (0.76 to 0.80)	0.79 (0.76 to 0.81)	0.79 (0.76 to 0.81)	0.76 (0.74 to 0.79)	0.79 (0.76 to 0.81)
P value*	Reference	0.59	0.37	0.65	0.006	0.20
Sensitivity	0.56 (0.51 to 0.60)	0.60 (0.55 to 0.64)	0.54 (0.49 to 0.58)	0.57 (0.52 to 0.62)	0.55 (0.50 to 0.59)	0.54 (0.49 to 0.58)
Specificity	0.87 (0.86 to 0.88)	0.84 (0.83 to 0.84)	0.88 (0.88 to 0.89)	0.85 (0.84 to 0.86)	0.83 (0.82 to 0.84)	0.89 (0.88 to 0.89)
PPV	0.14 (0.12 to 0.16)	0.12 (0.11 to 0.14)	0.15 (0.13 to 0.17)	0.13 (0.11 to 0.14)	0.11 (0.10 to 0.12)	0.15 (0.14 to 0.17)
NPV	0.98 (0.98 to 0.98)	0.98 (0.98 to 0.98)	0.98 (0.98 to 0.98)	0.98 (0.98 to 0.98)	0.98 (0.98 to 0.98)	0.98 (0.98 to 0.98)

*We calculated p values to compare the AUC the receiver operating characteristics curve of logistic with each machine learning model.

ACC, accuracy; AUC, area under the curve; NPV, negative predictive value; PPV, positive predictive value.

With regard to the number of actual and predicted outcomes (table 3), all models correctly predicted perinatal deaths by more than half of 468 deaths in the testing set. The numbers of correct classification were higher in the ANN 280 (59.8%) and NB 267 (57.1%), followed by the Lreg model 261 (55.8%) and bagging 260 (55.6%). The decision curve analysis (figure 4B) demonstrated that the net benefit of the RF model surpassed that of other ML models, including Lreg for all threshold values, indicating that the RF model is more superior in predicting the risk of perinatal deaths in this cohort. The accuracy of the RF model was 0.87, 95% CI (0.86 to 0.87), compared with 0.87, 95% CI (0.87 to 0.88) for boosting and 0.86, 95% CI (0.85 to 0.86) for the Lreg model (table 2). Furthermore, other ML models (except for boosting) demonstrated high net benefit over a range of threshold probability values relative to that of the Lreg model. Also, the RF model had a superior net benefit over all models (figure 4B).

**Table 3:** The number of actual and predicted outcomes of prediction models in the test set

Prediction model	Classification	Perinatal status	
		Alive	Died
	Actual number of events	12 227	468
Logistic regression	Correctly predicted outcome	10 627	261
	Incorrectly predicted outcome	1600	207
Artificial neural network	Correctly predicted outcome	10 225	280
	Incorrectly predicted outcome	2002	188
Random Fforests	Correctly predicted outcome	10 774	251
	Incorrectly predicted outcome	1453	217
Naïve bayes	Correctly predicted outcome	10 386	267
	Incorrectly predicted outcome	1841	201
Bagging	Correctly predicted outcome	10 175	260
	Incorrectly predicted outcome	2052	208
Boosting	Correctly predicted outcome	10 852	252
	Incorrectly predicted outcome	1375	216

## Discussion

In this study, the perinatal death was predicted using five ML models (ANN, RFs, NB, bagging and boosting). There were no differences in the predictive performance between ML models except for bagging, which had a lower predictive performance. The ANN and NB had higher sensitivity compared with the Lreg, and other ML models. Specificity for all models was high, mainly due to the low prevalence of perinatal deaths in this cohort. Additionally, results from the decision curve analysis revealed that the ML models (except for boosting) had a higher net benefit over a range of threshold probability values compared with the Lreg model, indicating high accuracy. The RF model demonstrated a superior net benefit over other models.

In the present study, maternal characteristics before and during pregnancy, pregnancy history, and paternal characteristics identified pregnancies at high risk of experiencing adverse perinatal outcomes that might need close clinical follow-ups. It is worth noting here that paternal age and education level were highly predictive of perinatal death more than the known pregnancy-related conditions or complications such as prematurity. Previous literature shows that paternal characteristics, particularly advanced paternal age, increase the risk of adverse perinatal outcomes, such as low birth weight, prematurity, small for gestational age and low Apgar scores,^62–64^ despite conflicting evidence from other studies.^65^ Furthermore, studies using data from the KCMC Medical Birth Registry (same data source to the current study) focused on modelling the association between maternal and pregnancy-related characteristics and complications during pregnancy and childbirth with the risk of adverse perinatal outcomes^26–31^ but ignored paternal characteristics. Despite challenges in male involvement in pregnancy and childbirth in Tanzania,^66 67^ their participation is critical to improving maternal and child health outcomes.

On top of clinicians’ judgement, previous investigators applied standard regression models in prediction of risk for adverse perinatal outcomes, particularly perinatal death.^29 39–45 68–73^ We found no differences in the predictive performance of the ML models, except for bagging, which had lower predictive capacity. The sensitivity of the ML models was also almost comparable to that of Lreg, which indicates that both models correctly classified perinatal deaths. Our finding is consistent with a recent systematic review that showed no performance benefit of ML models over Lreg for the prediction of clinical outcomes.^19^ The possible explanation for lack of differences in the performance between the compared models could be attributed to the low proportion of outcome and exposures in this study, as well as data quality and recording challenges inherent in registry-based studies.

In contrast, some previous investigators have demonstrated that ML models offer better predictions of clinical or adverse pregnancy outcomes compared with classical regression models.^13–16^ The application of ML models may improve the classification of adverse events occurring during the perinatal period and, therefore, assist in triaging and provision of close clinical follow-up for women at high risk. Other studies also provide evidence of improved prediction of under-5 and neonatal mortality^12–15^ using ML models. The utility of these models may, therefore, improve the prediction of adverse pregnancy outcomes as opposed to standard regression models.

In this study, the decision curve analysis that accounts for the impact of false-negative and false-positive misclassification errors showed superior predictive performance of the ML approaches over the Lreg model. This demonstrates a higher net benefit for the prediction of perinatal deaths. The higher net benefit in the prediction ability of the ML approaches has also been documented elsewhere.^10 17^ This is because ML approaches can incorporate the high order nonlinear interactions between predictors, which cannot be addressed by traditional modelling approaches, including the Lreg model. Furthermore, the use of cross-validation is also known to reduce potential overfitting in ML models. It is important to note that ML approaches are, to a large extent, non-parametric as opposed to the Lreg model that relies on strong distributional assumptions.

The strength of this study is that it is the first to apply modern ML approaches to predict perinatal deaths, particularly in Tanzania and to a large extent sub-Saharan Africa, compared with the classical Lreg model. Our study demonstrated that ML models might be used to improve the prediction of perinatal deaths and triage of women at risk. We also used the SMOTE balancing technique to avoid the bias of the model toward skewed data (reduce overfitting), hence improving the prediction accuracy of the ML algorithm.^14 58 59^ However, SMOTE is not very effective for high dimensional data.^74 75^ Our study also had some limitations that are worth considering when interpreting the results. First, we excluded observations with missing values in both the outcome and exposures from the analysis, a problem inherent in cohort studies, including birth registries, which may lead to under-estimation of the proportion of perinatal death. Two excluded variables (maternal BMI and HIV status) have been associated with perinatal and under-5 deaths^4 6 38^; hence their exclusion might increase the risk of residual confounding bias. The effect of exclusion of these two variables and missing values to predict perinatal deaths remains unquantified.

Second, selection bias/or referral bias is a common problem in hospital-based studies, which affects the generalisation of findings to the general population. This might also be the case in the present study. However, our findings might reflect a similar setting in Tanzania and probably in other sub-Saharan African countries. Third, the KCMC Medical Birth Registry cohort only captures perinatal deaths occurring in the health facility (KCMC hospital), which may underestimate the observed perinatal deaths in the wider population. Currently, the hospital has no mechanisms to follow-up the birth outcomes from deliveries that occur at home and postdischarge outcomes of the babies after mothers are discharged from the hospital within the first week, especially within the KCMC hospital catchment area. Future extensions include ways of handling missing values before applying the ML algorithms to predict perinatal death and other adverse pregnancy outcomes.

## Conclusion

The ML models (except for bagging) performed equally with the Lreg model to predict perinatal deaths using maternal, paternal and obstetric factors in this cohort. The ML models, however, have a higher net benefit, demonstrating superiority in the prediction of perinatal death. Furthermore, the RF model also demonstrated superior performance over other ML models. These models are a useful and alternative strategy over the standard Lreg model to predict perinatal deaths, considering the richness of the medical birth registries. Moreover, the ML models are capable of handling many predictors at the same time, which is crucial in capturing multiple risk factors for adverse perinatal outcomes such as perinatal deaths. The application of ML models may, therefore, increase the prediction ability of adverse perinatal outcomes and thereby helping in triage women most at risk.

## Supplementary Material

Reviewer comments

Author's manuscript

## References

[1] United Nations Development Program Sustainable development goals: United nations, 2019 Available: https://www.undp.org/content/undp/en/home/sustainable-development-goals/goal-3-good-health-and-well-being.html#targets [Accessed 14 Aug 2019].

[2] World Health Organization The WHO application of ICD-10 to deaths during the perinatal period: ICD-PM. Geneva: World Health Organization, 2016.

[3] World Health Organization Every newborn: an action plan to end preventable deaths. Geneva: World Health Organization, 2014.

[4] Hug L, Alexander M, You D, et al National, regional, and global levels and trends in neonatal mortality between 1990 and 2017, with scenario-based projections to 2030: a systematic analysis. Lancet Glob Health 2019;7:e710–20. 10.1016/S2214-109X(19)30163-9 31097275 PMC6527519

[5] Burstein R, Henry NJ, Collison ML, et al Mapping 123 million neonatal, infant and child deaths between 2000 and 2017. Nature 2019;574:353–8. 10.1038/s41586-019-1545-0 31619795 PMC6800389

[6] UNICEF Levels & Trends in Child Mortality: Report 2019. Estimates Developed by the UN Inter-agency Group for Child Mortality Estimation. United Nations Children’s Fund, 2019.

[7] Akombi BJ, Renzaho AM Perinatal mortality in sub-Saharan Africa: a meta-analysis of demographic and health surveys. Ann Glob Health 2019;85:2348. 10.5334/aogh.2348 PMC6634369 31298820

[8] MoHCDGEC, MoH NBS Tanzania demographic and health survey and malaria indicator survey (TDHS-MIS) 2015-16. Dar es Salaam, Tanzania and Rockville, Maryland, USA, 2016.

[9] Kuhle S, Maguire B, Zhang H, et al Comparison of logistic regression with machine learning methods for the prediction of fetal growth abnormalities: a retrospective cohort study. BMC Pregnancy Childbirth 2018;18:1–9. 10.1186/s12884-018-1971-2 30111303 PMC6094446

[10] Raita Y, Goto T, Faridi MK, et al Emergency department triage prediction of clinical outcomes using machine learning models. Crit Care 2019;23:64. 10.1186/s13054-019-2351-7 30795786 PMC6387562

[11] Lamping F, Jack T, Rübsamen N, et al Development and validation of a diagnostic model for early differentiation of sepsis and non-infectious SIRS in critically ill children - a data-driven approach using machine-learning algorithms. BMC Pediatr 2018;18:112. 10.1186/s12887-018-1082-2 29544449 PMC5853156

[12] Nasejje JB, Mwambi H Application of random survival forests in understanding the determinants of under-five child mortality in Uganda in the presence of covariates that satisfy the proportional and non-proportional hazards assumption. BMC Res Notes 2017;10:459. 10.1186/s13104-017-2775-6 28882171 PMC5590231

[13] Houweling TAJ, van Klaveren D, Das S, et al A prediction model for neonatal mortality in low- and middle-income countries: an analysis of data from population surveillance sites in India, Nepal and Bangladesh. Int J Epidemiol 2019;48:186–98. 10.1093/ije/dyy194 30325465 PMC6380321

[14] Hoodbhoy Z, Noman M, Shafique A, et al Use of machine learning algorithms for prediction of fetal risk using cardiotocographic data. Int J Appl Basic Med Res 2019;9:226. 10.4103/ijabmr.IJABMR_370_18 31681548 PMC6822315

[15] Muktan D, Singh RR, Bhatta NK, et al Neonatal mortality risk assessment using SNAPPE- II score in a neonatal intensive care unit. BMC Pediatr 2019;19:279. 10.1186/s12887-019-1660-y 31409303 PMC6691535

[16] Lee SM, Lee MH, Chang YS, et al The clinical risk index for babies II for prediction of time-dependent mortality and short-term morbidities in very low birth weight infants. Neonatology 2019;116:244–51. 10.1159/000500270 31307048

[17] Goto T, Camargo CA, Faridi MK, et al Machine Learning-Based prediction of clinical outcomes for children during emergency department triage. JAMA Netw Open 2019;2:e186937. 10.1001/jamanetworkopen.2018.6937 30646206 PMC6484561

[18] Vellido A, Ribas V, Morales C, et al Machine learning in critical care: state-of-the-art and a sepsis case study. Biomed Eng Online 2018;17:135. 10.1186/s12938-018-0569-2 30458795 PMC6245501

[19] Christodoulou E, Ma J, Collins GS, et al A systematic review shows no performance benefit of machine learning over logistic regression for clinical prediction models. J Clin Epidemiol 2019;110:12–22. 10.1016/j.jclinepi.2019.02.004 30763612

[20] Allanson E, Tunçalp Özge, Gardosi J, et al Classifying the causes of perinatal death. Bull World Health Organ 2016;94:79–79A. 10.2471/BLT.15.168047 26908954 PMC4750440

[21] Wojcieszek AM, Reinebrant HE, Leisher SH, et al Characteristics of a global classification system for perinatal deaths: a Delphi consensus study. BMC Pregnancy Childbirth 2016;16:223. 10.1186/s12884-016-0993-x 27527704 PMC4986199

[22] Pitt C, Tawiah T, Soremekun S, et al Cost and cost-effectiveness of newborn home visits: findings from the Newhints cluster-randomised controlled trial in rural Ghana. Lancet Glob Health 2016;4:e45–56. 10.1016/S2214-109X(15)00207-7 26639857 PMC5357735

[23] World Health Organization Newborns: reducing mortality, 2020 Available: https://www.who.int/news-room/fact-sheets/detail/newborns-reducing-mortality [Accessed 02 May 2020].

[24] Khan FA, Mullany LC, Wu LF-S, et al Predictors of neonatal mortality: development and validation of prognostic models using prospective data from rural Bangladesh. BMJ Glob Health 2020;5:e001983. 10.1136/bmjgh-2019-001983 PMC7042570 32133171

[25] Chaibva BV, Olorunju S, Nyadundu S, et al Adverse pregnancy outcomes, 'stillbirths and early neonatal deaths' in Mutare district, Zimbabwe (2014): a descriptive study. BMC Pregnancy Childbirth 2019;19:86. 10.1186/s12884-019-2229-3 30841873 PMC6402130

[26] Mmbaga BT, Lie RT, Kibiki GS, et al Transfer of newborns to neonatal care unit: a Registry based study in northern Tanzania. BMC Pregnancy Childbirth 2011;11:68. 10.1186/1471-2393-11-68 21970789 PMC3206461

[27] Mitao M, Philemon R, Obure J, et al Risk factors and adverse perinatal outcome associated with low birth weight in northern Tanzania: a registry-based retrospective cohort study. Asian Pacific Journal of Reproduction 2016;5:75–9. 10.1016/j.apjr.2015.12.014

[28] Mahande MJ, Daltveit AK, Obure J, et al Recurrence of preterm birth and perinatal mortality in northern Tanzania: registry-based cohort study. Trop Med Int Health 2013;18:962–7. 10.1111/tmi.12111 23581495 PMC3749445

[29] Mahande MJ, Daltveit AK, Mmbaga BT, et al Recurrence of perinatal death in northern Tanzania: a Registry based cohort study. BMC Pregnancy Childbirth 2013;13:166. 10.1186/1471-2393-13-166 23988153 PMC3765768

[30] Isaksen AB, Østbye T, Mmbaga BT, et al Alcohol consumption among pregnant women in northern Tanzania 2000-2010: a registry-based study. BMC Pregnancy Childbirth 2015;15:205. 10.1186/s12884-015-0630-0 26337194 PMC4559883

[31] Chuwa FS, Mwanamsangu AH, Brown BG, et al Maternal and fetal risk factors for stillbirth in northern Tanzania: a registry-based retrospective cohort study. PLoS One 2017;12:e0182250. 10.1371/journal.pone.0182250 28813528 PMC5557599

[32] Hamilton EF, Dyachenko A, Ciampi A, et al Estimating risk of severe neonatal morbidity in preterm births under 32 weeks of gestation. J Matern Fetal Neonatal Med 2020;33:73–80. 10.1080/14767058.2018.1487395 29886760

[33] Mahande AM, Mahande MJ Prevalence of parasitic infections and associations with pregnancy complications and outcomes in northern Tanzania: a registry-based cross-sectional study. BMC Infect Dis 2016;16:78. 10.1186/s12879-016-1413-6 26874788 PMC4753041

[34] Temu TB, Masenga G, Obure J, et al Maternal and obstetric risk factors associated with preterm delivery at a referral hospital in northern-eastern Tanzania. Asian Pacific Journal of Reproduction 2016;5:365–70. 10.1016/j.apjr.2016.07.009

[35] Bergsjø P, Mlay J, Lie RT, et al A medical birth registry at Kilimanjaro Christian medical centre. East Afr J Public Health 2007;4:1–4. 17907753

[36] Mahande MJ Recurrence of perinatal death, preterm birth and preeclampsia in northern Tanzania: a Registry based study. University of Bergen, 2015.

[37] World Health Organization Maternal and perinatal health: World Health organization, 2020 Available: http://www.who.int/maternal_child_adolescent/topics/maternal/maternal_perinatal/en/ [Accessed 02 Mar 2020].

[38] Nijkamp JW, Sebire NJ, Bouman K, et al Perinatal death investigations: what is current practice? Semin Fetal Neonatal Med 2017;22:167–75. 10.1016/j.siny.2017.02.005 28325580 PMC7118457

[39] Getiye Y, Fantahun M Factors associated with perinatal mortality among public health deliveries in Addis Ababa, Ethiopia, an unmatched case control study. BMC Pregnancy Childbirth 2017;17:245. 10.1186/s12884-017-1420-7 28747161 PMC5530490

[40] Allanson ER, Muller M, Pattinson RC Causes of perinatal mortality and associated maternal complications in a South African Province: challenges in predicting poor outcomes. BMC Pregnancy Childbirth 2015;15:37. 10.1186/s12884-015-0472-9 25880128 PMC4339432

[41] Vogel JP, Souza JP, Mori R, et al Maternal complications and perinatal mortality: findings of the world Health organization multicountry survey on maternal and newborn health. BJOG 2014;121 Suppl 1:76–88. 10.1111/1471-0528.12633 24641538

[42] Unterscheider J, O'Donoghue K, Daly S, et al Fetal growth restriction and the risk of perinatal mortality-case studies from the multicentre Porto study. BMC Pregnancy Childbirth 2014;14:63. 10.1186/1471-2393-14-63 24517273 PMC3923738

[43] Mpembeni R, Jonathan R, Mughamba J Perinatal mortality and associated factors among deliveries in three municipal hospitals of Dar ES Salaam, Tanzania. Journal of Pediatrics & Neonatal Care 2014;1.

[44] Ouyang F, Zhang J, Betrán AP, et al Recurrence of adverse perinatal outcomes in developing countries. Bull World Health Organ 2013;91:357–67. 10.2471/BLT.12.111021 23678199 PMC3646344

[45] Mutsaerts MAQ, Groen H, Buiter-Van der Meer A, et al Effects of paternal and maternal lifestyle factors on pregnancy complications and perinatal outcome. A population-based birth-cohort study: the gecko Drenthe cohort. Hum Reprod 2014;29:824–34. 10.1093/humrep/deu006 24510962

[46] WHO Preterm birth: World Health organization, 2020 Available: http://www.who.int/news-room/fact-sheets/detail/preterm-birth [Accessed 19 Feb 2020].

[47] World Health Organization WHA global nutrition targets 2025: low birth weight policy brief. Geneva: World Health Organization, 2014.

[48] StataCorp Stata statistical software: release 15. College Station, TX: StataCorp LLC, 2017.

[49] R Core Team R: a language and environment for statistical computing. Vienna, Austria: R Foundation for Statistical Computing, 2020.

[50] Dwivedi AK Artificial neural network model for effective cancer classification using microarray gene expression data. Neural Computing and Applications 2018;29:1545–54. 10.1007/s00521-016-2701-1

[51] Stephens D, Diesing M A comparison of supervised classification methods for the prediction of substrate type using multibeam acoustic and legacy grain-size data. PLoS One 2014;9:e93950. 10.1371/journal.pone.0093950 24699553 PMC3974812

[52] Breiman L Random forests. Mach Learn 2001;45:5–32. 10.1023/A:1010933404324

[53] Hastie T, Tibshirani R, Friedman J The elements of statistical learning: data mining, inference, and prediction. 2 edn Springer, 2009.

[54] IEEE Bayesian networks classifiers for gene-expression data. 2011 11th International Conference on intelligent systems design and applications, 2011.

[55] Musa AB Gene expression data classification with kernel independent component analysis. Research Journal of Mathematical and Statistical Sciences 2014;2320:6047.

[56] Dreiseitl S, Ohno-Machado L Logistic regression and artificial neural network classification models: a methodology review. J Biomed Inform 2002;35:352–9. 10.1016/S1532-0464(03)00034-0 12968784

[57] Sutton CD Classification and regression trees, Bagging, and boosting. data mining and data visualization, 2005: 303–29.

[58] Johnson JM, Khoshgoftaar TM Survey on deep learning with class imbalance. Journal of Big Data 2019;6:27 10.1186/s40537-019-0192-5

[59] Chawla NV Data mining for imbalanced datasets: an overview. data mining and knowledge discovery Handbook. Springer, 2009: 875–86.

[60] Vickers AJ, Elkin EB Decision curve analysis: a novel method for evaluating prediction models. Med Decis Making 2006;26:565–74. 10.1177/0272989X06295361 17099194 PMC2577036

[61] Zhang Z, Rousson V, Lee W-C, et al Decision curve analysis: a technical note. Ann Transl Med 2018;6:308 10.21037/atm.2018.07.02 30211196 PMC6123195

[62] Khandwala YS, Baker VL, Shaw GM, et al Association of paternal age with perinatal outcomes between 2007 and 2016 in the United States: population based cohort study. BMJ 2018;363:k4372. 10.1136/bmj.k4372 30381468 PMC6207919

[63] Tough SC, Faber AJ, Svenson LW, et al Is paternal age associated with an increased risk of low birthweight, preterm delivery, and multiple birth? Can J Public Health 2003;94:88–92. 10.1007/BF03404578 12675162 PMC6979898

[64] Meng Y, Groth SW Fathers count: the impact of paternal risk factors on birth outcomes. Matern Child Health J 2018;22:401–8. 10.1007/s10995-017-2407-8 29218490 PMC5892832

[65] Hurley EG, DeFranco EA Influence of paternal age on perinatal outcomes. Am J Obstet Gynecol 2017;217:566.e1–566.e6. 10.1016/j.ajog.2017.07.034 28784418

[66] Peneza AK, Maluka SO 'Unless you come with your partner you will be sent back home': strategies used to promote male involvement in antenatal care in southern Tanzania. Glob Health Action 2018;11:1449724. 10.1080/16549716.2018.1449724 29699464 PMC5933283

[67] Gibore NS, Bali TAL Community perspectives: an exploration of potential barriers to men's involvement in maternity care in a central Tanzanian community. PLoS One 2020;15:e0232939. 10.1371/journal.pone.0232939 32437360 PMC7241761

[68] Carvalho CA, Silva AAMda, Victora C, et al Changes in infant and neonatal mortality and associated factors in eight cohorts from three Brazilian cities. Sci Rep 2020;10 10.1038/s41598-020-59910-7 PMC7039903 32094364

[69] Habimana-Kabano I, Broekhuis A, Hooimeijer P The effects of Interpregnancy intervals and previous pregnancy outcome on fetal loss in Rwanda (1996–2010). Int J Reprod Med 2015;2015:1–10. 10.1155/2015/413917 PMC4647053 26613103

[70] Flores Navarro-Pérez C, González-Jiménez E, Schmidt-RioValle J, et al [SOCIODEMOGRAPHIC FACTORS AND ADEQUACY OF PRENATAL CARE ASSOCIATED PERINATAL MORTALITY IN COLOMBIAN PREGNANT WOMEN]. Nutr Hosp 2015;32:1091–8. 10.3305/nh.2015.32.3.9179 26319825

[71] Ali AAA, Elgessim ME, Taha E, et al Factors associated with perinatal mortality in Kassala, eastern Sudan: a community-based study 2010-2011. J Trop Pediatr 2014;60:79–82. 10.1093/tropej/fmt075 24052575

[72] Nankabirwa V, Tumwine JK, Tylleskär T, et al Perinatal mortality in eastern Uganda: a community based prospective cohort study. PLoS One 2011;6:e19674. 10.1371/journal.pone.0019674 21573019 PMC3090412

[73] Hinderaker SG, Olsen BE, Bergsjø PB, et al Perinatal mortality in northern rural Tanzania. J Health Popul Nutr 2003;21:8–17. 12751669

[74] Blagus R, Lusa L SMOTE for high-dimensional class-imbalanced data. BMC Bioinformatics 2013;14:106. 10.1186/1471-2105-14-106 23522326 PMC3648438

[75] Blagus R, Lusa L Improved shrunken centroid classifiers for high-dimensional class-imbalanced data. BMC Bioinformatics 2013;14:64. 10.1186/1471-2105-14-64 23433084 PMC3687811

