# Supplementary material for: Prediction of perinatal death using machine learning models: a birth registry-based cohort study in northern Tanzania
Source: BMJ Open. 2020 Oct 19;10(10):e040132. doi: 10.1136/bmjopen-2020-040132 (PMC7574940; doi:10.1136/bmjopen-2020-040132)
Supplement: Supplementary data [file bmjopen-2020-040132supp001.pdf]

```
# input Stata file
library(foreign)
data<-read.dta("D:/Documents/Innocent/PhD 2018/PhD project/Data/Results/Machine Learning Paper/complete_data_withlabels.dta")
utils::View(head(data))
summary(data)
##################################################################################
#Divide data into training and testing sets

library(caret)

set.seed(100) # For reproducabilty

trainIndex <- createDataPartition(data$perinatal_death,p=.70,list=FALSE) #divide the data based on the outcome variable in the same ratio
trainData <- data[trainIndex,]
testData  <- data[-trainIndex,]
table(data$perinatal_death)
table(trainData$perinatal_death)
table(testData$perinatal_death)
##################################################################################

#Variable/Feature Selection
##################################################################################

# install.packages("mlbench")
# install.packages("Hmisc")
# install.packages("randomForest")

#RandomForests
#install.packages("caret", "mlbench", "mlbench", "randomForest")

library(mlbench)
library(Hmisc)
library(randomForest)
library(caret)

#speed up the performance of the analysis
# library(doParallel)
# ncores <- detectCores() - 1
# cl <- makeCluster(ncores)
# registerDoParallel(cl)

#Parameter tuning and Cross validation
```

```
ctrl <- trainControl(method="CV",   # 10fold cross validation
                number = 10,
                #repeats = 10, #has no meaning for this resampling method
                savePredictions = TRUE,
                classProbs=TRUE,
                allowParallel = TRUE,
                summaryFunction = twoClassSummary
                #search = "random" # Random searching of hyperparameters?
                )

#Feature selection using random forests algorithm
RFMod <- train(x=trainData[,-which(names(trainData) %in% c("perinatal_death"))],
            y=trainData[,which(names(trainData) %in% c("perinatal_death"))],
            method="rf", metric="ROC", trControl=ctrl)

#--------------------------------------------------------------------------
#select and plot the Important variables from the RF model
RFImp <- varImp(RFMod, scale=T)
RFImp
plot(RFImp, main='Variable Importance')
plot(RFImp, top = 20, main='Variable Importance') #all 20 selected features

#Plot of important features with proper names
impvarsdata<-data.frame(impvars=c("Year of birth", "Paternal age groups", "Birth weight",
  "Paternal education level", "Maternal education level", "Maternal occupation",
  "Maternal age groups", "Sex of the child","Mode of delivery", "Number of ANC visits",
  "Area of residence", "Alcohol use during pregnancy", "Paternal occupation",
  "Gestational age at birth", "Malaria during pregnancy", "Induction of labor",
  "Maternal marital status", "Abruption placenta",  "Referral status",
  "Pre-eclampsia/ eclampsia"),

  rfvalues=c(100.00,      41.12,   31.51,   28.49,   26.82,  26.32,    26.18,   25.00,   22.22,
          21.13,    19.53,   19.39,   17.64,   17.29,   14.31,  13.96,    12.57,   11.96,
          11.11,    10.69))

utils::View(impvarsdata) #compare with RF below
RFImp
class(impvarsdata$rfvalues)
class(impvarsdata$impvars)

# arrange in descending order
# https://rpubs.com/Felix/7644

impvarsdata$impvars <- factor(impvarsdata$impvars,
```

```
                 levels = impvarsdata[order(impvarsdata$rfvalues), "impvars"])

ggplot(impvarsdata, aes(x = impvars, y = rfvalues)) + geom_bar(stat = "identity") +
  coord_flip() +  xlab("Predictors") + ylab("Importance") +
  theme(axis.text = element_text(colour = "black")) +
  theme(axis.line = element_line(colour = "black"))

ggplot(RFImp, top = 20, main='Variable Importance') #all 20 selected features
#--------------------------------------------------------------------------

# Remove the variables which were not selected from both training and testing datasets

#training
trainDataIMP <- trainData[, c("year", "fage_grp", "lbw", "feducation", "meducation2", "moccup_grp2",
"mage_grp2", "sex2", "demode_grp2", "ancvisit_grp", "residence_grp2", "alcopreg2", "PTB_LMP_grp",
"foccup_grp", "INDUCT", "MHDP10", "marstat_grp2", "CMPL04", "refdel2", "PE_E")]
trainDataIMP$perinatal_death <- trainData$perinatal_death[match(row.names(trainDataIMP),
row.names(trainData))]

#testing
testDataIMP <- testData[,c("year", "fage_grp", "lbw", "feducation", "meducation2", "moccup_grp2",
"mage_grp2", "sex2", "demode_grp2", "ancvisit_grp", "residence_grp2", "alcopreg2", "PTB_LMP_grp",
"foccup_grp", "INDUCT", "MHDP10", "marstat_grp2", "CMPL04", "refdel2",  "PE_E")]
testDataIMP$perinatal_death <- testData$perinatal_death[match(row.names(testDataIMP),
row.names(testData))]

#save both data to CSV
write.csv(x = trainDataIMP, file = "D:/Documents/Innocent/PhD 2018/PhD
project/Data/Results/Machine Learning Paper/trainDataIMP.csv", row.names = TRUE)
write.csv(x = testDataIMP, file = "D:/Documents/Innocent/PhD 2018/PhD project/Data/Results/Machine
Learning Paper/testDataIMP.csv", row.names = TRUE)

#Classification - use selected variables (from training data) to predict perinatal death (in testing data)

#Reload the saved data
TrainData <- read.csv(file = "D:/Documents/Innocent/PhD 2018/PhD project/Data/Results/Machine
Learning Paper/trainDataIMP.csv", header = TRUE, row.names = 1, stringsAsFactors = TRUE)
TestData <- read.csv(file = "D:/Documents/Innocent/PhD 2018/PhD project/Data/Results/Machine
Learning Paper/testDataIMP.csv", header = TRUE, row.names = 1, stringsAsFactors = TRUE)
################################################################################

# 1) Logistic regression
################################################################################
library(DMwR)
```

```
ctrlLReg <- trainControl(## 10-fold CV
  method = "cv",
  number = 10,
  classProbs=TRUE,
  savePredictions = TRUE,
  allowParallel = TRUE,
  sampling = "smote",
  # To correct for class imbalances (more alive than death in perinatal_death)
  # https://www.r-bloggers.com/dealing-with-unbalanced-data-in-machine-learning/
  summaryFunction = twoClassSummary
)

# Train
set.seed(1239)
lreg <- train(x=TrainData[-which(names(TrainData) %in% c("perinatal_death"))],
            y= TrainData$perinatal_death, method="glm",family=binomial(),
            trControl=ctrlLReg)

lreg
#-------------------------------------------------------------

# Test
set.seed(1239)
lreg.pred <- predict(lreg, TestData[,-which(names(TestData) %in% c("perinatal_death"))])
lreg.tab = table(pred = lreg.pred, true = TestData[,c("perinatal_death")])
lreg.Conf = confusionMatrix(lreg.pred, TestData[,c("perinatal_death")], positive =
levels(TestData[,c("perinatal_death")])[2])
lreg.Conf

##################################################################################

# 2) Naive Bayes
##################################################################################

# Load required packages
library(dplyr)        # Used by caret
#library(nnet)      # support vector machine
library(pROC)          # plot the ROC curves
#library(e1071)
# install.packages("naivebayes")
library(naivebayes)
```

```
set.seed(1236)
# Setup for cross validation
ctrlNB <- trainControl(method="CV",   # 10fold cross validation
                     number = 10,
                     savePredictions = TRUE,
                     classProbs=TRUE,
                     allowParallel = TRUE,
                     sampling = "smote",
                     summaryFunction = twoClassSummary
)

set.seed(1236)

# Train
NBModel.tune <- train(x=TrainData[-which(names(TrainData) %in% c("perinatal_death"))],
                    y= TrainData$perinatal_death,
                    method = "naive_bayes",   # Naive Bayes
                    #tuneLength = 5,                                              # 9 values of the cost function
                    #preProc = c("center","scale"),  # Center and scale data
                      #not needed for this analysis
                    metric="ROC",
                    trControl=ctrlNB)

NBModel.tune
plot(NBModel.tune)

#-------------------------------------------------------------
# Test
NBModel.pred <- predict(NBModel.tune, TestData[,-which(names(TestData) %in%
c("perinatal_death"))])
NBModel.tab = table(pred = NBModel.pred, true = TestData[,c("perinatal_death")])
NBModel.Conf = confusionMatrix(NBModel.pred, TestData[,c("perinatal_death")], positive =
levels(TestData[,c("perinatal_death")])[2])
NBModel.Conf
##############################################################################

# 3) Artificial nueral network (ANN)
##############################################################################

# Load important packages
# library(caret)
# library(dplyr)        # Used by caret
library(nnet)       # support vector machine
# library(pROC)        # plot the ROC curves
```

```
set.seed(1234)
#set.seed(123)
# Setup for cross validation
ctrlANN <- trainControl(method="CV",   # 10fold cross validation
                  number = 10,
                  #savePredictions = TRUE,
                  classProbs=TRUE,
                  allowParallel = TRUE,
                  savePredictions = TRUE,
                  sampling = "smote",
                  summaryFunction = twoClassSummary
)

# Train: Artificial nueral network (ANN) model
set.seed(1234)
ANNModel.tune <- train(x=TrainData[-which(names(TrainData) %in% c("perinatal_death"))],
                 y= TrainData$perinatal_death,
                 method = "nnet",   # nueral network
                 #tuneLength = 5,                                    # 9 values of the cost function
                 #preProc = c("center","scale"),  # Center and scale data
                 #metric="ROC",
                 #linout=TRUE,
                 #maxit = 1000,
                 #tuneGrid = my.grid,
                 trace = F,
                 #tuneLength = 10,
                 #linout = 1,
                 trControl=ctrlANN,
                 metric="ROC")

ANNModel.tune
plot(ANNModel.tune)
#------------------------------------------------------------

# Test
set.seed(1234)
ANNModel.pred <- predict(ANNModel.tune, TestData[,-which(names(TestData) %in%
c("perinatal_death"))])
ANNModel.tab = table(pred = ANNModel.pred, true = TestData[,c("perinatal_death")])
ANNModel.Conf = confusionMatrix(ANNModel.pred, TestData[,c("perinatal_death")],
                   positive = levels(TestData[,c("perinatal_death")])[2])
ANNModel.Conf
###############################################################################
```

```
# 4) Bagged Tree
##################################################################################
# Specify the training configuration
ctrlBagTree <- trainControl(method = "cv",
                       number = 10,
                       classProbs=TRUE,
                       savePredictions = TRUE,
                       allowParallel = TRUE,
                       sampling = "smote",
                       summaryFunction = twoClassSummary)  # For AUC

# Cross validate the credit model using "treebag" method;
# Track AUC (Area under the ROC curve)
set.seed(1237)  # for reproducibility
BaggedTree <- train(x=TrainData[-which(names(TrainData) %in% c("perinatal_death"))],
                y= TrainData$perinatal_death,
                method = "treebag",
                metric = "ROC",
                trControl = ctrlBagTree)
BaggedTree

#------------------------------------------------------------
# Test
set.seed(1237)
BaggedTree.pred <- predict(BaggedTree, TestData[,-which(names(TestData) %in%
c("perinatal_death"))])
BaggedTree.tab = table(pred = BaggedTree.pred, true = TestData[,c("perinatal_death")])
BaggedTree.Conf = confusionMatrix(BaggedTree.pred, TestData[,c("perinatal_death")], positive =
levels(TestData[,c("perinatal_death")])[2])
# if you need to predict alive change to 1 at the end of the syntax
BaggedTree.Conf
##################################################################################

# 5) Boosting
##################################################################################
#install gbm package
# install.packages("gbm")
library(gbm)
# Specify the training configuration
ctrlBoosting <- trainControl(method = "cv",
                        number = 10,
                        classProbs=TRUE,
                        savePredictions = TRUE,
```

```
                       allowParallel = TRUE,
                       sampling = "smote",
                       summaryFunction = twoClassSummary)  # For AUC

# Cross validate the credit model using "treebag" method;
# Track AUC (Area under the ROC curve)
set.seed(1238)  # for reproducibility
Boosting <- train(x=TrainData[-which(names(TrainData) %in% c("perinatal_death"))],
              y= TrainData$perinatal_death,
              method = "gbm",
              metric = "ROC",
              trControl = ctrlBoosting)
plot(Boosting)

#--------------------------------------------------------------
# Test
set.seed(1238)
Boosting.pred <- predict(Boosting, TestData[,-which(names(TestData) %in% c("perinatal_death"))])
Boosting.tab = table(pred = Boosting.pred, true = TestData[,c("perinatal_death")])
Boosting.Conf = confusionMatrix(Boosting.pred, TestData[,c("perinatal_death")], positive =
levels(TestData[,c("perinatal_death")])[2])
Boosting.Conf
##################################################################################

# 6) Random Forests (RF)
##################################################################################
#Load additional libraries
library(caTools)
library(mlbench)

#set.seed(123)
# Setup for cross validation
ctrlRF <- trainControl(method="CV",   # 10fold cross validation
                  number = 10,
                  savePredictions = TRUE,
                  classProbs=TRUE,
                  allowParallel = TRUE,
                  sampling = "smote",
                  summaryFunction = twoClassSummary
                  )

#Train
set.seed(1235)
RFModel.tune <- train(x=TrainData[-which(names(TrainData) %in% c("perinatal_death"))],
```

```
                y= TrainData$perinatal_death,
                method = "rf",   # Random Forest
                #tuneLength = 5,                                          # 9 values of the cost function
                #preProc = c("center","scale"),  # Center and scale data
                metric="ROC",
                trControl=ctrlRF)

RFModel.tune
plot(RFModel.tune)

#-------------------------------------------------------------
# Test
set.seed(1235)
RFModel.pred <- predict(RFModel.tune, TestData[,-which(names(TestData) %in% c("perinatal_death"))])
RFModel.tab = table(pred = RFModel.pred, true = TestData[,c("perinatal_death")])
RFModel.Conf = confusionMatrix(RFModel.pred, TestData[,c("perinatal_death")], positive =
levels(TestData[,c("perinatal_death")])[2])
RFModel.Conf
################################################################################

# RESULTS
################################################################################

# Plot ROC curves
# ============================================================================
library(ROCR)
probANN <- predict(ANNModel.tune, newdata=TestData[,-which(names(TestData) %in%
c("perinatal_death"))], type="prob")
predANN <- prediction(probANN[,2], TestData[,c("perinatal_death")])
perfANN <- performance(predANN, measure = "tpr", x.measure = "fpr")
plot(perfANN, col="Black", lwd=2)

probRF <- predict(RFModel.tune, newdata=TestData[,-which(names(TestData) %in%
c("perinatal_death"))], type="prob")
predRF <- prediction(probRF[,2], TestData[,c("perinatal_death")])
perfRF <- performance(predRF, measure = "tpr", x.measure = "fpr")
plot(perfRF, col="Blue", lwd=2, add = TRUE)

probNB <- predict(NBModel.tune, newdata=TestData[,-which(names(TestData) %in%
c("perinatal_death"))], type="prob")
predNB <- prediction(probNB[,2], TestData[,c("perinatal_death")])
perfNB <- performance(predNB, measure = "tpr", x.measure = "fpr")
plot(perfNB, col="Green", lwd=2, add = TRUE)
```

```
probLreg <- predict(lreg, newdata=TestData[,-which(names(TestData) %in% c("perinatal_death"))],
type="prob")
predLreg <- prediction(probLreg[,2], TestData[,c("perinatal_death")])
perfLreg <- performance(predLreg, measure = "tpr", x.measure = "fpr")
plot(perfLreg, col="Red", lwd=2, add = TRUE)

probBaggedTrees <- predict(BaggedTree, newdata=TestData[,-which(names(TestData) %in%
c("perinatal_death"))], type="prob")
predBaggedTrees <- prediction(probBaggedTrees[,2], TestData[,c("perinatal_death")])
perfBaggedTrees <- performance(predBaggedTrees, measure = "tpr", x.measure = "fpr")
plot(perfBaggedTrees, col="Orange", lwd=2, add = TRUE)

probBoosting <- predict(Boosting, newdata=TestData[,-which(names(TestData) %in%
c("perinatal_death"))], type="prob")
predBoosting <- prediction(probBoosting[,2], TestData[,c("perinatal_death")])
perfBoosting <- performance(predBoosting, measure = "tpr", x.measure = "fpr")
plot(perfBoosting, col="DarkGray", lwd=2, add = TRUE)

#all plots together
par(mar=c(5,5,1,1), cex=0.9) # cex = text font size, mar=margins (left, bottom, top, right)
plot(perfANN, col="Black", lwd=2)
plot(perfRF, col="Blue", lwd=2, add = TRUE)
plot(perfNB, col="Green", lwd=2, add = TRUE)
plot(perfLreg, col="Red", lwd=2, add = TRUE)
plot(perfBaggedTrees, col="Orange", lwd=2, add = TRUE)
plot(perfBoosting, col="DarkGray", lwd=2, add = TRUE)

legend(0.55,0.7, legend = c("Artificial neural networks", "Random forest", "Naive bayes",
                      "Logistic regression", "Bagging", "Boosting"),
      col = c("Black", "Blue", "Green", "Red", "Orange", "DarkGray"),
      lty = 1,lwd = 3,bty = "n", y.intersp = 1.5, cex=0.8)
# ==================================================================

#Compute AUC values
#==================================================================
ANN_auc <- performance(predANN, measure = "auc")
ANN_auc <- ANN_auc@y.values[[1]]

RF_auc <- performance(predRF, measure = "auc")
RF_auc <- RF_auc@y.values[[1]]

NB_auc <- performance(predNB , measure = "auc")
NB_auc <- NB_auc@y.values[[1]]
```

```
Lreg_auc <- performance(predLreg , measure = "auc")
Lreg_auc <- Lreg_auc@y.values[[1]]

Bagged_auc <- performance(predBaggedTrees , measure = "auc")
Bagged_auc <- Bagged_auc@y.values[[1]]

Boosting_auc <- performance(predBoosting , measure = "auc")
Boosting_auc <- Boosting_auc@y.values[[1]]

#an alternative and short way compared to the above syntax
aucANN<-auc(as.numeric(TestData$perinatal_death), probANN[,2])#ANN
aucRF<-auc(as.numeric(TestData$perinatal_death), probRF[,2])#RF
aucNB<-auc(as.numeric(TestData$perinatal_death), probNB[,2])#NB
aucLreg<-auc(as.numeric(TestData$perinatal_death), probLreg[,2])#Lreg
aucBagging<-auc(as.numeric(TestData$perinatal_death), probBaggedTrees[,2])#Bagging
aucBoosting<-auc(as.numeric(TestData$perinatal_death), probBoosting[,2])#Boosting
#==========================================================================

#Compute CI for AUC
#==========================================================================
library(pROC)
ci.auc(as.numeric(TestData$perinatal_death), probANN[,2])#ANN
ci.auc(as.numeric(TestData$perinatal_death), probRF[,2])#RF
ci.auc(as.numeric(TestData$perinatal_death), probNB[,2])#NB
ci.auc(as.numeric(TestData$perinatal_death), probLreg[,2])#Lreg
ci.auc(as.numeric(TestData$perinatal_death), probBaggedTrees[,2])#Bagging
ci.auc(as.numeric(TestData$perinatal_death), probBoosting[,2])#Boosting
#==========================================================================

#Comparison of AUC for different machines using Delong's Test
library(ROCR)
roc.test(aucLreg, aucANN, method = "delong") #delong test is the default
roc.test(aucLreg, aucNB)
roc.test(aucLreg, aucBagging)
roc.test(aucLreg, aucBoosting)
roc.test(aucLreg, aucRF)
#==========================================================================
#Compute CT for Sens, Spec, Prevelance ... etc
#==========================================================================

library(epiR)
epi.tests(ANNModel.Conf$table, conf.level = 0.95)
epi.tests(RFModel.Conf$table, conf.level = 0.95)
epi.tests(NBModel.Conf$table, conf.level = 0.95)
```

```
epi.tests(lreg.Conf$table, conf.level = 0.95)
epi.tests(BaggedTree.Conf$table, conf.level = 0.95)
epi.tests(Boosting.Conf$table, conf.level = 0.95)

#=========================================================================

# Summary statistics of the Results
# =========================================================================
library(caret)
results <- resamples(list(ANN=ANNModel.tune, RF=RFModel.tune,
                          NB=NBModel.tune, BaggedTree=BaggedTree, Lreg=lreg, Boosting= Boosting))
# Table comparison
summary(results)

# boxplot comparison
bwplot(results)
# Dot-plot comparison
dotplot(results)
# =========================================================================

# Decision curve analysis
# DCA analysis
# =========================================================================
library("tidyverse")
library("dplyr")

#Predict Perinatal death
ANNModel.pred <- predict(ANNModel.tune, TestData[,-which(names(TestData) %in%
c("perinatal_death"))], type = "prob")
RFModel.pred <- predict(RFModel.tune, TestData[,-which(names(TestData) %in% c("perinatal_death"))],
type = "prob")
NBModel.pred <- predict(NBModel.tune, TestData[,-which(names(TestData) %in%
c("perinatal_death"))], type = "prob")
lreg.pred <- predict(lreg, TestData[,-which(names(TestData) %in% c("perinatal_death"))], type = "prob")
BaggedTree.pred <- predict(BaggedTree, TestData[,-which(names(TestData) %in%
c("perinatal_death"))], type = "prob")
Boosting.pred <- predict(Boosting, TestData[,-which(names(TestData) %in% c("perinatal_death"))], type
= "prob")

dcadata <- TestData %>%select(c(perinatal_death))
source("D:/Documents/Innocent/PhD 2018/PhD project/Data/Results/Machine Learning Paper/dca.r")
  # This file is avaible at http://www.decisioncurveanalysis.org
library(reshape2)
dcadata$ANN<-as.numeric(ANNModel.pred$Died)
```

```
dcadata$RF<-as.numeric(RFModel.pred$Died)
dcadata$NB<-as.numeric(NBModel.pred$Died)
dcadata$Lreg<-as.numeric(lreg.pred$Died)
dcadata$Bagging<-as.numeric(BaggedTree.pred$Died)
dcadata$Boosting <- as.numeric(Boosting.pred$Died)
data.set <- dcadata

attach(data.set)
data.set$perinatal_death<-as.numeric(data.set$perinatal_death)
data.set$perinatal_death<-data.set$perinatal_death-1 #0=alive, 1=died (in the testing data)

#Plot the DCA curves
library(DCA)
dca(data=data.set, outcome="perinatal_death",
    predictors=c("ANN", "RF", "NB", "Lreg", "Bagging", "Boosting"), xstart=0, ymin=0)

dcaoutput <- dca(data=data.set, outcome="perinatal_death",
             predictors=c("ANN", "RF", "NB", "Lreg", "Bagging", "Boosting"), xstart=0,xstop=0.3, ymin=0)

dcadf <- data.frame(dcaoutput$net.benefit)
temp <- melt(dcadf, id="threshold",
          measure=c("ANN", "RF", "NB", "Lreg", "Bagging", "Boosting"))

library(ggplot2)
ggplot(temp,
      aes(x=threshold,
          y=value,
          colour=variable,
          group=variable)) + geom_line() +
  geom_line(size=0.8) + # Thicker line
  coord_cartesian(xlim = c(0, 0.075), ylim= c(0, 0.04)) +
  labs(x="Threshold probability (%)") + labs(y="Net benefit") +
  theme_minimal()  + theme(legend.position = c(0.8, 0.8),
                  text = element_text(size=12), #text size
                  panel.grid.major = element_blank(), #grid lines
                  panel.grid.minor = element_blank(),
                  axis.line = element_line(colour = "black"), # solid line
                  axis.ticks = element_line(size = 1), #tick marks
                  panel.border = element_rect(linetype = "solid",
                                  fill = NA, colour = "black"), #axis boarders
                  axis.text = element_text(color = "black")) + #text color
  scale_color_manual(values = c("Black", "Blue", "Green", "Red", "Orange",
                        "DarkGray"), #Use same colors as with ROC curve
              labels = c("Artificial neural networks",
```

```
                        "Random forest",
                        "Naive bayes",
                        "Logistic regression",
                        "Bagging",
                        "Boosting")) + labs(color="")
# ===========================================================================

#Combining the a) ROC and b) DC plots on top of one another.

# install.packages("magick")
library(magick)

Figure3_roc<-image_read("D:/Documents/Innocent/PhD 2018/PhD project/Data/Results/Machine
Learning Paper/Figure3_roc.tiff")
Figure3_roc<-image_annotate(Figure3_roc, "a", font = 'Times', size = 30)
Figure3_roc
Figure3_deccurve2<-image_read("D:/Documents/Innocent/PhD 2018/PhD
project/Data/Results/Machine Learning Paper/Figure3_deccurve.tiff")
Figure3_deccurve2<-image_annotate(Figure3_deccurve2, "b", font = 'Times', size = 30)
Figure3_deccurve2

img <- c(Figure3_roc, Figure3_deccurve2) #combine the two images
# img <- image_scale(img) #size of the images
img
image_info(img)

img2<-image_append(image_scale(img), stack = TRUE) %>%
  image_border("grey", "1x1")
img2
image_info(img2)

#export the image to tiff format (better quality)
image_write(img2, path = "D:/Documents/Innocent/PhD 2018/PhD project/Data/Results/Machine
Learning Paper/Figure3.tiff",
      format = "tiff")
# ===========================================================================
```
