# Supplementary material for: Prediction of perinatal death using machine learning models: a birth registry-based cohort study in northern Tanzania
Source: BMJ Open. 2020 Oct 19;10(10):e040132. doi: 10.1136/bmjopen-2020-040132 (PMC7574940; doi:10.1136/bmjopen-2020-040132)
Supplement: Author's manuscript [file bmjopen-2020-040132.draft_revisions.pdf]

BMJ Open

BMJ Open is committed to open peer review. As part of this commitment we make the peer review history of every article we publish publicly available.

When an article is published we post the peer reviewers' comments and the authors' responses online. We also post the versions of the paper that were used during peer review. These are the versions that the peer review comments apply to.

The versions of the paper that follow are the versions that were submitted during the peer review process. They are not the versions of record or the final published versions. They should not be cited or distributed as the published version of this manuscript.

BMJ Open is an open access journal and the full, final, typeset and author-corrected version of record of the manuscript is available on our site with no access controls, subscription charges or pay-per-view fees (http://bmjopen.bmj.com).

If you have any questions on BMJ Open's open peer review process please email info.bmjopen@bmj.com

Prediction of perinatal death using machine learning models: A birth registry-based cohort study in northern Tanzania

Journal:	*BMJ Open*
Manuscript ID	bmjopen-2020-040132
Article Type:	Original research
Date Submitted by the Author:	05-May-2020
Complete List of Authors:	Mboya, Innocent; University of KwaZulu-Natal, School of Mathematics, Statistics, and Computer Science; Kilimanjaro Christian Medical University College, Epidemiology and Biostatistics<br>Mahande, Michael Johnson; Kilimanjaro Christian Medical University College, Epidemiology & Biostatistics<br>Mohammed, Mohanad ; University of KwaZulu-Natal, School of Mathematics, Statistics and Computer Science<br>Obure, Joseph; Kilimanjaro Christian Medical Centre, Obstetrics and Gynaecology<br>Mwambi, Henry; University of KwaZulu-Natal, School of Mathematics, Statistics and Computer Science
Keywords:	Reproductive medicine < GYNAECOLOGY, NEONATOLOGY, Prenatal diagnosis < OBSTETRICS, PERINATOLOGY

SCHOLARONE™
Manuscripts

I, the Submitting Author has the right to grant and does grant on behalf of all authors of the Work (as defined in the below author licence), an exclusive licence and/or a non-exclusive licence for contributions from authors who are: i) UK Crown employees; ii) where BMJ has agreed a CC-BY licence shall apply, and/or iii) in accordance with the terms applicable for US Federal Government officers or employees acting as part of their official duties; on a worldwide, perpetual, irrevocable, royalty-free basis to BMJ Publishing Group Ltd ("BMJ") its licensees and where the relevant Journal is co-owned by BMJ to the co-owners of the Journal, to publish the Work in this journal and any other BMJ products and to exploit all rights, as set out in our licence.

The Submitting Author accepts and understands that any supply made under these terms is made by BMJ to the Submitting Author unless you are acting as an employee on behalf of your employer or a postgraduate student of an affiliated institution which is paying any applicable article publishing charge ("APC") for Open Access articles. Where the Submitting Author wishes to make the Work available on an Open Access basis (and intends to pay the relevant APC), the terms of reuse of such Open Access shall be governed by a Creative Commons licence – details of these licences and which Creative Commons licence will apply to this Work are set out in our licence referred to above.

Other than as permitted in any relevant BMJ Author's Self Archiving Policies, I confirm this Work has not been accepted for publication elsewhere, is not being considered for publication elsewhere and does not duplicate material already published. I confirm all authors consent to publication of this Work and authorise the granting of this licence.

For peer review only

Prediction of perinatal death using machine learning models: A birth registry-based cohort study in northern Tanzania

Innocent B. Mboya[1,2*], Michael J. Mahande[2], Mohanad Mohammed[1], Joseph Obure[3], Henry G. Mwambi[1]

[1]School of Mathematics, Statistics, and Computer Science, University of KwaZulu-Natal, Pietermaritzburg, Private Bag X01, Scottsville 3209, South Africa.

[2]Department of Epidemiology and Biostatistics, Kilimanjaro Christian Medical University College (KCMUCo), P. O. Box 2240, Moshi-Tanzania.

[3]Department of Obstetrics and Gynecology, Kilimanjaro Christian Medical Center (KCMC), P. O. Box 3010, Moshi-Tanzania.

[*]Correspondence

E-mail: ib.mboya@gmail.com

For peer review only

ABSTRACT

Objective: We aimed to determine the key predictors of perinatal deaths using machine learning models compared with the logistic regression model.

Design: A secondary data analysis using the Kilimanjaro Christian Medical Center (KCMC) Medical Birth Registry cohort from 2000 to 2015. We assessed the discriminative ability of models using the area under the receiver operating characteristics curve (AUC) and the net benefit using decision curve analysis.

Setting: The KCMC is a zonal referral hospital located in Moshi Municipality, Kilimanjaro Region, Northern Tanzania. The Medical Birth Registry is within the hospital grounds at the Reproductive and Child Health Centre.

Participants: Singleton deliveries (N=42,319) with complete records from 2000 to 2015.

Primary outcome measures: Perinatal death, i.e., stillbirths and early neonatal deaths. These outcomes were only captured before mothers discharged from the hospital.

Results: The proportion of perinatal deaths was 3.7%. There were no statistically significant differences in the predictive performance of four machine learning models except for bagging, which had a significantly lower performance (AUC: 0.76, 95%CI 0.74, 0.79, p=0.006) compared to the logistic regression model (AUC: 0.78, 95%CI 0.76, 0.81). However, in the decision curve analysis, the machine learning models had a higher net benefit, i.e., the correct classification of perinatal deaths considering a trade-off between false-negatives and false-positives – over the logistic regression model across a range of threshold probability values.

Conclusions: In this cohort, there was no significant difference in the prediction of perinatal deaths between machine learning and logistic regression models, except for bagging. The machine learning models had a higher net benefit, as its predictive ability of perinatal death was considerably superior over the logistic regression model. The machine learning models, as demonstrated by our study, can be used to improve the prediction of perinatal deaths and triage for women at risk.

For peer review only - http://bmjopen.bmj.com/site/about/guidelines.xhtml

Strengths and limitations of this study

- The KCMC Medical Birth Registry cohort data provides a rich source of information for monitoring trends, inform clinical and administrative decisions, and enables complex modeling of the key predictors of perinatal deaths, among other adverse pregnancy outcomes.
- While standard regression models such as logistic regression are extensively applied in the literature to predict adverse pregnancy outcomes such as perinatal deaths, the application of machine learning models is limited.
- Machine learning algorithms may improve the prediction ability of perinatal deaths, and enable triage of women at high risk of experiencing adverse perinatal outcomes.
- The birth registry only captured deaths occurring in the KCMC hospital hence might have underestimated the proportion of perinatal deaths.

INTRODUCTION

Neonatal survival is at the heart of the sustainable development goals (SDG) agenda[1 2]. The Every Newborn Action Plan to end Preventable Deaths set a goal for all countries to reach the target of ten or less newborn deaths per 1000 live births and ten or less stillbirths per 1000 total births by the year 2035[3]. Furthermore, the United Nations set the target of reducing neonatal mortality to 12 deaths per 1000 live births or fewer by 2030[1]. Globally, neonatal deaths have declined by 51% from 5 million in 1990 to 2.5 million in 2017. But this decline has not been realized in low- and middle-income countries (LMICs), which carries the highest burden of neonatal deaths, with south Asia and sub-Saharan Africa accounted for 79% of the total burden of neonatal deaths in 2017[4]. Furthermore, the under-five mortality rate has decreased almost across the world, but the proportions of neonatal deaths remained high in this group[5 6]. Neonatal death has been reported to account for 47% of all under-five deaths in 2018, and it has increased from 40% in 1990, with sub-Saharan Africa bearing the highest burden[6]. Globally 2.5 million children died in the first month of life in 2018, with an approximately 7,000 newborn deaths every day[6]. Nearly three-quarters of these deaths occur during the first week, with about one million dying on the first day and close to one million dying within the next six days[6].

For peer review only - http://bmjopen.bmj.com/site/about/guidelines.xhtml

Globally, approximately 5.4 million perinatal deaths occur each year [2 7]. The majority (95%) of these deaths occur in sub-Saharan Africa and Southern Asia [8]. According to Tanzania Demographic and Health Survey, perinatal mortality rate has increased from 36 to 39 deaths per 1,000 live births between 2010-11 and 2015-16 survey rounds, respectively relative to under-five mortality[9]. Also, the perinatal mortality rate in Tanzania is the highest in East Africa[8].

Early identification of pregnant women at risk for adverse maternal and perinatal outcomes during the prenatal period and timely provision of high quality health care services have been reported to improve maternal and newborn survival[10]. Machine learning (hereafter denoted as 'ML') models are methodologies for developing algorithms that learn from existing data to make predictions on new data[10]. ML models have better predictive performance over the classical or conventional regression models [11], and they can better handle a significant number of potential predictors. However, there is conflicting evidence of the performance of these models. Previous investigators have demonstrated that, compared with the classical regression models, ML models have superior performance for early differentiation of sepsis and non-infectious systemic inflammatory response syndrome in critically ill children[12], prediction of neonatal and under-five mortality[13-17], and prediction of critical care and hospitalization outcomes[11 18 19]. In contrast, other studies have shown not predictive performance benefit of the ML models in prediction of clinical outcomes[10 20].

The first step in addressing high perinatal mortality is the accurate capture and classification of the causes of those deaths across all settings[21]. The World Health Organization (WHO) International Classification of Diseases (ICD-10) is a standardized tool used for the classification of deaths occurring during the perinatal period: ICD-PM[2 21 22]. ML models may be an essential tool in the assessment of risk factors for deaths during the perinatal period and triage pregnant women at high risk of experiencing adverse perinatal outcomes, especially in low resourced settings where the majority of perinatal deaths occur at home [23-26]. Capturing the chain of events that led to the perinatal mortality, from both the maternal and the perinatal side, informs the design and development of preventative and therapeutic measures[2].

Using data from the medical birth registry at Kilimanjaro Christian Medical Center (KCMC) referral hospital in northern Tanzania, we aimed to determine the key predictors of perinatal death using machine learning models. Previous studies using the same data[27-32] applied

For peer review only - http://bmjopen.bmj.com/site/about/guidelines.xhtml
For peer review only

standard regression models to assess risk factors for adverse perinatal outcomes. A major weakness of conventional regression analysis, as opposed to ML models, is that many covariates are excluded based on specific model assumptions. In contrast, ML techniques find the most predictive groupings of factors based on their frequency and strength of association, with no particular model assumptions [33]. In this study, we compared the predictive performance of the ML models with the conventional regression analysis, particularly logistic regression.

METHODS

Study design, setting, and population

We conducted a retrospective cohort study utilizing data from the KCMC referral hospital, situated in the Moshi Municipality of Kilimanjaro region, Northern Tanzania. The hospital receives deliveries from nearby communities and referral cases from other health care facilities inside the region and the neighboring regions[34]. The hospital has an average annual delivery rate of 4000 births[32 34 35]. The study population was women who delivered singleton babies. We included 42,319 deliveries with complete records between 2000 and 2015. We excluded records with missing values on the outcome (perinatal status) and the covariates as well as pregnancies with multiple gestations to avoid over-representation of high-risk pregnancies[32].

Data source

We used data from the KCMC referral hospital medical birth registry between the years 2000-2015, which were collected among mothers who delivered at the department of obstetrics and gynecology. More description of the KCMC medical birth registry is also available elsewhere[27 29 32 36 37]. Briefly, the KCMC medical birth registry is within hospital grounds at the Reproductive and Child Health Centre. The birth registry has been in operation since the year 2000, established to serve both clinical, administrative, and research purposes[36 37]. Trained midwives collected data using a standardized questionnaire (within 24 hours after delivery or later in case a mother had recovered from complications), after which data is entered into a computerized database located at the birth registry. Also, additional data were abstracted from the antenatal (ANC) cards and the hospital medical records of the mother[29].

For peer review only - http://bmjopen.bmj.com/site/about/guidelines.xhtml

A unique hospital identification number was assigned to each woman at first admission and used to trace her medical records at later admissions, and further to link records of successive births of the same woman[37]. Data captured information on the background characteristics of mother and father, mother's health before and during present pregnancy, information about delivery including complications, and child characteristics including their status (i.e., whether dead or alive).

Study variables

The main outcome variable in this study was perinatal death which was defined as the number of stillbirths (pregnancy loss that occurs after seven months of gestation) and early neonatal deaths (deaths of live births within the first seven days of life)[9 38]. The perinatal death was coded as binary, i.e., 'yes' if death occurred during the perinatal period and 'no' if otherwise. This outcome only captured deaths that occurred within the hospital before the discharge of mothers. There are no follow-up mechanisms for deaths that occur outside the health facility (KCMC hospital).

We included a total of 32 predictor variables for the machine learning models. Previous literature informed the selection of these variables[4 28 39-46], most of which are available in the birth registry. These included maternal and paternal background characteristics; age in years, area of residence (rural vs. urban), highest education level (none, primary, secondary and higher), marital status (single, married and widow/divorced), and occupation (unemployed, employed and others). Further, specific characteristics of the mother included referral status (whether referred for delivery or not), and the number of antenatal care visits (<4 and ≥4 visits).

We excluded maternal body mass index (BMI) and HIV status because they contributed to nearly 47% of all missing values in the dataset. Maternal health during pregnancy included; alcohol consumption, smoking, gestational diabetes, diabetes, hypertension, pre-eclampsia/eclampsia, bleeding (i.e., the woman observed blood from the vagina at any time during the pregnancy), anemia, malaria, and systemic infections/sepsis. Variables with information concerning delivery included; induction of labor (yes or no), mode of delivery (vaginal vs. caesarean section), presentation (breech vs. cephalic), complications during birth, particularly premature rupture of the membranes (PROM), postpartum hemorrhage (PPH),

For peer review only - http://bmjopen.bmj.com/site/about/guidelines.xhtml

placenta previa, and placenta abruption, all categorized as yes and no. Gestational age at birth was estimated based on the date of the last menstrual period and recorded in full weeks. Preterm was defined as babies born alive before 37 weeks of pregnancy are completed[47]. Child characteristics included sex (male or female), low birth weight defined as an infant birth weight of less than 2500g[28 48], and year of birth.

Statistical and computational analysis

Prior data were cleaned and then analyzed using Stata version 15.1[49]. Descriptive statistics were summarized using frequencies, and percentages for categorical variables. Chi-square test (χ^2) was used to compare proportion of perinatal death across participant characteristics. For the ML models, i.e., from feature selection, training, testing, and comparison of the predictive performance of the machines, we used R version 3.6.3[50]. The training dataset contained 70% of randomly selected samples used to develop six different ML models to predict perinatal death. These are artificial neural networks (ANN), random forests (RF), Naïve Bayes (NB), bagged trees, boosting, and the logistic regression (Lreg) model. We used the *caret* package to implement these models in R.

Briefly, ANN is a method constructed from three layers of connected nodes: input, hidden, and output[51]. The input where each input variable appears as a node; the hidden layer contains several nodes determined during the model tuning phase. In contrast, the output layer contains several nodes equal to the number of classes to be predicted[52]. Between these layers there are weighted links[10 51 52], the hidden layer receives a sum of the multiplication of the input variables with associated weights values plus the bias[51 52]. This value is entered into an activation function, such as a logistic or sigmoid function, to decide the class prediction. Outputs of the network are interpreted as class probabilities and sum to one[52]. We used *nnet* package to construct the ANN model.

RF is an extension of classification and regression trees (CART)[10 11 52 53]. RF performance is better compared to bagged trees because it decorrelates the trees[54], hence improves accuracy[53]. Several forests of decision trees are grown using a random bootstrapped training sample. Also, instead of using all the variables/features in each tree, a random sample of variables are selected and tested at each split in each tree[11 52 53]. The prediction is made for unobserved data by taking a

For peer review only - http://bmjopen.bmj.com/site/about/guidelines.xhtml

majority vote of the individual trees[52 53]. We used *randomForest* package to construct the RF model. NB is an effective classifier[51] due to its simplicity, exhibiting a surprisingly competitive predictive accuracy[55]. NB uses probability theory to find the most possible sample class in a classification problem. NB has two assumptions; (1) each attribute is conditionally independent of the other attributes given the class, and (2) all the attributes have an impact on the class[52 55]. We used *naivebayes* package to construct the NB model.

Logistic regression (Lreg) is a standard multivariate classification method. It arises from the desire to model the posterior probabilities via linear functions in covariates, such that besides predicting class labels, it provides a probabilistic interpretation of this labeling[54 56 57]. Lreg uses a sigmoid function instead of a linear function to map predictions to probabilities between 0 and 1[54]. We used *glm* method to construct the Lreg model. Bagging, or bootstrap aggregation and boosting are general techniques for improving prediction rules and accuracy of the resulting predictions, by reducing the associated variance of prediction[54 58]. Bagging divides the available data into many bootstrap samples and then train a separate model for each bootstrap, and then make a final prediction by averaging and voting for regression and classification, respectively[58]. Boosting, on the other hand, is a committee-based approach that uses a weighted average of prediction from various samples. The incorrectly predicted cases from a given step are given a higher weight during the next step. Thus, it is an iterative procedure, incorporating weights, as opposed to simple averaging of predictions[58]. We used *treebag* method and *gbm* package to construct the bagging and boosting models, respectively.

In the training set, parameter tuning and cross-validation aim to find a balance between building a model that can classify the training data effectively without overfitting to the random fluctuations[52]. For each ML model, we used 10-fold cross-validation as a resampling method, where the training set is divided equally into ten parts (folds). Therefore, every nine folds are used together for training the model and the remaining one-fold for testing. This training-testing process is repeated ten times. We used the Synthetic Minority Over-sampling Technique (SMOTE) method[59 60] to address the class imbalance in the outcome (i.e., the low proportion of perinatal deaths), by specifying the additional sampling to be "smote" on train control parameter specifications. SMOTE is a method that produces artificial minority samples by interpolating between existing minority samples and their nearest minority neighbors[59 60].

For peer review only - http://bmjopen.bmj.com/site/about/guidelines.xhtml

Using the testing set (30% of the remaining randomly selected sample), we computed the predictive performance of the six models (including logistic regression model) from the training set using the area under the receiver-operating-characteristics curve (AUC). We used the *ROCR* package for plotting ROC curves, obtaining the AUC values, and comparison of models using AUC values. We also used measures from the confusion matrix results (i.e., accuracy, sensitivity, specificity, positive and negative predictive values), and the net benefit through decision curve analysis[61 62] – which quantifies whether a machine provides a relevant improvement in the prediction. We used *epiR* package to obtain confidence intervals for the performance measures and *DCA* package (*http://www.decisioncurveanalysis.org*) for decision curve analysis. We further used *ggplot2* package to plot the decision curves. A good model will have a higher net benefit[61]. We used Delong's test to compare the receiver-operating-characteristics curve (ROC) between models, where, a p-value of <0.05 was considered statistically significant. The variable importance is a scaled measure with a maximum value of 100[18].

Patient and public involvement

There was no patient and public involvement.

RESULTS

Characteristics of study participants

The characteristics of the participants are shown in Table **1**. A total of 55,003 total deliveries were recorded at the KCMC medical birth registry from 2000 to 2015. Of these, we excluded 3,316 (6%) multiple gestations (to avoid over-representation of high-risk pregnancies)[32], 49 (0.1%) records missing maternal identification numbers (hence could not be linked to child records), 791 (1.4%) records with a mismatch between the date of birth and unknown sequence (i.e., singleton vs. multiple births). We further excluded a total of 8,528 (15.5%) observations with missing values in both the outcome (perinatal status) and covariates. We, therefore, analyzed data for a total of 42,319 singleton deliveries with complete records (Figure **1**).

Figure 1: Schematic diagram showing the number of singleton deliveries analyzed, KCMC medical birth registry data, 2000-2015.

For peer review only - http://bmjopen.bmj.com/site/about/guidelines.xhtml

The overall proportion of perinatal death among 42,319 singleton deliveries in this study was 3.7%. The proportion of perinatal deaths among mothers aged 20-34, 35-39, and 40+ years was 3.5%, 4.7%, and 5.8%, respectively. Mothers with no education (5.6%) and those with primary education level (4.4%), who resided in rural areas (4.8%), had less than four antenatal care (ANC) visits (5.9%), and those referred for delivery (7.8%) had a higher proportion of perinatal death. Among fathers, a higher proportion of perinatal death is among those aged 35+ years (4.1%), with no (9.6%) or with primary education level (4.6%) as well as those who were unemployed (5.7%), Table **1**.

Furthermore, the most common obstetric care and complications in this birth cohort included induction of labor (22.7%), malaria (13.2%), preterm birth (10.8%), and LBW (10.2%). About 4% of mothers in this cohort experienced pre-eclampsia/ eclampsia during pregnancy. Less than half of all children were females. The proportion of perinatal death among women who experienced induction of labor, with malaria, delivered preterm, delivered LBW baby, and experienced pre-eclampsia/ eclampsia during pregnancy was 4.8%, 3.8%, 14.2%, 17.6%, and 12.5%, respectively. The proportion of perinatal death is almost similar among males (3.8%) compared to females (3.6%) children in this cohort (Table **1**).

For peer review only - http://bmjopen.bmj.com/site/about/guidelines.xhtml
For peer review only

Table 1: Characteristics of study participants (N=42,319)

Characteristics	Total	Perinatal death	P-value*	Characteristics	Total	Perinatal death	P-value*
Maternal	**n (%)**	**n (%)**		***Obstetrics***	**n (%)**	**n (%)**	
**Age (years)**			<0.001	**Gestational diabetes**			0.89
15-19	3470 (8.2)	99 (2.9)		No	42288 (99.9)	1560 (3.7)	
20-34	32675 (77.2)	1158 (3.5)		Yes	31 (0.1)	1 (3.2)	
35-39	4984 (11.8)	235 (4.7)		**Diabetes**			0.002
40+	1190 (2.8)	69 (5.8)		No	42240 (99.8)	1553 (3.7)	
**Education level**			<0.001	Yes	79 (0.2)	8 (10.1)	
None	567 (1.3)	32 (5.6)		**Hypertension**			<0.001
Primary	23010 (54.4)	1019 (4.4)		No	42241 (99.8)	1550 (3.7)	
Secondary	5275 (12.5)	159 (3.0)		Yes	78 (0.2)	11 (14.1)	
Higher	13467 (31.8)	351 (2.6)		**Bleeding**			<0.001
**Occupation**			0.37	No	41897 (99.0)	1528 (3.6)	
Unemployed	9316 (22.0)	365 (3.9)		Yes	422 (1.0)	33 (7.8)	
Employed	30061 (71.0)	1085 (3.6)		**Anemia**			0.004
Others	2942 (7.0)	111 (3.8)		No	41661 (98.4)	1523 (3.7)	
**Marital status**			0.89	Yes	658 (1.6)	38 (5.8)	
Single	4954 (11.7)	186 (3.8)		**Malaria**			0.79
Married	37300 (88.1)	1372 (3.7)		No	36746 (86.8)	1352 (3.7)	
Widowed/Divorced	65 (0.2)	3 (4.6)		Yes	5573 (13.2)	209 (3.8)	
**Area of residence**			<0.001	**Sepsis/ infections**			0.43
Urban	25056 (59.2)	725 (2.9)		No	41588 (98.3)	1538 (3.7)	
Rural	17263 (40.8)	836 (4.8)		Yes	731 (1.7)	23 (3.1)	
**Alcohol consumption during pregnancy**			0.001	***Complications***			
No	30759 (72.7)	1191 (3.9)		**Pre-eclampsia/ eclampsia**			<0.001
Yes	11560 (27.3)	370 (3.2)		No	40668 (96.1)	1355 (3.3)	
**Smoking during pregnancy**			0.97	Yes	1651 (3.9)	206 (12.5)	
Yes	53 (0.1)	2 (3.8)		**Induction of labor**			<0.001
No	42266 (99.9)	1559 (3.7)		No	32732 (77.3)	1105 (3.4)	
**Number of ANC visits**			<0.001	Yes	9587 (22.7)	456 (4.8)	
≥4	28742 (67.9)	760 (2.6)		**PROM**			0.006
<4	13577 (32.1)	801 (5.9)		No	41416 (97.9)	1543 (3.7)	

For peer review only

**Referred for delivery**			<0.001
No	32762 (77.4)	819 (2.5)	
Yes	9557 (22.6)	742 (7.8)	
Paternal characteristics			
**Age (years)**			0.001
<25	3938 (9.3)	122 (3.1)	
25-29	10593 (25.0)	346 (3.3)	
30-34	12303 (29.1)	457 (3.7)	
35+	15485 (36.6)	636 (4.1)	
**Education level**			<0.001
None	281 (0.7)	27 (9.6)	
Primary	18987 (44.9)	868 (4.6)	
Secondary	4565 (10.8)	154 (3.4)	
Higher	18486 (43.7)	512 (2.8)	
**Occupation**			<0.001
Unemployed	5710 (13.5)	323 (5.7)	
Employed	36102 (85.3)	1218 (3.4)	
Others	507 (1.2)	20 (3.9)	
Yes	903 (2.1)	18 (2.0)	
**PPH**			<0.001
No	42091 (99.5)	1516 (3.6)	
Yes	228 (0.5)	45 (19.7)	
**3-4-degree tear**			0.49
No	42305 (99.9)	1560 (3.7)	
Yes	14 (0.1)	1 (7.1)	
**Abruption placenta**			<0.001
No	42193 (99.7)	1490 (3.5)	
Yes	126 (0.3)	71 (56.3)	
**Placenta previa**			0.04
No	42245 (99.8)	1555 (3.7)	
Yes	74 (0.2)	6 (8.1)	
**Presentation**			<0.001
Cephalic	41833 (98.9)	1459 (3.5)	
Breach/ Transverse	486 (1.1)	102 (21.0)	
**Gestational age at birth**			<0.001
Term birth (>=37 weeks)	37764 (89.2)	914 (2.4)	
Preterm birth (<37 weeks)	4555 (10.8)	647 (14.2)	
**Birth weight**			<0.001
NBW	37991 (89.8)	801 (2.1)	
LBW	4328 (10.2)	760 (17.6)	
**Child's sex**			0.42
Female	20430 (48.3)	738 (3.6)	
Male	21889 (51.7)	823 (3.8)	
**Total**	**42319**	**1561 (3.7%)**	

[*]P-value based on the Chi-square test.

For peer review only - http://bmjopen.bmj.com/site/about/guidelines.xhtml
For peer review only

The trends in the proportion of perinatal deaths that occurred at KCMC between the years 2000-2015 are shown in Figure **2**. Overall, the proportion of perinatal deaths has slightly declined over the years by 6% (95%CI, 0.3%, 12.3%). Though this decline was not statistically significant (p=0.06).

Figure 2: Trends of perinatal death, KCMC Medical Birth Registry data, 2000-2015.

Variable importance

We used the random forest algorithm for variable selection. This model selected a total of 20 important predictors based on its threshold measure of importance out of the 32 variables. We used these 20 variables in all the subsequent analysis (Figure **3**).

Figure 3: Variable importance of predictors for perinatal death in the random forest model scaled to have a maximum value of 100.

Predicting perinatal deaths

The discriminatory abilities of all models for the prediction of perinatal death are in Figure **4a** and Table **2**. There were no significant differences (p>0.05) in the area under the receiver-operating characteristics curve (AUC) between logistic regression with random forest (RF), artificial neural networks (ANN), boosting, and naïve Bayes (NB). However, bagging had significantly lower predictive performance (AUC: 0.76, 95%CI 0.74, 0.79, p=0.006) compared to the logistic regression model (AUC: 0.78, 95%CI 0.76, 0.81). Furthermore, the ANN model (Sensitivity: 0.60, 95% CI 0.55, 0.64) and NB model (Sensitivity: 0.57, 95% CI 0.52, 0.62) had slightly higher sensitivity compared to logistic regression (Sensitivity: 0.56, 95%CI 0.51, 0.60) while boosting (Specificity: 0.89 95%CI 0.88, 0.89) and RF (Specificity: 0.88, 95%CI 0.88, 0.89) had slightly higher specificity compared to logistic regression (Specificity: 0.87, 95%CI 0.86, 0.88). Due to the low prevalence of perinatal deaths (3.7%), all models had high negative predictive values (NPV: 0.98, 95%CI 0.98, 0.98).

With regard to the number of actual and predicted outcomes (Table **3**), all models correctly predicted perinatal deaths by more than half of 468 deaths in the testing set. The numbers of correct classification were higher in the ANN 280 (59.8%) and NB 267 (57.1%),

For peer review only - http://bmjopen.bmj.com/site/about/guidelines.xhtml

followed by the logistic regression model 261 (55.8%) and Bagging 260 (55.6%). The decision curve analysis (Figure 4b) demonstrated that the net benefit of the RF model surpassed that of other machine learning models, including logistic regression for all threshold values, indicating that the RF model is more superior in predicting the risk of perinatal deaths in this cohort. The accuracy of the RF model was 0.87, 95%CI (0.86, 0.87), compared to 0.87, 95%CI (0.87, 0.88) for boosting and 0.86, 95%CI (0.85, 0.86) for the logistic regression model (Table **2**). Furthermore, other ML models (except for boosting) demonstrated high net benefit over a range of threshold probability values relative to that of the logistic regression model. Also, the random forest model had a superior net benefit over all models (Figure **4b**).

For peer review only

For peer review only - http://bmjopen.bmj.com/site/about/guidelines.xhtml

Table 2: Prediction performance of the reference and machine learning models in the test set

Model	Logistic Regression	Artificial Neural Network	Random Forests	Naïve Bayes	Bagging	Boosting
ACC	0.86 (0.85, 0.86)	0.83 (0.82, 0.83)	0.87 (0.86, 0.87)	0.84 (0.83, 0.85)	0.82 (0.81, 0.83)	0.87 (0.87, 0.88)
AUC	0.78 (0.76, 0.81)	0.78 (0.76, 0.80)	0.79 (0.76, 0.81)	0.79 (0.76, 0.81)	0.76 (0.74, 0.79)	0.79 (0.76, 0.81)
P-value*	Reference	0.59	0.37	0.65	0.006	0.20
Sensitivity	0.56 (0.51, 0.60)	0.60 (0.55, 0.64)	0.54 (0.49, 0.58)	0.57 (0.52, 0.62)	0.55 (0.50, 0.59)	0.54 (0.49, 0.58)
Specificity	0.87 (0.86, 0.88)	0.84 (0.83, 0.84)	0.88 (0.88, 0.89)	0.85 (0.84, 0.86)	0.83 (0.82, 0.84)	0.89 (0.88, 0.89)
PPV	0.14 (0.12, 0.16)	0.12 (0.11, 0.14)	0.15 (0.13, 0.17)	0.13 (0.11, 0.14)	0.11 (0.10, 0.12)	0.15 (0.14, 0.17)
NPV	0.98 (0.98, 0.98)	0.98 (0.98, 0.98)	0.98 (0.98, 0.98)	0.98 (0.98, 0.98)	0.98 (0.98, 0.98)	0.98 (0.98, 0.98)

Abbreviations: ACC, Accuracy; AUC, Area Under the Curve; PPV, Positive Predictive Value; NPV, Negative Predictive Value
*We calculated p-values to compare the area under the receiver-operating-characteristics curve (AUC) of logistic with each machine learning model

Table 3: The number of actual and predicted outcomes of prediction models in the test set

Prediction Model	Classification	Perinatal status	
		Alive	Died
	Actual number of events	12227	468
Logistic Regression	Correctly predicted outcome	10627	261
	Incorrectly predicted outcome	1600	207
Artificial Neural Network	Correctly predicted outcome	10225	280
	Incorrectly predicted outcome	2002	188
Random Forests	Correctly predicted outcome	10774	251
	Incorrectly predicted outcome	1453	217
Naïve Bayes	Correctly predicted outcome	10386	267
	Incorrectly predicted outcome	1841	201
Bagging	Correctly predicted outcome	10175	260
	Incorrectly predicted outcome	2052	208
Boosting	Correctly predicted outcome	10852	252
	Incorrectly predicted outcome	1375	216

For peer review only - http://bmjopen.bmj.com/site/about/guidelines.xhtml
For peer review only

Figure 4: Prediction ability of perinatal deaths comparing different machine learning models in the test set: [a] Receiver-operating-characteristics (ROC) curves. The corresponding values of the area under the receiver-operating-characteristics curve (AUC) for each model are in Table 2. [b] Decision curve analysis. The net benefit of the machine learning models (except for boosting) is larger over a range of threshold probability values compared to that of the logistic regression model.

DISCUSSION

In this study, the perinatal death was predicted using five ML models (Artificial Neural Networks, Random Forests, Naïve Bayes, Bagging, and Boosting). There were no differences in the predictive performance between ML models except for bagging, which had a lower predictive performance. The artificial neural networks and naïve Bayes had higher sensitivity compared to the logistic regression, and other ML models. Specificity for all models was high, mainly due to the low prevalence of perinatal deaths in this cohort. Additionally, results from the decision curve analysis revealed that the ML models (except for boosting) had a higher net benefit over a range of threshold probability values compared to the logistic regression model, indicating high accuracy. The random forest model demonstrated a superior net benefit over other models.

In the present study, maternal characteristics before and during pregnancy, as well as pregnancy history, were used to identify pregnancies at high risk of experiencing adverse perinatal outcomes that might need close clinical follow-ups. On top of clinicians' judgment, previous investigators applied standard regression models in prediction of risk for adverse perinatal outcomes, particularly perinatal death[30 40-46 63-68]. We found no differences in the predictive performance of the ML models, except for bagging, which had lower predictive capacity. The sensitivity of the machine learning models was also almost comparable to that of logistic regression, which indicates that both models correctly classified perinatal deaths. Our finding is consistent with a recent systematic review that showed no performance benefit of ML models over logistic regression for the prediction of clinical outcomes[20]. The possible explanation for lack of differences in the performance between the compared models could be attributed to the low proportion of outcome and exposures in this study, as well as data quality and recording challenges.

For peer review only - http://bmjopen.bmj.com/site/about/guidelines.xhtml

In contrast, some previous investigators have demonstrated that ML models offers better predictions of clinical or adverse pregnancy outcomes compared to classical regression models [14-17]. The application of ML models may improve the classification of adverse events occurring during the perinatal period and, therefore, assist in triaging and provision of close clinical follow-up for women at high-risk. Other studies also provide evidence of improved prediction of under-five and neonatal mortality[13-16] using ML models. The utility of these models may, therefore, improve the prediction of adverse pregnancy outcomes as opposed to standard regression models.

In this study, the decision curve analysis that accounts for the impact of false-negative and false-positive misclassification errors showed superior predictive performance of the ML approaches over the logistic regression model. This demonstrates a higher net benefit for the prediction of perinatal deaths. The higher net benefit in the prediction ability of the ML approaches has also been documented elsewhere[11 18]. This is because ML approaches can incorporate the high order nonlinear interactions between predictors, which cannot be addressed by traditional modeling approaches, including the logistic regression model. Furthermore, the use of cross-validation is also known to reduce potential overfitting in ML models. It is important to note that ML approaches are, to a large extent, non-parametric as opposed to the logistic regression model that relies on strong distributional assumptions.

The strength of this study is that it is the first to apply modern ML approaches to predict perinatal deaths, particularly in Tanzania, compared with the classical logistic regression model. Our study demonstrated that ML models might be used to improve the prediction of perinatal deaths and triage of women at risk. We also used SMOTE balancing technique to avoid the bias of the model toward skewed data, hence improving prediction accuracy of the ML algorithm[15]. Our study also had some limitations that are worth considering when interpreting the results. Firstly, we excluded observations with missing values in both the outcome and exposures from the analysis, a problem inherent in cohort studies, including birth registries, which may lead to under-estimation of the proportion of perinatal death. Two excluded variables (maternal BMI and HIV status) have been associated with perinatal and under-five deaths[4 6 39]; hence their exclusion might increase the risk of residual confounding bias. The effect of exclusion of these two variables and missing values to predict perinatal deaths remains unquantified.

For peer review only - http://bmjopen.bmj.com/site/about/guidelines.xhtml

Secondly, selection bias/ or referral bias is a common problem in hospital-based studies, which affects the generalization of findings to the general population. This might also be the case in the present study. However, our findings might reflect a similar setting in Tanzania and probably in other sub-Saharan African countries. Thirdly, the KCMC Medical Birth Registry cohort only captures perinatal deaths occurring in the health facility (KCMC hospital), which may underestimate the observed perinatal deaths. Currently, the hospital has no mechanisms to follow-up the birth outcomes from deliveries that occur at home and post-discharge outcomes of the babies after mothers are discharged from the hospital within the first week, especially within the KCMC hospital catchment area. Future extensions include ways of handling missing values before applying the machine learning algorithms.

CONCLUSION

The ML models (except for bagging) performed equally with the logistic regression model to predict perinatal deaths using maternal, paternal, and obstetric factors in this cohort. The ML models, however, have a higher net benefit, demonstrating superiority in the prediction of perinatal death. Furthermore, the random forest model also demonstrated superior performance over other ML models. These models are a useful and alternative strategy over standard logistic regression model to predict perinatal deaths, considering the richness of the medical birth registries. Moreover, the ML models are capable of handling many predictors at the same time, which is crucial in capturing a multiple risk factors for adverse perinatal outcomes such as perinatal deaths. The application of ML models may, therefore, increase the prediction ability of adverse perinatal outcomes and thereby helping in triage women most at risk.

Acknowledgements

We would like to acknowledge the midwives who participated in data collection and all women and children whose information enabled the availability of data used in this study. The authors also thank the staff at the Birth Registry for capturing these data in the electronic system. We also appreciate the Centre for International Health at the University of Bergen in Norway and the Department of Obstetrics and Gynecology of the KCMC hospital in Tanzania for establishing the KCMC medical birth registry, which facilitated the availability of data to conduct this study.

For peer review only - http://bmjopen.bmj.com/site/about/guidelines.xhtml
For peer review only

Author contributions

All authors made a substantial contribution to this study. IBM, MJM, JO and HGM designed the study. IBM and MM analyzed the data. MJM, JO and HGM commented on the manuscript. IBM drafted the manuscript and had primary responsibility for the final content. All authors reviewed the drafts of this manuscript and approved the final version for submission.

Funding

This work was supported through the DELTAS Africa Initiative Grant No. 107754/Z/15/ZDELTAS Africa SSACAB. The DELTAS Africa Initiative is an independent funding scheme of the African Academy of Sciences (AAS)'s Alliance for Accelerating Excellence in Science in Africa (AESA) and supported by the New Partnership for Africa's Development Planning and Coordinating Agency (NEPAD Agency) with funding from the Wellcome Trust (Grant No. 107754/Z/15/Z) and the UK government. The views expressed in this publication are those of the author(s) and not necessarily those of AAS, NEPAD Agency, Wellcome Trust or the UK government. The Norwegian Council for Higher Education's Program for Development Research (NUFU) funded the establishment of the birth registry at the Kilimanjaro Christian Medical Center (KCMC). The funders had no role in study design, data collection, and analysis, decision to publish, or preparation of the manuscript.

Competing interests

None declared.

Patient consent for publication

Not required.

Ethics approval

This study was approved by the Kilimanjaro Christian Medical College Research Ethics and Review Committee (KCMU-CRERC) with approval number 2424. Before data collection, mothers provided oral informed consent after receiving information about the purpose of the study, questionnaire, and its contents as well as the intention to gather new knowledge, which will, in turn, benefit mothers and children in the future. Mothers were informed that participation

For peer review only

For peer review only - http://bmjopen.bmj.com/site/about/guidelines.xhtml

was voluntary and had no implications on the care they would receive. Following consent, mothers were free to refuse to reply to single questions. For privacy and confidentiality, unique identification numbers were used to identify and then link mothers with child records. There was no person-identifiable information in any electronic database, and instead, unique identification numbers were used. Necessary measures were taken by midwives to ensure privacy during the interview process.

Data availability statement

The KCMC medical birth registry data contains potentially identifying and sensitive patient information. This has also been stipulated by the local Institutional Review Board of KCMC hospital and the National Ethics Committee in Norway when establishing this birth registry. Permission to use the data in this study was made through the Kilimanjaro Christian Medical University College Research and Ethics Review Committee, and received an approval number 2424. The authors do not have the legal right to share the data publicly. All data requests can be sent to the Executive Director of the KCMC referral hospital, P. O. Box 3010, Moshi, Tanzania, Email: kcmcadmin@kcmc.ac.tz or through the corresponding author.

ORCID iDs

Innocent B. Mboya: https://orcid.org/0000-0001-9861-5879

Michael J. Mahande: http://orcid.org/0000-0002-7750-7657

Mohanad Mohammed: https://orcid.org/0000-0001-5336-0286

Henry G. Mwambi: https://orcid.org/0000-0001-9654-400X

REFERENCES

1. United Nations Development Program. Sustainable development goals: United Nations; 2019 [Available from: https://www.undp.org/content/undp/en/home/sustainable-development-goals/goal-3-good-health-and-well-being.html#targets accessed August 14 2019.
2. World Health Organization. The WHO application of ICD-10 to deaths during the perinatal period: ICD-PM. Geneva: World Health Organization, 2016.
3. World Health Organization. Every newborn: an action plan to end preventable deaths. 2014
4. Hug L, Alexander M, You D, et al. National, regional, and global levels and trends in neonatal mortality between 1990 and 2017, with scenario-based projections to 2030: a systematic analysis. *The Lancet Global Health* 2019;7(6):e710-e20.
5. Burstein R, Henry NJ, Collison ML, et al. Mapping 123 million neonatal, infant and child deaths between 2000 and 2017. *Nature* 2019;574(7778):353-58.

For peer review only - http://bmjopen.bmj.com/site/about/guidelines.xhtml

6. UNICEF. Levels & Trends in Child Mortality: Report 2019. Estimates Developed by the UN Inter-agency Group for Child Mortality Estimation: United Nations Children's Fund, 2019.

7. World Health Organization. Maternal and perinatal health: World Health Organization; 2019 [Available from: http://www.who.int/maternal_child_adolescent/topics/maternal/maternal_perinatal/en/ accessed August 14 2019.

8. Akombi BJ, Renzaho AM. Perinatal Mortality in Sub-Saharan Africa: A Meta-Analysis of Demographic and Health Surveys. *Annals of global health* 2019;85(1)

9. MoHCDGEC, MoH, NBS, et al. Tanzania Demographic and Health Survey and Malaria Indicator Survey (TDHS-MIS) 2015-16. Dar es Salaam, Tanzania and Rockville, Maryland, USA, 2016.

10. Kuhle S, Maguire B, Zhang H, et al. Comparison of logistic regression with machine learning methods for the prediction of fetal growth abnormalities: a retrospective cohort study. *BMC pregnancy and childbirth* 2018;18(1):1-9.

11. Raita Y, Goto T, Faridi MK, et al. Emergency department triage prediction of clinical outcomes using machine learning models. *Crit Care* 2019;23(1):64. doi: 10.1186/s13054-019-2351-7 [published Online First: 2019/02/24]

12. Lamping F, Jack T, Rübsamen N, et al. Development and validation of a diagnostic model for early differentiation of sepsis and non-infectious SIRS in critically ill children-a data-driven approach using machine-learning algorithms. *BMC pediatrics* 2018;18(1):112.

13. Nasejje JB, Mwambi H. Application of random survival forests in understanding the determinants of under-five child mortality in Uganda in the presence of covariates that satisfy the proportional and non-proportional hazards assumption. *BMC Res Notes* 2017;10(1):459. doi: 10.1186/s13104-017-2775-6 [published Online First: 2017/09/09]

14. Houweling TA, van Klaveren D, Das S, et al. A prediction model for neonatal mortality in low-and middle-income countries: an analysis of data from population surveillance sites in India, Nepal and Bangladesh. *International journal of epidemiology* 2019;48(1):186-98.

15. Hoodbhoy Z, Noman M, Shafique A, et al. Use of machine learning algorithms for prediction of fetal risk using cardiotocographic data. *International Journal of Applied and Basic Medical Research* 2019;9(4):226.

16. Muktan D, Singh RR, Bhatta NK, et al. Neonatal mortality risk assessment using SNAPPE-II score in a neonatal intensive care unit. *BMC Pediatr* 2019;19(1):279. doi: 10.1186/s12887-019-1660-y [published Online First: 2019/08/15]

17. Lee SM, Lee MH, Chang YS, et al. The Clinical Risk Index for Babies II for Prediction of Time-Dependent Mortality and Short-Term Morbidities in Very Low Birth Weight Infants. *Neonatology* 2019;116(3):244-51. doi: 10.1159/000500270 [published Online First: 2019/07/16]

18. Goto T, Camargo CA, Jr., Faridi MK, et al. Machine Learning-Based Prediction of Clinical Outcomes for Children During Emergency Department Triage. *JAMA Netw Open* 2019;2(1):e186937. doi: 10.1001/jamanetworkopen.2018.6937 [published Online First: 2019/01/16]

19. Vellido A, Ribas V, Morales C, et al. Machine learning in critical care: state-of-the-art and a sepsis case study. *Biomedical engineering online* 2018;17(1):135.

20. Christodoulou E, Ma J, Collins GS, et al. A systematic review shows no performance benefit of machine learning over logistic regression for clinical prediction models. *J Clin Epidemiol* 2019;110:12-22. doi: 10.1016/j.jclinepi.2019.02.004 [published Online First: 2019/02/15]

For peer review only

For peer review only - http://bmjopen.bmj.com/site/about/guidelines.xhtml

21. Allanson E, Tuncalp O, Gardosi J, et al. Classifying the causes of perinatal death. *Bull World Health Organ* 2016;94(2):79-79A. doi: 10.2471/BLT.15.168047 [published Online First: 2016/02/26]
22. Wojcieszek AM, Reinebrant HE, Leisher SH, et al. Characteristics of a global classification system for perinatal deaths: a Delphi consensus study. *BMC pregnancy and childbirth* 2016;16(1):223.
23. Pitt C, Tawiah T, Soremekun S, et al. Cost and cost-effectiveness of newborn home visits: findings from the Newhints cluster-randomised controlled trial in rural Ghana. *The Lancet Global Health* 2016;4(1):e45-e56.
24. World Health Organization. Newborns: reducing mortality 2020 [cited 2020 02 May]. Available from: https://www.who.int/news-room/fact-sheets/detail/newborns-reducing-mortality.
25. Khan FA, Mullany LC, Wu LF, et al. Predictors of neonatal mortality: development and validation of prognostic models using prospective data from rural Bangladesh. *BMJ Glob Health* 2020;5(1):e001983. doi: 10.1136/bmjgh-2019-001983 [published Online First: 2020/03/07]
26. Chaibva BV, Olorunju S, Nyadundu S, et al. Adverse pregnancy outcomes,'stillbirths and early neonatal deaths' in Mutare district, Zimbabwe (2014): a descriptive study. *BMC pregnancy and childbirth* 2019;19(1):86.
27. Mmbaga BT, Lie RT, Kibiki GS, et al. Transfer of newborns to neonatal care unit: a registry based study in Northern Tanzania. *BMC Pregnancy and Childbirth* 2011;11(1):68. doi: 10.1186/1471-2393-11-68
28. Mitao M, Philemon R, Obure J, et al. Risk factors and adverse perinatal outcome associated with low birth weight in Northern Tanzania: a registry-based retrospective cohort study. *Asian Pacific Journal of Reproduction* 2016;5(1):75-79. doi: 10.1016/j.apjr.2015.12.014
29. Mahande MJ, Daltveit AK, Obure J, et al. Recurrence of preterm birth and perinatal mortality in northern Tanzania: registry-based cohort study. *Trop Med Int Health* 2013;18(8):962-7. doi: 10.1111/tmi.12111 [published Online First: 2013/04/16]
30. Mahande MJ, Daltveit AK, Mmbaga BT, et al. Recurrence of perinatal death in Northern Tanzania: a registry based cohort study. *BMC pregnancy and childbirth* 2013;13(1):166.
31. Isaksen AB, Østbye T, Mmbaga BT, et al. Alcohol consumption among pregnant women in Northern Tanzania 2000–2010: a registry-based study. *BMC Pregnancy and Childbirth* 2015;15(1):205. doi: 10.1186/s12884-015-0630-0
32. Chuwa FS, Mwanamsangu AH, Brown BG, et al. Maternal and fetal risk factors for stillbirth in Northern Tanzania: A registry-based retrospective cohort study. *PloS one* 2017;12(8):e0182250.
33. Hamilton EF, Dyachenko A, Ciampi A, et al. Estimating risk of severe neonatal morbidity in preterm births under 32 weeks of gestation. *The Journal of Maternal-Fetal & Neonatal Medicine* 2020;33(1):73-80.
34. Mahande AM, Mahande MJ. Prevalence of parasitic infections and associations with pregnancy complications and outcomes in northern Tanzania: a registry-based cross-sectional study. *BMC Infect Dis* 2016;16:78. doi: 10.1186/s12879-016-1413-6 [published Online First: 2016/02/15]
35. Temu TB, Masenga G, Obure J, et al. Maternal and obstetric risk factors associated with preterm delivery at a referral hospital in northern-eastern Tanzania. *Asian Pacific Journal of Reproduction* 2016;5(5):365-70. doi: 10.1016/j.apjr.2016.07.009

For peer review only

For peer review only - http://bmjopen.bmj.com/site/about/guidelines.xhtml

36. Bergsjo P, Mlay J, Lie RT, et al. A medical birth registry at Kilimanjaro Christian Medical Centre. *East Afr J Public Health* 2007;4(1):1-4.
37. Mahande MJ. Recurrence of Perinatal Death, Preterm Birth and Preeclampsia in Northern Tanzania: A Registry based Study. University of Bergen, 2015.
38. World Health Organization. Maternal and perinatal health: World Health Organization; 2020 [Available from: http://www.who.int/maternal_child_adolescent/topics/maternal/maternal_perinatal/en/ accessed March 02 2020.
39. Nijkamp JW, Sebire NJ, Bouman K, et al. Perinatal death investigations: What is current practice? *Semin Fetal Neonatal Med* 2017;22(3):167-75. doi: 10.1016/j.siny.2017.02.005 [published Online First: 2017/03/23]
40. Getiye Y, Fantahun M. Factors associated with perinatal mortality among public health deliveries in Addis Ababa, Ethiopia, an unmatched case control study. *BMC Pregnancy Childbirth* 2017;17(1):245. doi: 10.1186/s12884-017-1420-7 [published Online First: 2017/07/28]
41. Allanson ER, Muller M, Pattinson RC. Causes of perinatal mortality and associated maternal complications in a South African province: challenges in predicting poor outcomes. *BMC Pregnancy Childbirth* 2015;15:37. doi: 10.1186/s12884-015-0472-9 [published Online First: 2015/04/17]
42. Vogel JP, Souza JP, Mori R, et al. Maternal complications and perinatal mortality: findings of the World Health Organization Multicountry Survey on Maternal and Newborn Health. *BJOG: An International Journal of Obstetrics & Gynaecology* 2014;121(Suppl. 1):76-88. doi: 10.1111/1471-0528.12633
43. Unterscheider J, O'Donoghue K, Daly S, et al. Fetal growth restriction and the risk of perinatal mortality–case studies from the multicentre PORTO study. *BMC pregnancy and childbirth* 2014;14(1):63.
44. Mpembeni R, Jonathan R, Mughamba J. Perinatal Mortality and Associated Factors Among Deliveries in Three Municipal Hospitals of Dar Es Salaam, Tanzania. *Journal of Pediatrics & Neonatal Care* 2014;1(4) doi: 10.15406/jpnc.2014.01.00022
45. Ouyang F, Zhang J, Betran AP, et al. Recurrence of adverse perinatal outcomes in developing countries. *Bull World Health Organ* 2013;91(5):357-67. doi: 10.2471/BLT.12.111021 [published Online First: 2013/05/17]
46. Mutsaerts MA, Groen H, Buiter-Van der Meer A, et al. Effects of paternal and maternal lifestyle factors on pregnancy complications and perinatal outcome. A population-based birth-cohort study: the GECKO Drenthe cohort. *Hum Reprod* 2014;29(4):824-34. doi: 10.1093/humrep/deu006 [published Online First: 2014/02/11]
47. WHO. Preterm birth: World Health Organization; 2020 [Available from: http://www.who.int/news-room/fact-sheets/detail/preterm-birth accessed 19 February 2020.
48. World Health Organization. WHA Global nutrition targets 2025: low birth weight policy brief. Geneva: World Health Organization, 2014.
49. StataCorp. Stata Statistical Software: Release 15. College Station, TX: StataCorp LLC, 2017.
50. R Core Team. R: A Language and Environment for Statistical Computing. Vienna, Austria: R Foundation for Statistical Computing, 2020.
51. Dwivedi AK. Artificial neural network model for effective cancer classification using microarray gene expression data. *Neural Computing and Applications* 2016;29(12):1545-54. doi: 10.1007/s00521-016-2701-1

For peer review only

52. Stephens D, Diesing M. A comparison of supervised classification methods for the prediction of substrate type using multibeam acoustic and legacy grain-size data. *PLoS One* 2014;9(4):e93950. doi: 10.1371/journal.pone.0093950 [published Online First: 2014/04/05]
53. Breiman L. Random forests. *Machine learning* 2001;45(1):5-32.
54. Hastie T, Tibshirani R, Friedman J. The Elements of Statistical Learning: Data Mining, Inference, and Prediction. Second ed: Springer 2009.
55. Bayesian networks classifiers for gene-expression data. 2011 11th International Conference on Intelligent Systems Design and Applications; 2011. IEEE.
56. Musa AB. Gene expression data classification with kernel independent component analysis. *Research Journal of Mathematical and Statistical Sciences* 2014;2320:6047.
57. Dreiseitl S, Ohno-Machado L. Logistic regression and artificial neural network classification models: a methodology review. *Journal of biomedical informatics* 2002;35(5-6):352-59.
58. Sutton CD. Classification and Regression Trees, Bagging, and Boosting. Data Mining and Data Visualization2005:303-29.
59. Johnson JM, Khoshgoftaar TM. Survey on deep learning with class imbalance. *Journal of Big Data* 2019;6(1):27.
60. Chawla NV. Data mining for imbalanced datasets: An overview. Data mining and knowledge discovery handbook: Springer 2009:875-86.
61. Vickers AJ, Elkin EB. Decision curve analysis: a novel method for evaluating prediction models. *Medical Decision Making* 2006;26(6):565-74.
62. Zhang Z, Rousson V, Lee W-C, et al. Decision curve analysis: a technical note. *Annals of translational medicine* 2018;6(15)
63. Carvalho CA, da Silva AA, Victora C, et al. Changes in Infant and Neonatal Mortality and Associated Factors in Eight Cohorts from Three Brazilian Cities. *Scientific Reports* 2020;10(1):1-8.
64. Habimana-Kabano I, Broekhuis A, Hooimeijer P. The Effects of Interpregnancy Intervals and Previous Pregnancy Outcome on Fetal Loss in Rwanda (1996–2010). *International journal of reproductive medicine* 2015;2015
65. Flores Navarro-Perez C, Gonzalez-Jimenez E, Schmidt-RioValle J, et al. Sociodemographic Factors and Adequacy of Prenatal Care Associated Perinatal Mortality in Colombian Pregnant Women. *Nutr Hosp* 2015;32(3):1091-8. doi: 10.3305/nh.2015.32.3.9179 [published Online First: 2015/09/01]
66. Ali AA, Elgessim ME, Taha E, et al. Factors associated with perinatal mortality in Kassala, Eastern Sudan: a community-based study 2010-2011. *J Trop Pediatr* 2014;60(1):79-82. doi: 10.1093/tropej/fmt075 [published Online First: 2013/09/21]
67. Nankabirwa V, Tumwine JK, Tylleskar T, et al. Perinatal mortality in eastern Uganda: a community based prospective cohort study. *PLoS One* 2011;6(5):e19674. doi: 10.1371/journal.pone.0019674 [published Online First: 2011/05/17]
68. Hinderaker SG, Olsen BE, Bergsjø PB, et al. Perinatal mortality in northern rural Tanzania. *Journal of Health, Population and Nutrition* 2003:8-17.

For peer review only

For peer review only - http://bmjopen.bmj.com/site/about/guidelines.xhtml

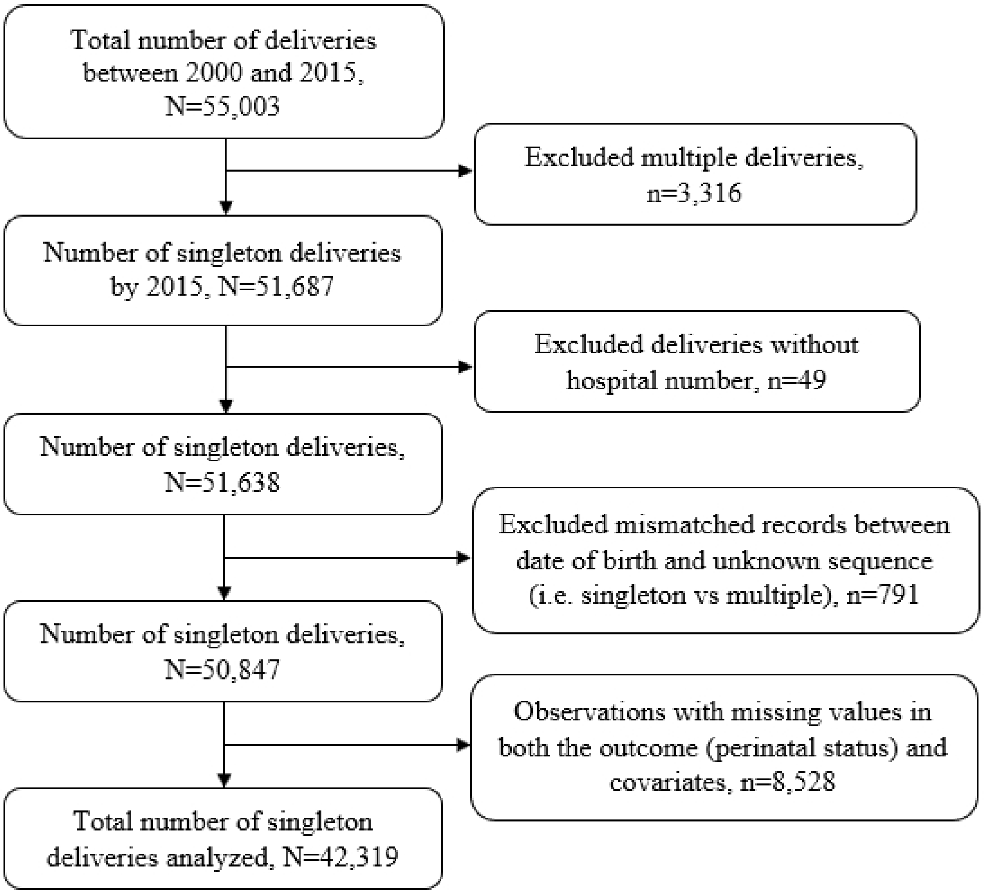

Figure 1: Schematic diagram showing the number of singleton deliveries analyzed, KCMC medical birth registry data, 2000-2015.

146x133mm (96 x 96 DPI)

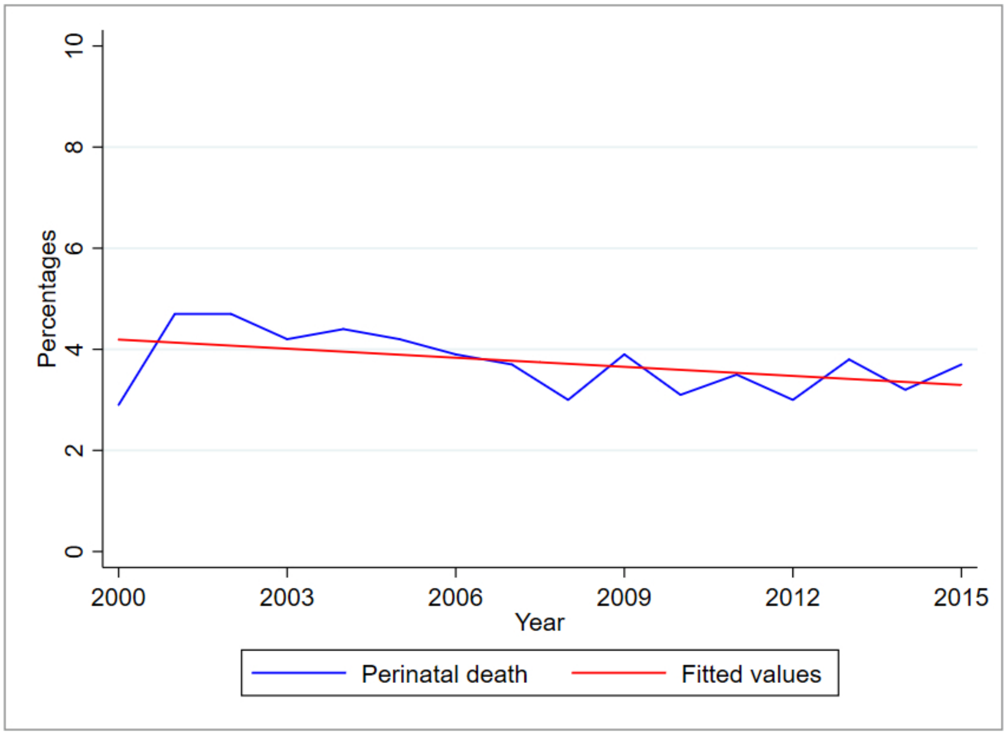

Figure 2: Trends of perinatal death, KCMC Medical Birth Registry data, 2000-2015.

298x217mm (72 x 72 DPI)

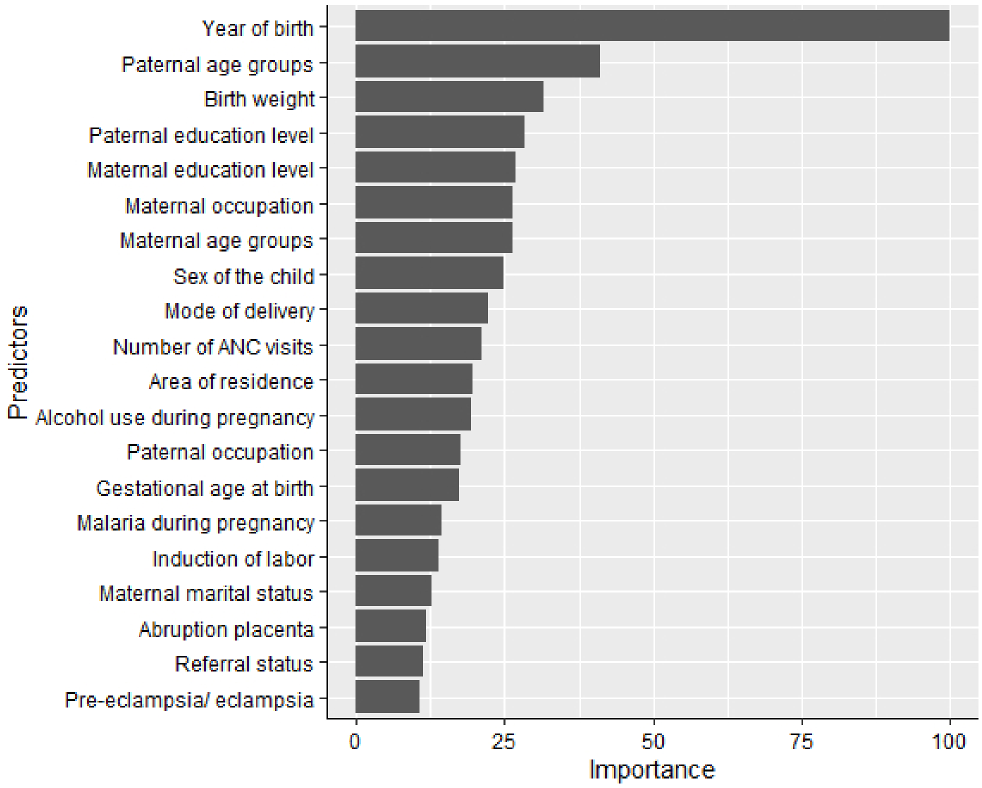

Figure 3: Variable importance of predictors for perinatal death in the random forest model scaled to have a maximum value of 100.

153x123mm (96 x 96 DPI)

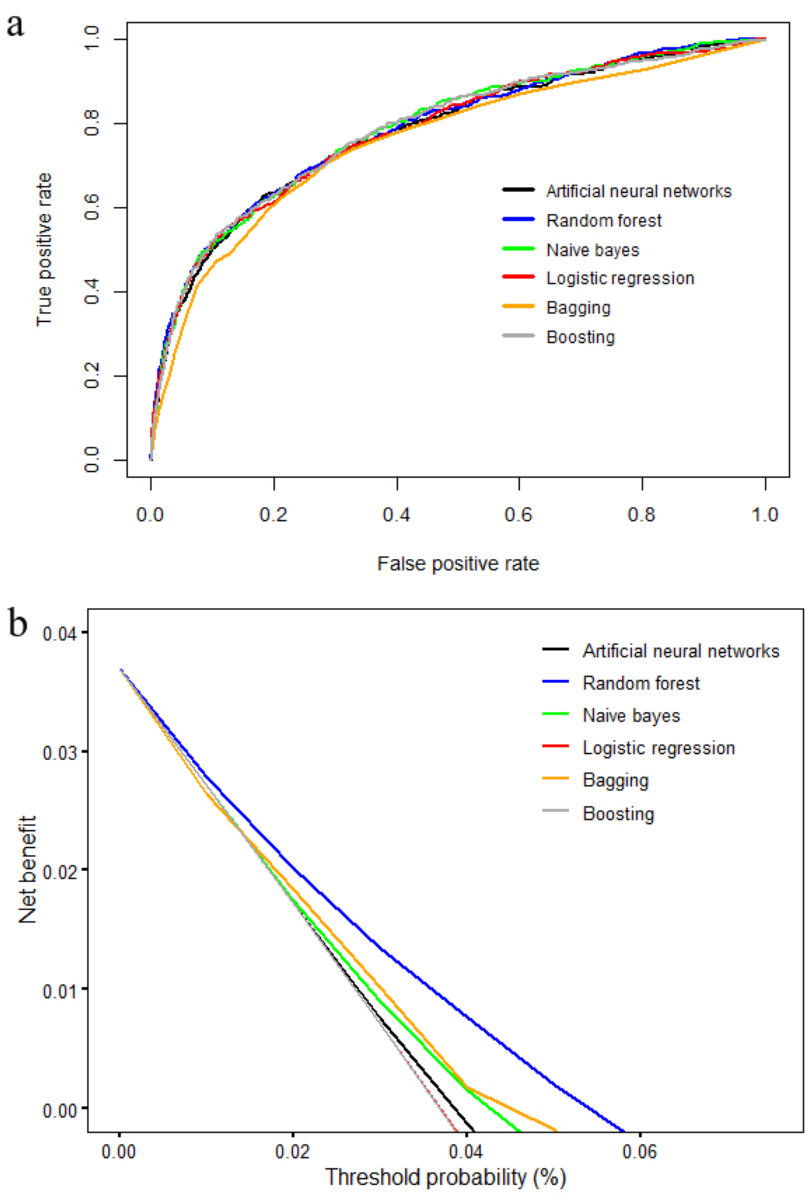

Figure 4: Prediction ability of perinatal deaths comparing different machine learning models in the test set: [a] Receiver-operating-characteristics (ROC) curves. The corresponding values of the area under the receiver-operating-characteristics curve (AUC) for each model are in Table 2. [b] Decision curve analysis. The net benefit of the machine learning models (except for boosting) is larger over a range of threshold probability values compared to that of the logistic regression model.

152x226mm (96 x 96 DPI)

STROBE 2007 (v4) Statement—Checklist of items that should be included in reports of *cohort studies*

Section/Topic	Item #	Recommendation	Reported on page #
**Title and abstract**	1	(*a*) Indicate the study's design with a commonly used term in the title or the abstract	1
		(*b*) Provide in the abstract an informative and balanced summary of what was done and what was found	2
**Introduction**			
Background/rationale	2	Explain the scientific background and rationale for the investigation being reported	3-5
Objectives	3	State specific objectives, including any prespecified hypotheses	1, 4
**Methods**			
Study design	4	Present key elements of study design early in the paper	5
Setting	5	Describe the setting, locations, and relevant dates, including periods of recruitment, exposure, follow-up, and data collection	5
Participants	6	(*a*) Give the eligibility criteria, and the sources and methods of selection of participants. Describe methods of follow-up	5
		(*b*) For matched studies, give matching criteria and number of exposed and unexposed	NA
Variables	7	Clearly define all outcomes, exposures, predictors, potential confounders, and effect modifiers. Give diagnostic criteria, if applicable	6-7
Data sources/ measurement	8*	For each variable of interest, give sources of data and details of methods of assessment (measurement). Describe comparability of assessment methods if there is more than one group	5-6
Bias	9	Describe any efforts to address potential sources of bias	7
Study size	10	Explain how the study size was arrived at	9
Quantitative variables	11	Explain how quantitative variables were handled in the analyses. If applicable, describe which groupings were chosen and why	6-7
Statistical methods	12	(*a*) Describe all statistical methods, including those used to control for confounding	7-9
		(*b*) Describe any methods used to examine subgroups and interactions	NA
		(*c*) Explain how missing data were addressed	NA
		(*d*) If applicable, explain how loss to follow-up was addressed	NA
		(*e*) Describe any sensitivity analyses	9
**Results**			

For peer review only

Participants	13*	(a) Report numbers of individuals at each stage of study—eg numbers potentially eligible, examined for eligibility, confirmed eligible, included in the study, completing follow-up, and analysed	9
		(b) Give reasons for non-participation at each stage	NA
		(c) Consider use of a flow diagram	9
Descriptive data	14*	(a) Give characteristics of study participants (eg demographic, clinical, social) and information on exposures and potential confounders	9-10
		(b) Indicate number of participants with missing data for each variable of interest	9
		(c) Summarise follow-up time (eg, average and total amount)	9
Outcome data	15*	Report numbers of outcome events or summary measures over time	10
Main results	16	(*a*) Give unadjusted estimates and, if applicable, confounder-adjusted estimates and their precision (eg, 95% confidence interval). Make clear which confounders were adjusted for and why they were included	NA
		(*b*) Report category boundaries when continuous variables were categorized	10-12
		(*c*) If relevant, consider translating estimates of relative risk into absolute risk for a meaningful time period	NA
Other analyses	17	Report other analyses done—eg analyses of subgroups and interactions, and sensitivity analyses	13-15
**Discussion**			
Key results	18	Summarise key results with reference to study objectives	16
**Limitations**			
Interpretation	20	Give a cautious overall interpretation of results considering objectives, limitations, multiplicity of analyses, results from similar studies, and other relevant evidence	16-18
Generalisability	21	Discuss the generalisability (external validity) of the study results	18
**Other information**			
Funding	22	Give the source of funding and the role of the funders for the present study and, if applicable, for the original study on which the present article is based	19

*Give information separately for cases and controls in case-control studies and, if applicable, for exposed and unexposed groups in cohort and cross-sectional studies.

Note: An Explanation and Elaboration article discusses each checklist item and gives methodological background and published examples of transparent reporting. The STROBE checklist is best used in conjunction with this article (freely available on the Web sites of PLoS Medicine at http://www.plosmedicine.org/, Annals of Internal Medicine at http://www.annals.org/, and Epidemiology at http://www.epidem.com/). Information on the STROBE Initiative is available at www.strobe-statement.org.

For peer review only

BMJ Open

Prediction of perinatal death using machine learning models: A birth registry-based cohort study in northern Tanzania

Journal:	*BMJ Open*
Manuscript ID	bmjopen-2020-040132.R1
Article Type:	Original research
Date Submitted by the Author:	15-Jul-2020
Complete List of Authors:	Mboya, Innocent; University of KwaZulu-Natal, School of Mathematics, Statistics, and Computer Science; Kilimanjaro Christian Medical University College, Epidemiology and Biostatistics<br>Mahande, Michael Johnson; Kilimanjaro Christian Medical University College, Epidemiology & Biostatistics<br>Mohammed, Mohanad ; University of KwaZulu-Natal, School of Mathematics, Statistics and Computer Science<br>Obure, Joseph; Kilimanjaro Christian Medical Centre, Obstetrics and Gynaecology<br>Mwambi, Henry; University of KwaZulu-Natal, School of Mathematics, Statistics and Computer Science
**Primary Subject Heading**:	Paediatrics
Secondary Subject Heading:	Public health, Obstetrics and gynaecology, Epidemiology, Paediatrics
Keywords:	Reproductive medicine < GYNAECOLOGY, NEONATOLOGY, Prenatal diagnosis < OBSTETRICS, PERINATOLOGY, EPIDEMIOLOGY

SCHOLARONE™
Manuscripts

I, the Submitting Author has the right to grant and does grant on behalf of all authors of the Work (as defined in the below author licence), an exclusive licence and/or a non-exclusive licence for contributions from authors who are: i) UK Crown employees; ii) where BMJ has agreed a CC-BY licence shall apply, and/or iii) in accordance with the terms applicable for US Federal Government officers or employees acting as part of their official duties; on a worldwide, perpetual, irrevocable, royalty-free basis to BMJ Publishing Group Ltd ("BMJ") its licensees and where the relevant Journal is co-owned by BMJ to the co-owners of the Journal, to publish the Work in this journal and any other BMJ products and to exploit all rights, as set out in our licence.

The Submitting Author accepts and understands that any supply made under these terms is made by BMJ to the Submitting Author unless you are acting as an employee on behalf of your employer or a postgraduate student of an affiliated institution which is paying any applicable article publishing charge ("APC") for Open Access articles. Where the Submitting Author wishes to make the Work available on an Open Access basis (and intends to pay the relevant APC), the terms of reuse of such Open Access shall be governed by a Creative Commons licence – details of these licences and which Creative Commons licence will apply to this Work are set out in our licence referred to above.

Other than as permitted in any relevant BMJ Author's Self Archiving Policies, I confirm this Work has not been accepted for publication elsewhere, is not being considered for publication elsewhere and does not duplicate material already published. I confirm all authors consent to publication of this Work and authorise the granting of this licence.

For peer review only

Prediction of perinatal death using machine learning models: A birth registry-based cohort study in northern Tanzania

Innocent B. Mboya[1,2*], Michael J. Mahande[2], Mohanad Mohammed[1], Joseph Obure[3], Henry G. Mwambi[1]

[1]School of Mathematics, Statistics, and Computer Science, University of KwaZulu-Natal, Pietermaritzburg, Private Bag X01, Scottsville 3209, South Africa.

[2]Department of Epidemiology and Biostatistics, Kilimanjaro Christian Medical University College (KCMUCo), P. O. Box 2240, Moshi-Tanzania.

[3]Department of Obstetrics and Gynecology, Kilimanjaro Christian Medical Center (KCMC), P. O. Box 3010, Moshi-Tanzania.

[*]Correspondence

E-mail: ib.mboya@gmail.com

For peer review only

ABSTRACT

Objective: We aimed to determine the key predictors of perinatal deaths using machine learning models compared with the logistic regression model.

Design: A secondary data analysis using the Kilimanjaro Christian Medical Center (KCMC) Medical Birth Registry cohort from 2000 to 2015. We assessed the discriminative ability of models using the area under the receiver operating characteristics curve (AUC) and the net benefit using decision curve analysis.

Setting: The KCMC is a zonal referral hospital located in Moshi Municipality, Kilimanjaro region, Northern Tanzania. The Medical Birth Registry is within the hospital grounds at the Reproductive and Child Health Centre.

Participants: Singleton deliveries (N=42,319) with complete records from 2000 to 2015.

Primary outcome measures: Perinatal death (composite of stillbirths and early neonatal deaths). These outcomes were only captured before mothers were discharged from the hospital.

Results: The proportion of perinatal deaths was 3.7%. There were no statistically significant differences in the predictive performance of four machine learning models except for bagging, which had a significantly lower performance (AUC: 0.76, 95%CI 0.74, 0.79, p=0.006) compared to the logistic regression model (AUC: 0.78, 95%CI 0.76, 0.81). However, in the decision curve analysis, the machine learning models had a higher net benefit (i.e. the correct classification of perinatal deaths considering a trade-off between false-negatives and false-positives – over the logistic regression model across a range of threshold probability values).

Conclusions: In this cohort, there was no significant difference in the prediction of perinatal deaths between machine learning and logistic regression models, except for bagging. The machine learning models had a higher net benefit, as its predictive ability of perinatal death was considerably superior over the logistic regression model. The machine learning models, as demonstrated by our study, can be used to improve the prediction of perinatal deaths and triage for women at risk.

For peer review only - http://bmjopen.bmj.com/site/about/guidelines.xhtml

Strengths and limitations of this study

- The KCMC Medical Birth Registry cohort data provides a rich source of information for monitoring trends, inform clinical and administrative decisions, and enables complex modeling of the key predictors of perinatal deaths, among other adverse pregnancy outcomes.
- While standard regression models such as logistic regression are extensively applied in the literature to predict adverse pregnancy outcomes such as perinatal deaths, the application of machine learning models is limited.
- Machine learning algorithms may improve the prediction ability of perinatal deaths, and enable triage of women at high risk of experiencing adverse perinatal outcomes.
- The birth registry only captured deaths occurring in the KCMC hospital hence might have underestimated the proportion of perinatal deaths.

INTRODUCTION

Neonatal survival is at the heart of sustainable development goals (SDG) agenda[1 2]. The Every Newborn Action Plan to end Preventable Deaths set a goal for all countries to reach the target of ten or less newborn deaths per 1000 live births and ten or less stillbirths per 1000 total births by the year 2035[3]. Furthermore, the United Nations set the target of reducing neonatal mortality to 12 deaths per 1000 live births or fewer by 2030[1]. Globally, neonatal deaths declined by 51% from 5 million in 1990 to 2.5 million in 2017. But this decline has not been realized in low- and middle-income countries (LMICs), which carries the highest burden of neonatal deaths, with south Asia and sub-Saharan Africa accounting for 79% of the total burden of neonatal deaths in 2017[4]. Furthermore, the under-five mortality rate has decreased almost across the world, but the proportions of neonatal deaths remained high in this group[5 6]. Neonatal deaths accounted for 47% of all under-five deaths in 2018, and it has increased from 40% in 1990, with sub-Saharan Africa bearing the highest burden[6]. Globally 2.5 million children died in the first month of life in 2018, with approximately 7,000 newborn deaths every day[6]. Nearly three-quarters of these deaths occur during the first week, with about one million dying on the first day and close to one million dying within the next six days[6].

For peer review only

Globally, more than five million perinatal deaths occur each year[2]. The majority (95%) of these deaths occur in sub-Saharan Africa and Southern Asia[7]. According to the Tanzania Demographic and Health Survey, the perinatal mortality rate has slightly increased from 36 to 39 deaths per 1,000 live births between 2010-11 and 2015-16 survey rounds, respectively relative to under-five mortality[8]. In addition, perinatal mortality rate in Tanzania is the highest in East Africa[7].

Early identification of pregnant women at risk for adverse maternal and perinatal outcomes during the prenatal period and timely provision of high-quality health care services have been reported to improve maternal and newborn survival[9]. Machine learning (hereafter denoted as 'ML') models are methodologies for developing algorithms that learn from existing data to make predictions on new data[9]. ML models have shown better predictive performance over the classical or conventional regression models[10], and they can better handle a significant number of potential predictors. However, there is conflicting evidence of the performance of these models. Previous investigators have demonstrated that, compared with the classical regression models, ML models have superior performance for early differentiation of sepsis and non-infectious systemic inflammatory response syndrome in critically ill children[11], in predicting neonatal and under-five mortality[12-16], and critical care and hospitalization outcomes[10 17 18]. In contrast, other studies have shown no predictive performance benefit of the ML models in prediction of clinical outcomes[9 19].

The first step in addressing high perinatal mortality is the accurate capture and classification of the causes of those deaths across all settings[20]. The World Health Organization (WHO) International Classification of Diseases (ICD-10) is a standardized tool used for the classification of deaths occurring during the perinatal period: ICD-PM[2 20 21]. ML models may be an essential tool in the assessment of risk factors for deaths during the perinatal period and triage pregnant women at high risk of experiencing adverse perinatal outcomes, especially in low resourced settings where the majority of perinatal deaths occur at home [22-25]. Capturing the chain of events that led to the perinatal mortality, from both the maternal and the perinatal side, informs the design and development of preventative and therapeutic measures[2].

Using data from the medical birth registry at Kilimanjaro Christian Medical Center (KCMC) referral hospital in northern Tanzania, we aimed to determine the key predictors of

For peer review only - http://bmjopen.bmj.com/site/about/guidelines.xhtml

perinatal death using machine learning models. Previous studies using the same data[26-31] applied standard regression models to assess risk factors for adverse perinatal outcomes. A major weakness of conventional regression analysis, as opposed to ML models, is that many covariates are excluded based on specific model assumptions. In contrast, ML techniques which are non-parametric in nature find the most predictive groupings of factors based on their frequency and strength of association, with no particular model assumptions [32]. In this study, we compared the predictive performance of the ML models with the conventional regression analysis, particularly logistic regression.

METHODS

Study design, setting, and population

We conducted a retrospective cohort study utilizing data from the KCMC referral hospital, situated in the Moshi Municipality of Kilimanjaro region, Northern Tanzania. The hospital receives deliveries from nearby communities and referral cases from other health care facilities inside the region and the neighboring regions[33]. The hospital has an average annual delivery rate of 4000 births[31 33 34]. The study population was women who delivered singleton babies. We included 42,319 deliveries with complete records between 2000 and 2015. We excluded records with missing values on the outcome (perinatal status) and the covariates as well as pregnancies with multiple gestations to avoid over-representation of high-risk pregnancies[31].

Data source

We used data from the KCMC referral hospital medical birth registry between the years 2000-2015, which were collected among mothers who delivered at the department of obstetrics and gynecology. More description of the KCMC medical birth registry is also available elsewhere[26 28 31 35 36]. Briefly, the KCMC medical birth registry is within hospital grounds at the Reproductive and Child Health Centre. The birth registry has been in operation since the year 2000, established to serve both clinical, administrative, and research purposes[35 36]. Trained midwives collected data using a standardized questionnaire (within 24 hours after delivery or later in case a mother had recovered from complications), after which data is entered into a computerized database located

For peer review only - http://bmjopen.bmj.com/site/about/guidelines.xhtml

at the birth registry. Also, additional data were abstracted from the antenatal (ANC) cards and the hospital medical records of the mother[28].

A unique hospital identification number was assigned to each woman at first admission and used to trace her medical records at later admissions, and further to link records of successive births of the same woman[36]. Data captured information on the background characteristics of mother and father, mother's health before and during present pregnancy, information about delivery including complications, and child characteristics including their status (i.e., whether dead or alive).

Study variables

The main outcome variable in this study was perinatal death which was defined as the number of stillbirths (pregnancy loss that occurs after seven months of gestation) and early neonatal deaths (deaths of live births within the first seven days of life)[8 37]. The perinatal death was coded as binary, i.e., 'yes' if death occurred during the perinatal period and 'no' if otherwise. This outcome only captured deaths that occurred within the hospital before the discharge of mothers. There are no follow-up mechanisms for deaths that occur outside the health facility (KCMC hospital).

We included a total of 32 predictor variables for the machine learning models. Previous literature informed the selection of these variables[4 27 38-45], most of which are available in the birth registry. These included maternal and paternal background characteristics; age in years, area of residence (rural vs. urban), highest education level (none, primary, secondary and higher), marital status (single, married and widow/divorced), and occupation (unemployed, employed and others). Further, specific characteristics of the mother included referral status (whether referred for delivery or not), and the number of antenatal care visits (<4 and ≥4 visits).

We excluded maternal body mass index (BMI) and HIV status because they contributed to nearly 47% of all missing values in the dataset. Maternal health during pregnancy included; alcohol consumption, smoking, gestational diabetes, diabetes, hypertension, pre-eclampsia/eclampsia, bleeding (i.e., the woman observed blood from the vagina at any time during the pregnancy), anemia, malaria, and systemic infections/sepsis. Variables with information concerning delivery included; induction of labor (yes or no), mode of delivery

For peer review only - http://bmjopen.bmj.com/site/about/guidelines.xhtml

(vaginal vs. caesarean section), presentation (breech vs. cephalic), complications during birth, particularly premature rupture of the membranes (PROM), postpartum hemorrhage (PPH), placenta previa, and placenta abruption, all categorized as yes and no. Gestational age at birth was estimated based on the date of the last menstrual period and recorded in full weeks. Preterm was defined as babies born alive before 37 weeks of pregnancy are completed[46]. Child characteristics included sex (male or female), low birth weight defined as an infant birth weight of less than 2500g[27 47], and year of birth.

Statistical and computational analysis

Data were cleaned and then analyzed using Stata version 15.1[48]. Categorical variables were summarized using frequencies and proportions. The Chi-square (χ^2) statistic was used to test the relationships between a set of independent variables and perinatal death. For the ML models (i.e., from feature selection, training, testing, and comparison of the predictive performance of the machines), we used R version 3.6.3[49]. The training dataset contained 70% of randomly selected samples used to develop six different ML models to predict perinatal death. These are artificial neural networks (ANN), random forests (RF), Naïve Bayes (NB), bagged trees, boosting, and the logistic regression (Lreg) model. We used the *caret* package to implement these models in R.

Briefly, ANN is a method constructed from three layers of connected nodes: input, hidden, and output[50]. The input where each input variable appears as a node; the hidden layer contains several nodes determined during the model tuning phase. In contrast, the output layer contains several nodes equal to the number of classes to be predicted[51]. Between these layers there are weighted links[9 50 51], the hidden layer receives a sum of the multiplication of the input variables with associated weights values plus the bias[50 51]. This value is entered into an activation function, such as a logistic or sigmoid function, to decide the class prediction. Outputs of the network are interpreted as class probabilities and sum to one[51]. We used *nnet* package to construct the ANN model.

RF is an extension of classification and regression trees (CART)[9 10 51 52]. RF performance is better compared to bagged trees because it decorrelates the trees[53], hence improves accuracy[52]. Several forests of decision trees are grown using a random bootstrapped training sample. Also, instead of using all the variables/features in each tree, a random sample of variables are selected

For peer review only - http://bmjopen.bmj.com/site/about/guidelines.xhtml

and tested at each split in each tree[10 51 52]. The prediction is made for unobserved data by taking a majority vote of the individual trees[51 52]. We used *randomForest* package to construct the RF model. NB is an effective classifier[50] due to its simplicity, exhibiting a surprisingly competitive predictive accuracy[54]. NB uses probability theory to find the most possible sample class in a classification problem. NB has two assumptions; (1) each attribute is conditionally independent of the other attributes given the class, and (2) all the attributes have an impact on the class[51 54]. We used *naivebayes* package to construct the NB model.

Logistic regression (Lreg) is a standard multivariate classification method. It arises from the desire to model the posterior probabilities via linear functions in covariates, such that besides predicting class labels, it provides a probabilistic interpretation of this labeling[53 55 56]. Lreg uses a sigmoid function instead of a linear function to map predictions to probabilities between 0 and 1[53]. We used *glm* method to construct the Lreg model. Bagging, or bootstrap aggregation and boosting are general techniques for improving prediction rules and accuracy of the resulting predictions, by reducing the associated variance of prediction[53 57]. Bagging divides the available data into many bootstrap samples and then train a separate model for each bootstrap, and then make a final prediction by averaging and voting for regression and classification, respectively[57]. Boosting, on the other hand, is a committee-based approach that uses a weighted average of prediction from various samples. The incorrectly predicted cases from a given step are given a higher weight during the next step. Thus, it is an iterative procedure, incorporating weights, as opposed to simple averaging of predictions[57]. We used *treebag* method and *gbm* package to construct the bagging and boosting models, respectively.

In the training set, parameter tuning and cross-validation aim to find a balance between building a model that can classify the training data effectively without overfitting to the random fluctuations[51]. For each ML model, we used 10-fold cross-validation as a resampling method, where the training set is divided equally into ten parts (folds). Therefore, every nine folds are used together for training the model and the remaining one-fold for testing. This training-testing process is repeated ten times. We performed feature selection using the RF algorithm. After selecting the most important features, we retained in the dataset and used them for analysis in both the training and testing data for all models. We used the Synthetic Minority Over-sampling Technique (SMOTE) method[58 59] to address the class imbalance in the outcome (i.e., the low

For peer review only

For peer review only - http://bmjopen.bmj.com/site/about/guidelines.xhtml

proportion of perinatal deaths), by specifying the additional sampling to be "smote" on train control parameter specifications. SMOTE is a method that produces artificial minority samples by interpolating between existing minority samples and their nearest minority neighbors[58 59].

Using the testing set (30% of the remaining randomly selected sample), we computed the predictive performance of the six models (including logistic regression model) from the training set using the area under the receiver-operating-characteristics curve (AUC). We used the *ROCR* package for plotting ROC curves, obtaining the AUC values, and comparison of models using AUC values. We also used measures from the confusion matrix results (i.e., accuracy, sensitivity, specificity, positive and negative predictive values), and the net benefit through decision curve analysis[60 61] – which quantifies whether a machine provides a relevant improvement in the prediction. We used *epiR* package to obtain confidence intervals for the performance measures and *DCA* package (*http://www.decisioncurveanalysis.org*) for decision curve analysis. We further used *ggplot2* package to plot the decision curves. A good model will have a higher net benefit[60]. We used Delong's test to compare the receiver-operating-characteristics curve (ROC) between models, where, a p-value of <0.05 was considered statistically significant. The variable importance is a scaled measure with a maximum value of 100[17]. The R code used for this analysis is shown in supplementary material 1.

Patient and public involvement

There was no patient and public involvement.

RESULTS

Characteristics of study participants

The characteristics of the participants are shown in Table **1**. A total of 55,003 total deliveries were recorded at the KCMC medical birth registry from 2000 to 2015. Of these, we excluded 3,316 (6%) multiple gestations (to avoid over-representation of high-risk pregnancies)[31], 49 (0.1%) records missing maternal identification numbers (hence could not be linked to child records), 791 (1.4%) records with a mismatch between the date of birth and unknown sequence (i.e., singleton vs. multiple births). We further excluded a total of 8,528 (15.5%) observations

For peer review only - http://bmjopen.bmj.com/site/about/guidelines.xhtml

with missing values in both the outcome (perinatal status) and covariates. We, therefore, analyzed data for a total of 42,319 singleton deliveries with complete records (Figure **1**).

Figure 1: Schematic diagram showing the number of singleton deliveries analyzed, KCMC medical birth registry data, 2000-2015.

The overall proportion of perinatal death among 42,319 singleton deliveries in this study was 3.7%. The proportion of perinatal deaths among mothers aged 20-34, 35-39, and 40+ years was 3.5%, 4.7%, and 5.8%, respectively. Mothers with no education (5.6%) and those with primary education level (4.4%), who resided in rural areas (4.8%), had less than four antenatal care (ANC) visits (5.9%), and those referred for delivery (7.8%) had a higher proportion of perinatal death. Among fathers, a higher proportion of perinatal death is among those aged 35+ years (4.1%), with no (9.6%) or with primary education level (4.6%) as well as those who were unemployed (5.7%), Table **1**.

Furthermore, the most common obstetric care and complications in this birth cohort included induction of labor (22.7%), malaria (13.2%), preterm birth (10.8%), and LBW (10.2%). About 4% of mothers in this cohort experienced pre-eclampsia/ eclampsia during pregnancy. Less than half of all children were females. The proportion of perinatal death among women who experienced induction of labor, with malaria, delivered preterm, delivered LBW baby, and experienced pre-eclampsia/ eclampsia during pregnancy was 4.8%, 3.8%, 14.2%, 17.6%, and 12.5%, respectively. The proportion of perinatal death is almost similar among males (3.8%) compared to females (3.6%) children in this cohort (Table **1**).

For peer review only

For peer review only - http://bmjopen.bmj.com/site/about/guidelines.xhtml

Table 1: Characteristics of study participants (N=42,319)

Characteristics	Total	Perinatal death	P-value*	Characteristics	Total	Perinatal death	P-value*
Maternal	**n (%)**	**n (%)**		***Obstetrics***	**n (%)**	**n (%)**	
**Age (years)**			<0.001	**Gestational diabetes**			0.89
15-19	3470 (8.2)	99 (2.9)		No	42288 (99.9)	1560 (3.7)	
20-34	32675 (77.2)	1158 (3.5)		Yes	31 (0.1)	1 (3.2)	
35-39	4984 (11.8)	235 (4.7)		**Diabetes**			0.002
40+	1190 (2.8)	69 (5.8)		No	42240 (99.8)	1553 (3.7)	
**Education level**			<0.001	Yes	79 (0.2)	8 (10.1)	
None	567 (1.3)	32 (5.6)		**Hypertension**			<0.001
Primary	23010 (54.4)	1019 (4.4)		No	42241 (99.8)	1550 (3.7)	
Secondary	5275 (12.5)	159 (3.0)		Yes	78 (0.2)	11 (14.1)	
Higher	13467 (31.8)	351 (2.6)		**Bleeding**			<0.001
**Occupation**			0.37	No	41897 (99.0)	1528 (3.6)	
Unemployed	9316 (22.0)	365 (3.9)		Yes	422 (1.0)	33 (7.8)	
Employed	30061 (71.0)	1085 (3.6)		**Anemia**			0.004
Others	2942 (7.0)	111 (3.8)		No	41661 (98.4)	1523 (3.7)	
**Marital status**			0.89	Yes	658 (1.6)	38 (5.8)	
Single	4954 (11.7)	186 (3.8)		**Malaria**			0.79
Married	37300 (88.1)	1372 (3.7)		No	36746 (86.8)	1352 (3.7)	
Widowed/Divorced	65 (0.2)	3 (4.6)		Yes	5573 (13.2)	209 (3.8)	
**Area of residence**			<0.001	**Sepsis/ infections**			0.43
Urban	25056 (59.2)	725 (2.9)		No	41588 (98.3)	1538 (3.7)	
Rural	17263 (40.8)	836 (4.8)		Yes	731 (1.7)	23 (3.1)	
**Alcohol consumption during pregnancy**			0.001	***Complications***			
No	30759 (72.7)	1191 (3.9)		**Pre-eclampsia/ eclampsia**			<0.001
Yes	11560 (27.3)	370 (3.2)		No	40668 (96.1)	1355 (3.3)	
**Smoking during pregnancy**			0.97	Yes	1651 (3.9)	206 (12.5)	
Yes	53 (0.1)	2 (3.8)		**Induction of labor**			<0.001
No	42266 (99.9)	1559 (3.7)		No	32732 (77.3)	1105 (3.4)	
**Number of ANC visits**			<0.001	Yes	9587 (22.7)	456 (4.8)	
≥4	28742 (67.9)	760 (2.6)		**PROM**			0.006
<4	13577 (32.1)	801 (5.9)		No	41416 (97.9)	1543 (3.7)	

For peer review only

For peer review only - http://bmjopen.bmj.com/site/about/guidelines.xhtml

**Referred for delivery**			<0.001	Yes	903 (2.1)	18 (2.0)	
No	32762 (77.4)	819 (2.5)		**PPH**			<0.001
Yes	9557 (22.6)	742 (7.8)		No	42091 (99.5)	1516 (3.6)	
Paternal characteristics				Yes	228 (0.5)	45 (19.7)	
**Age (years)**			0.001	**3-4-degree tear**			0.49
<25	3938 (9.3)	122 (3.1)		No	42305 (99.9)	1560 (3.7)	
25-29	10593 (25.0)	346 (3.3)		Yes	14 (0.1)	1 (7.1)	
30-34	12303 (29.1)	457 (3.7)		**Abruption placenta**			<0.001
35+	15485 (36.6)	636 (4.1)		No	42193 (99.7)	1490 (3.5)	
**Education level**			<0.001	Yes	126 (0.3)	71 (56.3)	
None	281 (0.7)	27 (9.6)		**Placenta previa**			0.04
Primary	18987 (44.9)	868 (4.6)		No	42245 (99.8)	1555 (3.7)	
Secondary	4565 (10.8)	154 (3.4)		Yes	74 (0.2)	6 (8.1)	
Higher	18486 (43.7)	512 (2.8)		**Presentation**			<0.001
**Occupation**			<0.001	Cephalic	41833 (98.9)	1459 (3.5)	
Unemployed	5710 (13.5)	323 (5.7)		Breach/ Transverse	486 (1.1)	102 (21.0)	
Employed	36102 (85.3)	1218 (3.4)		**Gestational age at birth**			<0.001
Others	507 (1.2)	20 (3.9)		Term birth (>=37 weeks)	37764 (89.2)	914 (2.4)	
				Preterm birth (<37 weeks)	4555 (10.8)	647 (14.2)	
				**Birth weight**			<0.001
				NBW	37991 (89.8)	801 (2.1)	
				LBW	4328 (10.2)	760 (17.6)	
				**Child's sex**			0.42
				Female	20430 (48.3)	738 (3.6)	
**Total**	**42319**	**1561 (3.7%)**		Male	21889 (51.7)	823 (3.8)	

***P-value based on the Chi-square test.**

For peer review only - http://bmjopen.bmj.com/site/about/guidelines.xhtml

For peer review only

The trends in the proportion of perinatal deaths that occurred at KCMC between the years 2000-2015 are shown in Figure **2**. Overall, the proportion of perinatal deaths has slightly declined over the years by 6% (95%CI, 0.3%, 12.3%), though this decline was not statistically significant (p=0.06).

Figure 2: Trends of perinatal death, KCMC Medical Birth Registry data, 2000-2015.

Variable importance

We used the random forest algorithm for feature/ variable selection. This model selected a total of 20 important predictors (Figure **3**) based on its threshold measure of importance out of the 32 variables. We used these 20 variables in all the subsequent analysis for all models in both training and testing sets.

Figure 3: Variable importance of predictors for perinatal death in the random forest model scaled to have a maximum value of 100.

Predicting perinatal deaths

The discriminatory abilities of all models for the prediction of perinatal death are in Figure **4a** and Table **2**. There were no significant differences (p>0.05) in the area under the receiver-operating characteristics curve (AUC) between logistic regression with random forest (RF), artificial neural networks (ANN), boosting, and naïve Bayes (NB). However, bagging had significantly lower predictive performance (AUC: 0.76, 95%CI 0.74, 0.79, p=0.006) compared to the logistic regression model (AUC: 0.78, 95%CI 0.76, 0.81). Furthermore, the ANN model (Sensitivity: 0.60, 95% CI 0.55, 0.64) and NB model (Sensitivity: 0.57, 95% CI 0.52, 0.62) had slightly higher sensitivity compared to logistic regression (Sensitivity: 0.56, 95%CI 0.51, 0.60) while boosting (Specificity: 0.89 95%CI 0.88, 0.89) and RF (Specificity: 0.88, 95%CI 0.88, 0.89) had slightly higher specificity compared to logistic regression (Specificity: 0.87, 95%CI 0.86, 0.88). Due to the low prevalence of perinatal deaths (3.7%), all models had high negative predictive values (NPV: 0.98, 95%CI 0.98, 0.98).

With regard to the number of actual and predicted outcomes (Table **3**), all models correctly predicted perinatal deaths by more than half of 468 deaths in the testing set. The

For peer review only - http://bmjopen.bmj.com/site/about/guidelines.xhtml

numbers of correct classification were higher in the ANN 280 (59.8%) and NB 267 (57.1%), followed by the logistic regression model 261 (55.8%) and Bagging 260 (55.6%). The decision curve analysis (Figure 4b) demonstrated that the net benefit of the RF model surpassed that of other machine learning models, including logistic regression for all threshold values, indicating that the RF model is more superior in predicting the risk of perinatal deaths in this cohort. The accuracy of the RF model was 0.87, 95%CI (0.86, 0.87), compared to 0.87, 95%CI (0.87, 0.88) for boosting and 0.86, 95%CI (0.85, 0.86) for the logistic regression model (Table **2**). Furthermore, other ML models (except for boosting) demonstrated high net benefit over a range of threshold probability values relative to that of the logistic regression model. Also, the random forest model had a superior net benefit over all models (Figure **4b**).

For peer review only

For peer review only - http://bmjopen.bmj.com/site/about/guidelines.xhtml

Table 2: Prediction performance of the reference and machine learning models in the test set

Model	Logistic Regression	Artificial Neural Network	Random Forests	Naïve Bayes	Bagging	Boosting
ACC	0.86 (0.85, 0.86)	0.83 (0.82, 0.83)	0.87 (0.86, 0.87)	0.84 (0.83, 0.85)	0.82 (0.81, 0.83)	0.87 (0.87, 0.88)
AUC	0.78 (0.76, 0.81)	0.78 (0.76, 0.80)	0.79 (0.76, 0.81)	0.79 (0.76, 0.81)	0.76 (0.74, 0.79)	0.79 (0.76, 0.81)
P-value*	Reference	0.59	0.37	0.65	0.006	0.20
Sensitivity	0.56 (0.51, 0.60)	0.60 (0.55, 0.64)	0.54 (0.49, 0.58)	0.57 (0.52, 0.62)	0.55 (0.50, 0.59)	0.54 (0.49, 0.58)
Specificity	0.87 (0.86, 0.88)	0.84 (0.83, 0.84)	0.88 (0.88, 0.89)	0.85 (0.84, 0.86)	0.83 (0.82, 0.84)	0.89 (0.88, 0.89)
PPV	0.14 (0.12, 0.16)	0.12 (0.11, 0.14)	0.15 (0.13, 0.17)	0.13 (0.11, 0.14)	0.11 (0.10, 0.12)	0.15 (0.14, 0.17)
NPV	0.98 (0.98, 0.98)	0.98 (0.98, 0.98)	0.98 (0.98, 0.98)	0.98 (0.98, 0.98)	0.98 (0.98, 0.98)	0.98 (0.98, 0.98)

Abbreviations: ACC, Accuracy; AUC, Area Under the Curve; PPV, Positive Predictive Value; NPV, Negative Predictive Value
*We calculated p-values to compare the area under the receiver-operating-characteristics curve (AUC) of logistic with each machine learning model

Table 3: The number of actual and predicted outcomes of prediction models in the test set

Prediction Model	Classification	Perinatal status	
		Alive	Died
	Actual number of events	12227	468
Logistic Regression	Correctly predicted outcome	10627	261
	Incorrectly predicted outcome	1600	207
Artificial Neural Network	Correctly predicted outcome	10225	280
	Incorrectly predicted outcome	2002	188
Random Forests	Correctly predicted outcome	10774	251
	Incorrectly predicted outcome	1453	217
Naïve Bayes	Correctly predicted outcome	10386	267
	Incorrectly predicted outcome	1841	201
Bagging	Correctly predicted outcome	10175	260
	Incorrectly predicted outcome	2052	208
Boosting	Correctly predicted outcome	10852	252
	Incorrectly predicted outcome	1375	216

For peer review only - http://bmjopen.bmj.com/site/about/guidelines.xhtml

Figure 4: Prediction ability of perinatal deaths comparing different machine learning models in the test set: [a] Receiver-operating-characteristics (ROC) curves. The corresponding values of the area under the receiver-operating-characteristics curve (AUC) for each model are in Table 2. [b] Decision curve analysis. The net benefit of the machine learning models (except for boosting) is larger over a range of threshold probability values compared to that of the logistic regression model.

DISCUSSION

In this study, the perinatal death was predicted using five ML models (Artificial Neural Networks, Random Forests, Naïve Bayes, Bagging, and Boosting). There were no differences in the predictive performance between ML models except for bagging, which had a lower predictive performance. The artificial neural networks and naïve Bayes had higher sensitivity compared to the logistic regression, and other ML models. Specificity for all models was high, mainly due to the low prevalence of perinatal deaths in this cohort. Additionally, results from the decision curve analysis revealed that the ML models (except for boosting) had a higher net benefit over a range of threshold probability values compared to the logistic regression model, indicating high accuracy. The random forest model demonstrated a superior net benefit over other models.

In the present study, maternal characteristics before and during pregnancy, pregnancy history, and paternal characteristics identified pregnancies at high risk of experiencing adverse perinatal outcomes that might need close clinical follow-ups. It is worth noting here that paternal age and education level were highly predictive of perinatal death more than the known pregnancy-related conditions or complications such as prematurity. Previous literature shows that paternal characteristics, particularly advanced paternal age, increase the risk of adverse perinatal outcomes, such as low birth weight, prematurity, small for gestational age, and low Apgar scores[62-64], despite conflicting evidence from other studies[65]. Furthermore, studies using data from the KCMC Medical Birth Registry (same data source to the current study) focused on modelling the association between maternal and pregnancy-related characteristics and complications during pregnancy and childbirth with the risk of adverse perinatal outcomes[26-31] but ignored paternal characteristics. Despite challenges in male involvement in pregnancy and

For peer review only - http://bmjopen.bmj.com/site/about/guidelines.xhtml

childbirth in Tanzania[66 67], their participation is critical to improving maternal and child health outcomes.

On top of clinicians' judgment, previous investigators applied standard regression models in prediction of risk for adverse perinatal outcomes, particularly perinatal death[29 39-45 68-73]. We found no differences in the predictive performance of the ML models, except for bagging, which had lower predictive capacity. The sensitivity of the machine learning models was also almost comparable to that of logistic regression, which indicates that both models correctly classified perinatal deaths. Our finding is consistent with a recent systematic review that showed no performance benefit of ML models over logistic regression for the prediction of clinical outcomes[19]. The possible explanation for lack of differences in the performance between the compared models could be attributed to the low proportion of outcome and exposures in this study, as well as data quality and recording challenges inherent in registry-based studies.

In contrast, some previous investigators have demonstrated that ML models offer better predictions of clinical or adverse pregnancy outcomes compared to classical regression models [13-16]. The application of ML models may improve the classification of adverse events occurring during the perinatal period and, therefore, assist in triaging and provision of close clinical follow-up for women at high-risk. Other studies also provide evidence of improved prediction of under-five and neonatal mortality[12-15] using ML models. The utility of these models may, therefore, improve the prediction of adverse pregnancy outcomes as opposed to standard regression models.

In this study, the decision curve analysis that accounts for the impact of false-negative and false-positive misclassification errors showed superior predictive performance of the ML approaches over the logistic regression model. This demonstrates a higher net benefit for the prediction of perinatal deaths. The higher net benefit in the prediction ability of the ML approaches has also been documented elsewhere[10 17]. This is because ML approaches can incorporate the high order nonlinear interactions between predictors, which cannot be addressed by traditional modeling approaches, including the logistic regression model. Furthermore, the use of cross-validation is also known to reduce potential overfitting in ML models. It is important to note that ML approaches are, to a large extent, non-parametric as opposed to the logistic regression model that relies on strong distributional assumptions.

For peer review only - http://bmjopen.bmj.com/site/about/guidelines.xhtml

The strength of this study is that it is the first to apply modern ML approaches to predict perinatal deaths, particularly in Tanzania and to a large extent sub-Saharan Africa, compared with the classical logistic regression model. Our study demonstrated that ML models might be used to improve the prediction of perinatal deaths and triage of women at risk. We also used the SMOTE balancing technique to avoid the bias of the model toward skewed data (reduce over-fitting), hence improving the prediction accuracy of the ML algorithm[14 58 59]. However, SMOTE is not very effective for high dimensional data[74 75]. Our study also had some limitations that are worth considering when interpreting the results. Firstly, we excluded observations with missing values in both the outcome and exposures from the analysis, a problem inherent in cohort studies, including birth registries, which may lead to under-estimation of the proportion of perinatal death. Two excluded variables (maternal BMI and HIV status) have been associated with perinatal and under-five deaths[4 6 38]; hence their exclusion might increase the risk of residual confounding bias. The effect of exclusion of these two variables and missing values to predict perinatal deaths remains unquantified.

Secondly, selection bias/ or referral bias is a common problem in hospital-based studies, which affects the generalization of findings to the general population. This might also be the case in the present study. However, our findings might reflect a similar setting in Tanzania and probably in other sub-Saharan African countries. Thirdly, the KCMC Medical Birth Registry cohort only captures perinatal deaths occurring in the health facility (KCMC hospital), which may underestimate the observed perinatal deaths in the wider population. Currently, the hospital has no mechanisms to follow-up the birth outcomes from deliveries that occur at home and post-discharge outcomes of the babies after mothers are discharged from the hospital within the first week, especially within the KCMC hospital catchment area. Future extensions include ways of handling missing values before applying the machine learning algorithms to predict perinatal death and other adverse pregnancy outcomes.

CONCLUSION

The ML models (except for bagging) performed equally with the logistic regression model to predict perinatal deaths using maternal, paternal, and obstetric factors in this cohort. The ML models, however, have a higher net benefit, demonstrating superiority in the prediction of

For peer review only - http://bmjopen.bmj.com/site/about/guidelines.xhtml

perinatal death. Furthermore, the random forest model also demonstrated superior performance over other ML models. These models are a useful and alternative strategy over the standard logistic regression model to predict perinatal deaths, considering the richness of the medical birth registries. Moreover, the ML models are capable of handling many predictors at the same time, which is crucial in capturing multiple risk factors for adverse perinatal outcomes such as perinatal deaths. The application of ML models may, therefore, increase the prediction ability of adverse perinatal outcomes and thereby helping in triage women most at risk.

Acknowledgements

We would like to acknowledge the midwives who participated in data collection and all women and children whose information enabled the availability of data used in this study. The authors also thank the staff at the Birth Registry for capturing these data in the electronic system. We also appreciate the Centre for International Health at the University of Bergen in Norway and the Department of Obstetrics and Gynecology of the KCMC hospital in Tanzania for establishing the KCMC medical birth registry, which facilitated the availability of data to conduct this study.

Author contributions

All authors made a substantial contribution to this study. IBM, MJM, JO and HGM designed the study. IBM and MM analyzed the data. MJM, JO and HGM commented on the manuscript. IBM drafted the manuscript and had primary responsibility for the final content. All authors reviewed the drafts of this manuscript and approved the final version for submission.

Funding

This work was supported through the DELTAS Africa Initiative Grant No. 107754/Z/15/ZDELTAS Africa SSACAB. The DELTAS Africa Initiative is an independent funding scheme of the African Academy of Sciences (AAS)'s Alliance for Accelerating Excellence in Science in Africa (AESA) and supported by the New Partnership for Africa's Development Planning and Coordinating Agency (NEPAD Agency) with funding from the Wellcome Trust (Grant No. 107754/Z/15/Z) and the UK government. The views expressed in this publication are those of the author(s) and not necessarily those of AAS, NEPAD Agency, Wellcome Trust or the UK government. The Norwegian Council for Higher Education's Program for Development Research (NUFU) funded the establishment of the birth registry at the

For peer review only - http://bmjopen.bmj.com/site/about/guidelines.xhtml

Kilimanjaro Christian Medical Center (KCMC). The funders had no role in study design, data collection, and analysis, decision to publish, or preparation of the manuscript.

Competing interests

None declared.

Patient consent for publication

Not required.

Ethics approval

This study was approved by the Kilimanjaro Christian Medical College Research Ethics and Review Committee (KCMU-CRERC) with approval number 2424. Before data collection, mothers provided oral informed consent after receiving information about the purpose of the study, questionnaire, and its contents as well as the intention to gather new knowledge, which will, in turn, benefit mothers and children in the future. Mothers were informed that participation was voluntary and had no implications on the care they would receive. Following consent, mothers were free to refuse to reply to single questions. For privacy and confidentiality, unique identification numbers were used to identify and then link mothers with child records. There was no person-identifiable information in any electronic database, and instead, unique identification numbers were used. Necessary measures were taken by midwives to ensure privacy during the interview process.

Data availability statement

The KCMC medical birth registry data contains potentially identifying and sensitive patient information. This has also been stipulated by the local Institutional Review Board of KCMC hospital and the National Ethics Committee in Norway when establishing this birth registry. Permission to use the data in this study was made through the Kilimanjaro Christian Medical University College Research and Ethics Review Committee, and received an approval number 2424. The authors do not have the legal right to share the data publicly. All data requests can be sent to the Executive Director of the KCMC referral hospital, P. O. Box 3010, Moshi, Tanzania, Email: kcmcadmin@kcmc.ac.tz or through the corresponding author.

For peer review only

ORCID iDs

Innocent B. Mboya: https://orcid.org/0000-0001-9861-5879

Michael J. Mahande: http://orcid.org/0000-0002-7750-7657

Mohanad Mohammed: https://orcid.org/0000-0001-5336-0286

Henry G. Mwambi: https://orcid.org/0000-0001-9654-400X

REFERENCES

1. United Nations Development Program. Sustainable development goals: United Nations; 2019 [Available from: https://www.undp.org/content/undp/en/home/sustainable-development-goals/goal-3-good-health-and-well-being.html#targets accessed August 14 2019.
2. World Health Organization. The WHO application of ICD-10 to deaths during the perinatal period: ICD-PM. Geneva: World Health Organization, 2016.
3. World Health Organization. Every newborn: an action plan to end preventable deaths. Geneva: World Health Organization, 2014.
4. Hug L, Alexander M, You D, et al. National, regional, and global levels and trends in neonatal mortality between 1990 and 2017, with scenario-based projections to 2030: a systematic analysis. *The Lancet Global Health* 2019;7(6):e710-e20.
5. Burstein R, Henry NJ, Collison ML, et al. Mapping 123 million neonatal, infant and child deaths between 2000 and 2017. *Nature* 2019;574(7778):353-58.
6. UNICEF. Levels & Trends in Child Mortality: Report 2019. Estimates Developed by the UN Inter-agency Group for Child Mortality Estimation: United Nations Children's Fund, 2019.
7. Akombi BJ, Renzaho AM. Perinatal Mortality in Sub-Saharan Africa: A Meta-Analysis of Demographic and Health Surveys. *Annals of global health* 2019;85(1)
8. MoHCDGEC, MoH, NBS, et al. Tanzania Demographic and Health Survey and Malaria Indicator Survey (TDHS-MIS) 2015-16. Dar es Salaam, Tanzania and Rockville, Maryland, USA, 2016.
9. Kuhle S, Maguire B, Zhang H, et al. Comparison of logistic regression with machine learning methods for the prediction of fetal growth abnormalities: a retrospective cohort study. *BMC pregnancy and childbirth* 2018;18(1):1-9.
10. Raita Y, Goto T, Faridi MK, et al. Emergency department triage prediction of clinical outcomes using machine learning models. *Crit Care* 2019;23(1):64. doi: 10.1186/s13054-019-2351-7 [published Online First: 2019/02/24]
11. Lamping F, Jack T, Rübsamen N, et al. Development and validation of a diagnostic model for early differentiation of sepsis and non-infectious SIRS in critically ill children-a data-driven approach using machine-learning algorithms. *BMC pediatrics* 2018;18(1):112.
12. Nasejje JB, Mwambi H. Application of random survival forests in understanding the determinants of under-five child mortality in Uganda in the presence of covariates that satisfy the proportional and non-proportional hazards assumption. *BMC Res Notes* 2017;10(1):459. doi: 10.1186/s13104-017-2775-6 [published Online First: 2017/09/09]
13. Houweling TA, van Klaveren D, Das S, et al. A prediction model for neonatal mortality in low-and middle-income countries: an analysis of data from population surveillance sites in India, Nepal and Bangladesh. *International journal of epidemiology* 2019;48(1):186-98.

For peer review only

14. Hoodbhoy Z, Noman M, Shafique A, et al. Use of machine learning algorithms for prediction of fetal risk using cardiotocographic data. *International Journal of Applied and Basic Medical Research* 2019;9(4):226.
15. Muktan D, Singh RR, Bhatta NK, et al. Neonatal mortality risk assessment using SNAPPE- II score in a neonatal intensive care unit. *BMC Pediatr* 2019;19(1):279. doi: 10.1186/s12887-019-1660-y [published Online First: 2019/08/15]
16. Lee SM, Lee MH, Chang YS, et al. The Clinical Risk Index for Babies II for Prediction of Time-Dependent Mortality and Short-Term Morbidities in Very Low Birth Weight Infants. *Neonatology* 2019;116(3):244-51. doi: 10.1159/000500270 [published Online First: 2019/07/16]
17. Goto T, Camargo CA, Jr., Faridi MK, et al. Machine Learning-Based Prediction of Clinical Outcomes for Children During Emergency Department Triage. *JAMA Netw Open* 2019;2(1):e186937. doi: 10.1001/jamanetworkopen.2018.6937 [published Online First: 2019/01/16]
18. Vellido A, Ribas V, Morales C, et al. Machine learning in critical care: state-of-the-art and a sepsis case study. *Biomedical engineering online* 2018;17(1):135.
19. Christodoulou E, Ma J, Collins GS, et al. A systematic review shows no performance benefit of machine learning over logistic regression for clinical prediction models. *J Clin Epidemiol* 2019;110:12-22. doi: 10.1016/j.jclinepi.2019.02.004 [published Online First: 2019/02/15]
20. Allanson E, Tuncalp O, Gardosi J, et al. Classifying the causes of perinatal death. *Bull World Health Organ* 2016;94(2):79-79A. doi: 10.2471/BLT.15.168047 [published Online First: 2016/02/26]
21. Wojcieszek AM, Reinebrant HE, Leisher SH, et al. Characteristics of a global classification system for perinatal deaths: a Delphi consensus study. *BMC pregnancy and childbirth* 2016;16(1):223.
22. Pitt C, Tawiah T, Soremekun S, et al. Cost and cost-effectiveness of newborn home visits: findings from the Newhints cluster-randomised controlled trial in rural Ghana. *The Lancet Global Health* 2016;4(1):e45-e56.
23. World Health Organization. Newborns: reducing mortality 2020 [cited 2020 02 May]. Available from: https://www.who.int/news-room/fact-sheets/detail/newborns-reducing-mortality.
24. Khan FA, Mullany LC, Wu LF, et al. Predictors of neonatal mortality: development and validation of prognostic models using prospective data from rural Bangladesh. *BMJ Glob Health* 2020;5(1):e001983. doi: 10.1136/bmjgh-2019-001983 [published Online First: 2020/03/07]
25. Chaibva BV, Olorunju S, Nyadundu S, et al. Adverse pregnancy outcomes,'stillbirths and early neonatal deaths' in Mutare district, Zimbabwe (2014): a descriptive study. *BMC pregnancy and childbirth* 2019;19(1):86.
26. Mmbaga BT, Lie RT, Kibiki GS, et al. Transfer of newborns to neonatal care unit: a registry based study in Northern Tanzania. *BMC Pregnancy and Childbirth* 2011;11(1):68. doi: 10.1186/1471-2393-11-68
27. Mitao M, Philemon R, Obure J, et al. Risk factors and adverse perinatal outcome associated with low birth weight in Northern Tanzania: a registry-based retrospective cohort study. *Asian Pacific Journal of Reproduction* 2016;5(1):75-79. doi: 10.1016/j.apjr.2015.12.014
28. Mahande MJ, Daltveit AK, Obure J, et al. Recurrence of preterm birth and perinatal mortality in northern Tanzania: registry-based cohort study. *Trop Med Int Health* 2013;18(8):962-7. doi: 10.1111/tmi.12111 [published Online First: 2013/04/16]
29. Mahande MJ, Daltveit AK, Mmbaga BT, et al. Recurrence of perinatal death in Northern Tanzania: a registry based cohort study. *BMC pregnancy and childbirth* 2013;13(1):166.
30. Isaksen AB, Østbye T, Mmbaga BT, et al. Alcohol consumption among pregnant women in Northern Tanzania 2000–2010: a registry-based study. *BMC Pregnancy and Childbirth* 2015;15(1):205. doi: 10.1186/s12884-015-0630-0
31. Chuwa FS, Mwanamsangu AH, Brown BG, et al. Maternal and fetal risk factors for stillbirth in Northern Tanzania: A registry-based retrospective cohort study. *PloS one* 2017;12(8):e0182250.

For peer review only - http://bmjopen.bmj.com/site/about/guidelines.xhtml

32. Hamilton EF, Dyachenko A, Ciampi A, et al. Estimating risk of severe neonatal morbidity in preterm births under 32 weeks of gestation. *The Journal of Maternal-Fetal & Neonatal Medicine* 2020;33(1):73-80.
33. Mahande AM, Mahande MJ. Prevalence of parasitic infections and associations with pregnancy complications and outcomes in northern Tanzania: a registry-based cross-sectional study. *BMC Infect Dis* 2016;16:78. doi: 10.1186/s12879-016-1413-6 [published Online First: 2016/02/15]
34. Temu TB, Masenga G, Obure J, et al. Maternal and obstetric risk factors associated with preterm delivery at a referral hospital in northern-eastern Tanzania. *Asian Pacific Journal of Reproduction* 2016;5(5):365-70. doi: 10.1016/j.apjr.2016.07.009
35. Bergsjo P, Mlay J, Lie RT, et al. A medical birth registry at Kilimanjaro Christian Medical Centre. *East Afr J Public Health* 2007;4(1):1-4.
36. Mahande MJ. Recurrence of Perinatal Death, Preterm Birth and Preeclampsia in Northern Tanzania: A Registry based Study. University of Bergen, 2015.
37. World Health Organization. Maternal and perinatal health: World Health Organization; 2020 [Available from: http://www.who.int/maternal_child_adolescent/topics/maternal/maternal_perinatal/en/ accessed March 02 2020.
38. Nijkamp JW, Sebire NJ, Bouman K, et al. Perinatal death investigations: What is current practice? *Semin Fetal Neonatal Med* 2017;22(3):167-75. doi: 10.1016/j.siny.2017.02.005 [published Online First: 2017/03/23]
39. Getiye Y, Fantahun M. Factors associated with perinatal mortality among public health deliveries in Addis Ababa, Ethiopia, an unmatched case control study. *BMC Pregnancy Childbirth* 2017;17(1):245. doi: 10.1186/s12884-017-1420-7 [published Online First: 2017/07/28]
40. Allanson ER, Muller M, Pattinson RC. Causes of perinatal mortality and associated maternal complications in a South African province: challenges in predicting poor outcomes. *BMC Pregnancy Childbirth* 2015;15:37. doi: 10.1186/s12884-015-0472-9 [published Online First: 2015/04/17]
41. Vogel JP, Souza JP, Mori R, et al. Maternal complications and perinatal mortality: findings of the World Health Organization Multicountry Survey on Maternal and Newborn Health. *BJOG: An International Journal of Obstetrics & Gynaecology* 2014;121(Suppl. 1):76-88. doi: 10.1111/1471-0528.12633
42. Unterscheider J, O'Donoghue K, Daly S, et al. Fetal growth restriction and the risk of perinatal mortality–case studies from the multicentre PORTO study. *BMC pregnancy and childbirth* 2014;14(1):63.
43. Mpembeni R, Jonathan R, Mughamba J. Perinatal Mortality and Associated Factors Among Deliveries in Three Municipal Hospitals of Dar Es Salaam, Tanzania. *Journal of Pediatrics & Neonatal Care* 2014;1(4) doi: 10.15406/jpnc.2014.01.00022
44. Ouyang F, Zhang J, Betran AP, et al. Recurrence of adverse perinatal outcomes in developing countries. *Bull World Health Organ* 2013;91(5):357-67. doi: 10.2471/BLT.12.111021 [published Online First: 2013/05/17]
45. Mutsaerts MA, Groen H, Buiter-Van der Meer A, et al. Effects of paternal and maternal lifestyle factors on pregnancy complications and perinatal outcome. A population-based birth-cohort study: the GECKO Drenthe cohort. *Hum Reprod* 2014;29(4):824-34. doi: 10.1093/humrep/deu006 [published Online First: 2014/02/11]
46. WHO. Preterm birth: World Health Organization; 2020 [Available from: http://www.who.int/news-room/fact-sheets/detail/preterm-birth accessed 19 February 2020.
47. World Health Organization. WHA Global nutrition targets 2025: low birth weight policy brief. Geneva: World Health Organization, 2014.

For peer review only - http://bmjopen.bmj.com/site/about/guidelines.xhtml

48. StataCorp. Stata Statistical Software: Release 15. College Station, TX: StataCorp LLC, 2017.
49. R Core Team. R: A Language and Environment for Statistical Computing. Vienna, Austria: R Foundation for Statistical Computing, 2020.
50. Dwivedi AK. Artificial neural network model for effective cancer classification using microarray gene expression data. *Neural Computing and Applications* 2016;29(12):1545-54. doi: 10.1007/s00521-016-2701-1
51. Stephens D, Diesing M. A comparison of supervised classification methods for the prediction of substrate type using multibeam acoustic and legacy grain-size data. *PLoS One* 2014;9(4):e93950. doi: 10.1371/journal.pone.0093950 [published Online First: 2014/04/05]
52. Breiman L. Random forests. *Machine learning* 2001;45(1):5-32.
53. Hastie T, Tibshirani R, Friedman J. The Elements of Statistical Learning: Data Mining, Inference, and Prediction. Second ed: Springer 2009.
54. Bayesian networks classifiers for gene-expression data. 2011 11th International Conference on Intelligent Systems Design and Applications; 2011. IEEE.
55. Musa AB. Gene expression data classification with kernel independent component analysis. *Research Journal of Mathematical and Statistical Sciences* 2014;2320:6047.
56. Dreiseitl S, Ohno-Machado L. Logistic regression and artificial neural network classification models: a methodology review. *Journal of biomedical informatics* 2002;35(5-6):352-59.
57. Sutton CD. Classification and Regression Trees, Bagging, and Boosting. Data Mining and Data Visualization2005:303-29.
58. Johnson JM, Khoshgoftaar TM. Survey on deep learning with class imbalance. *Journal of Big Data* 2019;6(1):27.
59. Chawla NV. Data mining for imbalanced datasets: An overview. Data mining and knowledge discovery handbook: Springer 2009:875-86.
60. Vickers AJ, Elkin EB. Decision curve analysis: a novel method for evaluating prediction models. *Medical Decision Making* 2006;26(6):565-74.
61. Zhang Z, Rousson V, Lee W-C, et al. Decision curve analysis: a technical note. *Annals of translational medicine* 2018;6(15)
62. Khandwala YS, Baker VL, Shaw GM, et al. Association of paternal age with perinatal outcomes between 2007 and 2016 in the United States: population based cohort study. *BMJ* 2018;363:k4372. doi: 10.1136/bmj.k4372 [published Online First: 2018/11/02]
63. Tough SC, Faber AJ, Svenson LW, et al. Is paternal age associated with an increased risk of low birthweight, preterm delivery, and multiple birth? *Canadian journal of public health* 2003;94(2):88-92.
64. Meng Y, Groth SW. Fathers Count: The Impact of Paternal Risk Factors on Birth Outcomes. *Matern Child Health J* 2018;22(3):401-08. doi: 10.1007/s10995-017-2407-8 [published Online First: 2017/12/09]
65. Hurley EG, DeFranco EA. Influence of paternal age on perinatal outcomes. *American Journal of Obstetrics and Gynecology* 2017;217(5):566. e1-66. e6.
66. Peneza AK, Maluka SO. 'Unless you come with your partner you will be sent back home': strategies used to promote male involvement in antenatal care in Southern Tanzania. *Global health action* 2018;11(1):1449724.
67. Gibore NS, Bali TA. Community perspectives: An exploration of potential barriers to men's involvement in maternity care in a central Tanzanian community. *PloS one* 2020;15(5):e0232939.
68. Carvalho CA, da Silva AA, Victora C, et al. Changes in Infant and Neonatal Mortality and Associated Factors in Eight Cohorts from Three Brazilian Cities. *Scientific Reports* 2020;10(1):1-8.

For peer review only - http://bmjopen.bmj.com/site/about/guidelines.xhtml

69. Habimana-Kabano I, Broekhuis A, Hooimeijer P. The Effects of Interpregnancy Intervals and Previous Pregnancy Outcome on Fetal Loss in Rwanda (1996–2010). *International journal of reproductive medicine* 2015;2015
70. Flores Navarro-Perez C, Gonzalez-Jimenez E, Schmidt-RioValle J, et al. Sociodemographic Factors and Adequacy of Prenatal Care Associated Perinatal Mortality in Colombian Pregnant Women. *Nutr Hosp* 2015;32(3):1091-8. doi: 10.3305/nh.2015.32.3.9179 [published Online First: 2015/09/01]
71. Ali AA, Elgessim ME, Taha E, et al. Factors associated with perinatal mortality in Kassala, Eastern Sudan: a community-based study 2010-2011. *J Trop Pediatr* 2014;60(1):79-82. doi: 10.1093/tropej/fmt075 [published Online First: 2013/09/21]
72. Nankabirwa V, Tumwine JK, Tylleskar T, et al. Perinatal mortality in eastern Uganda: a community based prospective cohort study. *PLoS One* 2011;6(5):e19674. doi: 10.1371/journal.pone.0019674 [published Online First: 2011/05/17]
73. Hinderaker SG, Olsen BE, Bergsjø PB, et al. Perinatal mortality in northern rural Tanzania. *Journal of Health, Population and Nutrition* 2003:8-17.
74. Blagus R, Lusa L. SMOTE for high-dimensional class-imbalanced data. *BMC Bioinformatics* 2013;14(1):106. doi: 10.1186/1471-2105-14-106
75. Lusa L. Improved shrunken centroid classifiers for high-dimensional class-imbalanced data. *BMC bioinformatics* 2013;14(1):64.

For peer review only

For peer review only - http://bmjopen.bmj.com/site/about/guidelines.xhtml

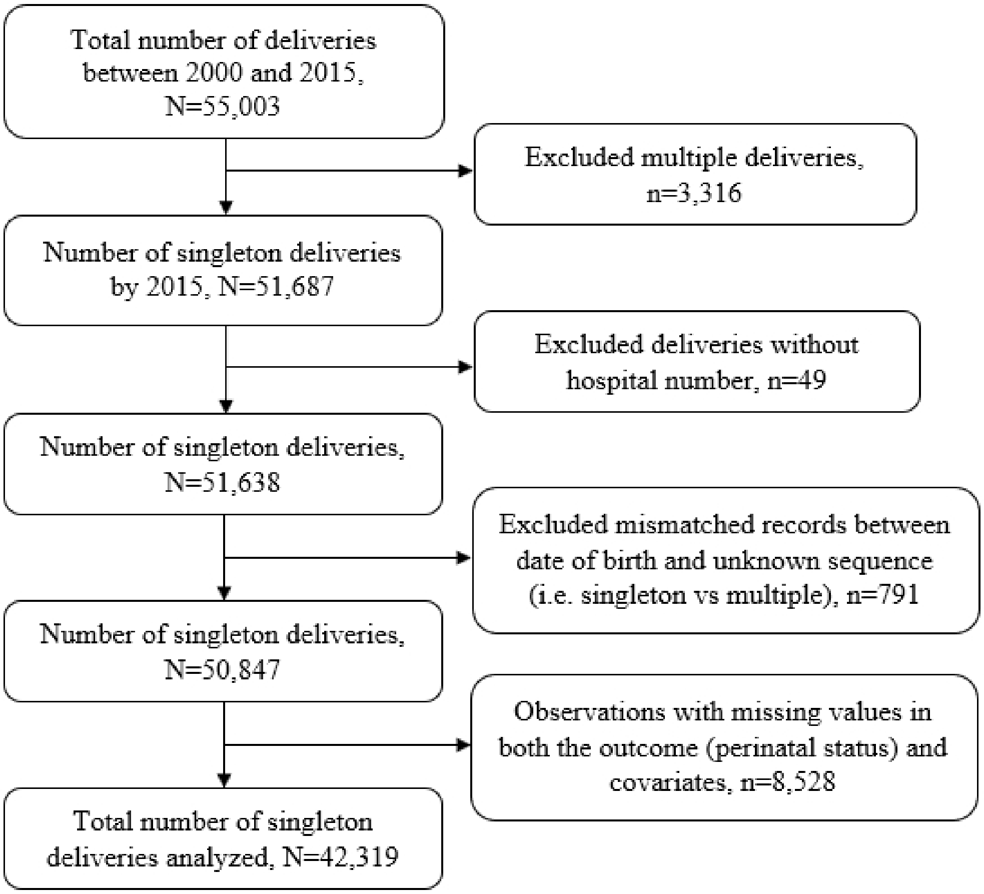

Figure 1: Schematic diagram showing the number of singleton deliveries analyzed, KCMC medical birth registry data, 2000-2015.

146x133mm (96 x 96 DPI)

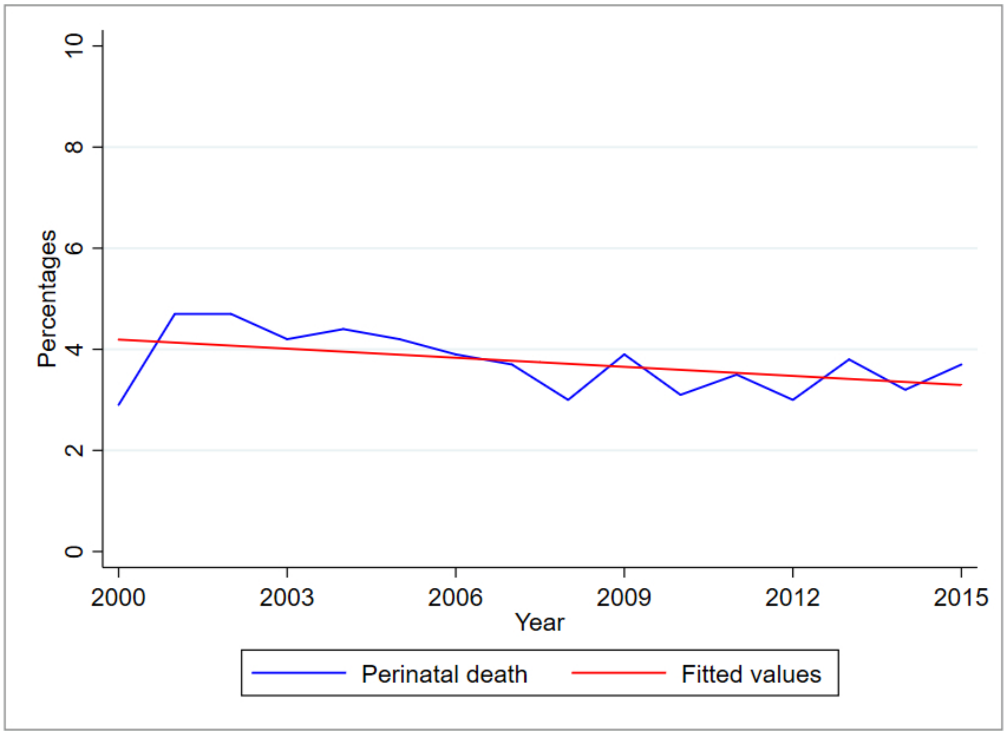

Figure 2: Trends of perinatal death, KCMC Medical Birth Registry data, 2000-2015.

298x217mm (72 x 72 DPI)

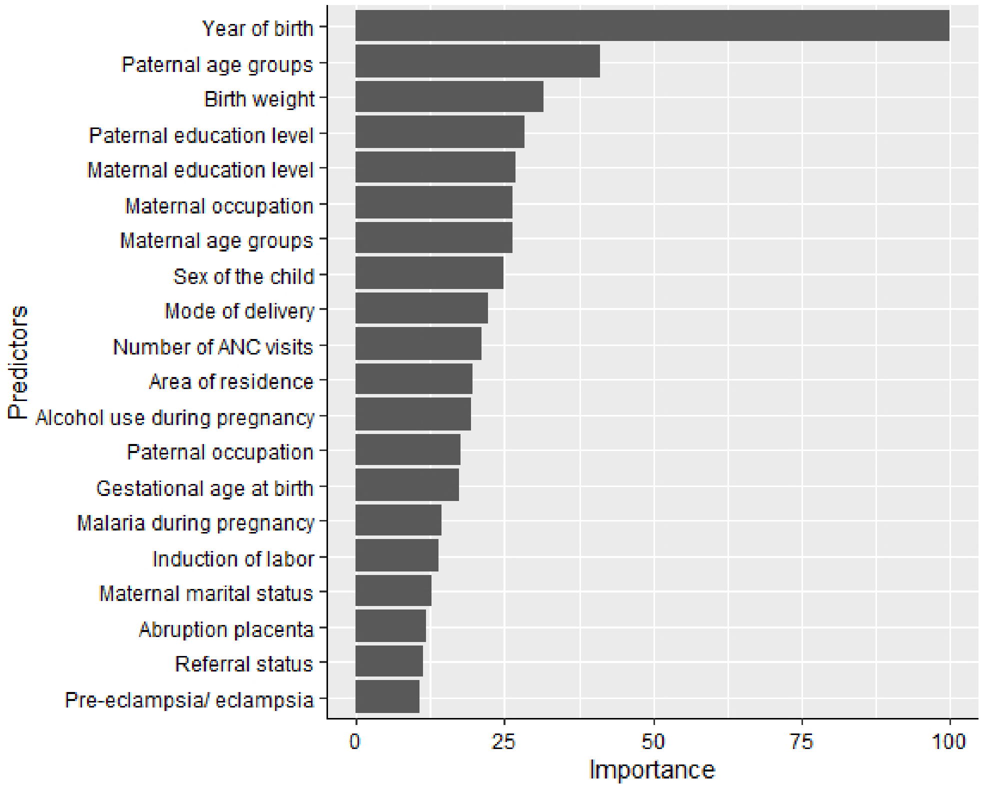

Figure 3: Variable importance of predictors for perinatal death in the random forest model scaled to have a maximum value of 100.

153x123mm (96 x 96 DPI)

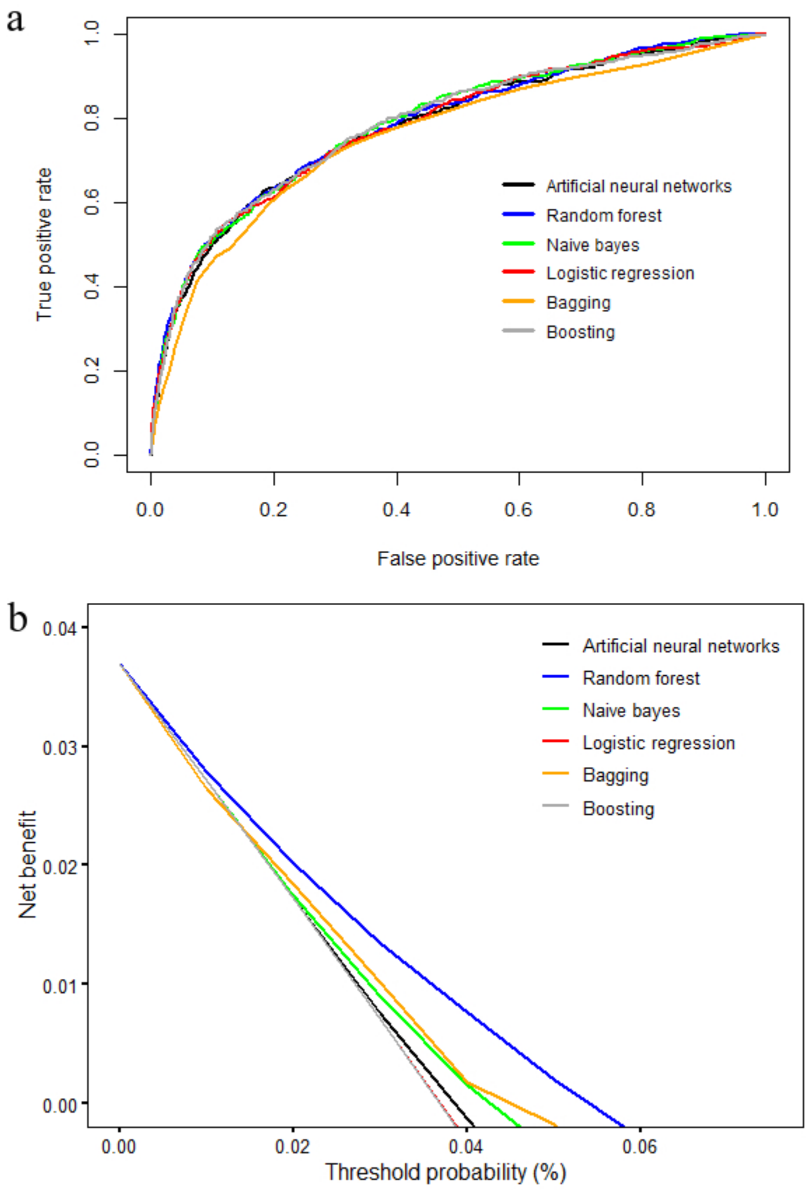

Figure 4: Prediction ability of perinatal deaths comparing different machine learning models in the test set: [a] Receiver-operating-characteristics (ROC) curves. The corresponding values of the area under the receiver-operating-characteristics curve (AUC) for each model are in Table 2. [b] Decision curve analysis. The net benefit of the machine learning models (except for boosting) is larger over a range of threshold probability values compared to that of the logistic regression model.

152x226mm (96 x 96 DPI)

```
# input Stata file
library(foreign)
data<-read.dta("D:/Documents/Innocent/PhD 2018/PhD project/Data/Results/Machine Learning Paper/complete_data_withlabels.dta")
utils::View(head(data))
summary(data)
###################################################################################
#Divide data into training and testing sets

library(caret)

set.seed(100) # For reproducabilty

trainIndex <- createDataPartition(data$perinatal_death,p=.70,list=FALSE) #divide the data based on the outcome variable in the same ratio
trainData <- data[trainIndex,]
testData  <- data[-trainIndex,]
table(data$perinatal_death)
table(trainData$perinatal_death)
table(testData$perinatal_death)
###################################################################################

#Variable/Feature Selection
###################################################################################

# install.packages("mlbench")
# install.packages("Hmisc")
# install.packages("randomForest")

#RandomForests
#install.packages("caret", "mlbench", "mlbench", "randomForest")

library(mlbench)
library(Hmisc)
library(randomForest)
library(caret)

#speed up the performance of the analysis
# library(doParallel)
# ncores <- detectCores() - 1
# cl <- makeCluster(ncores)
# registerDoParallel(cl)

#Parameter tuning and Cross validation
```

For peer review only

```
ctrl <- trainControl(method="CV",   # 10fold cross validation
                     number = 10,
                     #repeats = 10, #has no meaning for this resampling method
                     savePredictions = TRUE,
                     classProbs=TRUE,
                     allowParallel = TRUE,
                     summaryFunction = twoClassSummary
                     #search = "random" # Random searching of hyperparameters?
                     )

#Feature selection using random forests algorithm
RFMod <- train(x=trainData[,-which(names(trainData) %in% c("perinatal_death"))],
               y=trainData[,which(names(trainData) %in% c("perinatal_death"))],
               method="rf", metric="ROC", trControl=ctrl)

#---------------------------------------------------------------------------
#select and plot the Important variables from the RF model
RFImp <- varImp(RFMod, scale=T)
RFImp
plot(RFImp, main='Variable Importance')
plot(RFImp, top = 20, main='Variable Importance') #all 20 selected features

#Plot of important features with proper names
impvarsdata<-data.frame(impvars=c("Year of birth", "Paternal age groups", "Birth weight",
  "Paternal education level", "Maternal education level", "Maternal occupation",
  "Maternal age groups", "Sex of the child","Mode of delivery", "Number of ANC visits",
  "Area of residence", "Alcohol use during pregnancy", "Paternal occupation",
  "Gestational age at birth", "Malaria during pregnancy", "Induction of labor",
  "Maternal marital status", "Abruption placenta",  "Referral status",
  "Pre-eclampsia/ eclampsia"),

  rfvalues=c(100.00,      41.12,   31.51,   28.49,   26.82,  26.32,    26.18,   25.00,   22.22,
          21.13,    19.53,   19.39,   17.64,   17.29,   14.31,  13.96,    12.57,   11.96,
          11.11,    10.69))

utils::View(impvarsdata) #compare with RF below
RFImp
class(impvarsdata$rfvalues)
class(impvarsdata$impvars)

# arrange in descending order
# https://rpubs.com/Felix/7644

impvarsdata$impvars <- factor(impvarsdata$impvars,
```


For peer review only

```
                    levels = impvarsdata[order(impvarsdata$rfvalues), "impvars"])

ggplot(impvarsdata, aes(x = impvars, y = rfvalues)) + geom_bar(stat = "identity") +
  coord_flip() +  xlab("Predictors") + ylab("Importance") +
  theme(axis.text = element_text(colour = "black")) +
  theme(axis.line = element_line(colour = "black"))

ggplot(RFImp, top = 20, main='Variable Importance') #all 20 selected features
#--------------------------------------------------------------------------

# Remove the variables which were not selected from both training and testing datasets

#training
trainDataIMP <- trainData[, c("year", "fage_grp", "lbw", "feducation", "meducation2", "moccup_grp2",
"mage_grp2", "sex2", "demode_grp2", "ancvisit_grp", "residence_grp2", "alcopreg2", "PTB_LMP_grp",
"foccup_grp", "INDUCT", "MHDP10", "marstat_grp2", "CMPL04", "refdel2", "PE_E")]
trainDataIMP$perinatal_death <- trainData$perinatal_death[match(row.names(trainDataIMP),
row.names(trainData))]

#testing
testDataIMP <- testData[,c("year", "fage_grp", "lbw", "feducation", "meducation2", "moccup_grp2",
"mage_grp2", "sex2", "demode_grp2", "ancvisit_grp", "residence_grp2", "alcopreg2", "PTB_LMP_grp",
"foccup_grp", "INDUCT", "MHDP10", "marstat_grp2", "CMPL04", "refdel2",  "PE_E")]
testDataIMP$perinatal_death <- testData$perinatal_death[match(row.names(testDataIMP),
row.names(testData))]

#save both data to CSV
write.csv(x = trainDataIMP, file = "D:/Documents/Innocent/PhD 2018/PhD
project/Data/Results/Machine Learning Paper/trainDataIMP.csv", row.names = TRUE)
write.csv(x = testDataIMP, file = "D:/Documents/Innocent/PhD 2018/PhD project/Data/Results/Machine
Learning Paper/testDataIMP.csv", row.names = TRUE)

#Classification - use selected variables (from training data) to predict perinatal death (in testing data)

#Reload the saved data
TrainData <- read.csv(file = "D:/Documents/Innocent/PhD 2018/PhD project/Data/Results/Machine
Learning Paper/trainDataIMP.csv", header = TRUE, row.names = 1, stringsAsFactors = TRUE)
TestData <- read.csv(file = "D:/Documents/Innocent/PhD 2018/PhD project/Data/Results/Machine
Learning Paper/testDataIMP.csv", header = TRUE, row.names = 1, stringsAsFactors = TRUE)
##################################################################################

# 1) Logistic regression
##################################################################################
library(DMwR)
```

For peer review only

```
ctrlLReg <- trainControl(## 10-fold CV
  method = "cv",
  number = 10,
  classProbs=TRUE,
  savePredictions = TRUE,
  allowParallel = TRUE,
  sampling = "smote",
  # To correct for class imbalances (more alive than death in perinatal_death)
  # https://www.r-bloggers.com/dealing-with-unbalanced-data-in-machine-learning/
  summaryFunction = twoClassSummary
)

# Train
set.seed(1239)
lreg <- train(x=TrainData[-which(names(TrainData) %in% c("perinatal_death"))],
        y= TrainData$perinatal_death, method="glm",family=binomial(),
        trControl=ctrlLReg)

lreg
#-----------------------------------------------------------

# Test
set.seed(1239)
lreg.pred <- predict(lreg, TestData[,-which(names(TestData) %in% c("perinatal_death"))])
lreg.tab = table(pred = lreg.pred, true = TestData[,c("perinatal_death")])
lreg.Conf = confusionMatrix(lreg.pred, TestData[,c("perinatal_death")], positive =
levels(TestData[,c("perinatal_death")])[2])
lreg.Conf

##############################################################################

# 2) Naive Bayes
##############################################################################

# Load required packages
library(dplyr)      # Used by caret
#library(nnet)      # support vector machine
library(pROC)       # plot the ROC curves
#library(e1071)
# install.packages("naivebayes")
library(naivebayes)
```

```
set.seed(1236)
# Setup for cross validation
ctrlNB <- trainControl(method="CV",   # 10fold cross validation
                      number = 10,
                      savePredictions = TRUE,
                      classProbs=TRUE,
                      allowParallel = TRUE,
                      sampling = "smote",
                      summaryFunction = twoClassSummary
)

set.seed(1236)

# Train
NBModel.tune <- train(x=TrainData[-which(names(TrainData) %in% c("perinatal_death"))],
                     y= TrainData$perinatal_death,
                     method = "naive_bayes",   # Naive Bayes
                     #tuneLength = 5,                                    # 9 values of the cost function
                     #preProc = c("center","scale"), # Center and scale data
                       #not needed for this analysis
                     metric="ROC",
                     trControl=ctrlNB)

NBModel.tune
plot(NBModel.tune)

#-------------------------------------------------------------
# Test
NBModel.pred <- predict(NBModel.tune, TestData[,-which(names(TestData) %in%
c("perinatal_death"))])
NBModel.tab = table(pred = NBModel.pred, true = TestData[,c("perinatal_death")])
NBModel.Conf = confusionMatrix(NBModel.pred, TestData[,c("perinatal_death")], positive =
levels(TestData[,c("perinatal_death")])[2])
NBModel.Conf
################################################################################

# 3) Artificial nueral network (ANN)
################################################################################

# Load important packages
# library(caret)
# library(dplyr)        # Used by caret
library(nnet)       # support vector machine
# library(pROC)       # plot the ROC curves
```

For peer review only

```
set.seed(1234)
#set.seed(123)
# Setup for cross validation
ctrlANN <- trainControl(method="CV",   # 10fold cross validation
                     number = 10,
                     #savePredictions = TRUE,
                     classProbs=TRUE,
                     allowParallel = TRUE,
                     savePredictions = TRUE,
                     sampling = "smote",
                     summaryFunction = twoClassSummary
)

# Train: Artificial nueral network (ANN) model
set.seed(1234)
ANNModel.tune <- train(x=TrainData[-which(names(TrainData) %in% c("perinatal_death"))],
                    y= TrainData$perinatal_death,
                    method = "nnet",   # nueral network
                    #tuneLength = 5,                                          # 9 values of the cost function
                    #preProc = c("center","scale"),  # Center and scale data
                    #metric="ROC",
                    #linout=TRUE,
                    #maxit = 1000,
                    #tuneGrid = my.grid,
                    trace = F,
                    #tuneLength = 10,
                    #linout = 1,
                    trControl=ctrlANN,
                    metric="ROC")

ANNModel.tune
plot(ANNModel.tune)
#------------------------------------------------------------

# Test
set.seed(1234)
ANNModel.pred <- predict(ANNModel.tune, TestData[,-which(names(TestData) %in%
c("perinatal_death"))])
ANNModel.tab = table(pred = ANNModel.pred, true = TestData[,c("perinatal_death")])
ANNModel.Conf = confusionMatrix(ANNModel.pred, TestData[,c("perinatal_death")],
                          positive = levels(TestData[,c("perinatal_death")])[2])
ANNModel.Conf
################################################################################
```

For peer review only

```
# 4) Bagged Tree
##################################################################################
# Specify the training configuration
ctrlBagTree <- trainControl(method = "cv",
                            number = 10,
                            classProbs=TRUE,
                            savePredictions = TRUE,
                            allowParallel = TRUE,
                            sampling = "smote",
                            summaryFunction = twoClassSummary)  # For AUC

# Cross validate the credit model using "treebag" method;
# Track AUC (Area under the ROC curve)
set.seed(1237)  # for reproducibility
BaggedTree <- train(x=TrainData[-which(names(TrainData) %in% c("perinatal_death"))],
                    y= TrainData$perinatal_death,
                    method = "treebag",
                    metric = "ROC",
                    trControl = ctrlBagTree)
BaggedTree

#-----------------------------------------------------------
# Test
set.seed(1237)
BaggedTree.pred <- predict(BaggedTree, TestData[,-which(names(TestData) %in%
c("perinatal_death"))])
BaggedTree.tab = table(pred = BaggedTree.pred, true = TestData[,c("perinatal_death")])
BaggedTree.Conf = confusionMatrix(BaggedTree.pred, TestData[,c("perinatal_death")], positive =
levels(TestData[,c("perinatal_death")])[2])
# if you need to predict alive change to 1 at the end of the syntax
BaggedTree.Conf
##################################################################################

# 5) Boosting
##################################################################################
#install gbm package
# install.packages("gbm")
library(gbm)
# Specify the training configuration
ctrlBoosting <- trainControl(method = "cv",
                             number = 10,
                             classProbs=TRUE,
                             savePredictions = TRUE,
```

For peer review only

```
                    allowParallel = TRUE,
                    sampling = "smote",
                    summaryFunction = twoClassSummary)  # For AUC

# Cross validate the credit model using "treebag" method;
# Track AUC (Area under the ROC curve)
set.seed(1238)  # for reproducibility
Boosting <- train(x=TrainData[-which(names(TrainData) %in% c("perinatal_death"))],
              y= TrainData$perinatal_death,
              method = "gbm",
              metric = "ROC",
              trControl = ctrlBoosting)
plot(Boosting)

#-----------------------------------------------------------
# Test
set.seed(1238)
Boosting.pred <- predict(Boosting, TestData[,-which(names(TestData) %in% c("perinatal_death"))])
Boosting.tab = table(pred = Boosting.pred, true = TestData[,c("perinatal_death")])
Boosting.Conf = confusionMatrix(Boosting.pred, TestData[,c("perinatal_death")], positive =
levels(TestData[,c("perinatal_death")])[2])
Boosting.Conf
##################################################################################

# 6) Random Forests (RF)
##################################################################################
#Load additional libraries
library(caTools)
library(mlbench)

#set.seed(123)
# Setup for cross validation
ctrlRF <- trainControl(method="CV",   # 10fold cross validation
                number = 10,
                savePredictions = TRUE,
                classProbs=TRUE,
                allowParallel = TRUE,
                sampling = "smote",
                summaryFunction = twoClassSummary
                )

#Train
set.seed(1235)
RFModel.tune <- train(x=TrainData[-which(names(TrainData) %in% c("perinatal_death"))],
```

For peer review only

```
            y= TrainData$perinatal_death,
            method = "rf",   # Random Forest
            #tuneLength = 5,                              # 9 values of the cost function
            #preProc = c("center","scale"),  # Center and scale data
            metric="ROC",
            trControl=ctrlRF)

RFModel.tune
plot(RFModel.tune)

#--------------------------------------------------------------
# Test
set.seed(1235)
RFModel.pred <- predict(RFModel.tune, TestData[,-which(names(TestData) %in% c("perinatal_death"))])
RFModel.tab = table(pred = RFModel.pred, true = TestData[,c("perinatal_death")])
RFModel.Conf = confusionMatrix(RFModel.pred, TestData[,c("perinatal_death")], positive =
levels(TestData[,c("perinatal_death")])[2])
RFModel.Conf
##################################################################################

# RESULTS
##################################################################################

# Plot ROC curves
# ==============================================================================
library(ROCR)
probANN <- predict(ANNModel.tune, newdata=TestData[,-which(names(TestData) %in%
c("perinatal_death"))], type="prob")
predANN <- prediction(probANN[,2], TestData[,c("perinatal_death")])
perfANN <- performance(predANN, measure = "tpr", x.measure = "fpr")
plot(perfANN, col="Black", lwd=2)

probRF <- predict(RFModel.tune, newdata=TestData[,-which(names(TestData) %in%
c("perinatal_death"))], type="prob")
predRF <- prediction(probRF[,2], TestData[,c("perinatal_death")])
perfRF <- performance(predRF, measure = "tpr", x.measure = "fpr")
plot(perfRF, col="Blue", lwd=2, add = TRUE)

probNB <- predict(NBModel.tune, newdata=TestData[,-which(names(TestData) %in%
c("perinatal_death"))], type="prob")
predNB <- prediction(probNB[,2], TestData[,c("perinatal_death")])
perfNB <- performance(predNB, measure = "tpr", x.measure = "fpr")
plot(perfNB, col="Green", lwd=2, add = TRUE)
```

For peer review only

```
probLreg <- predict(lreg, newdata=TestData[,-which(names(TestData) %in% c("perinatal_death"))],
type="prob")
predLreg <- prediction(probLreg[,2], TestData[,c("perinatal_death")])
perfLreg <- performance(predLreg, measure = "tpr", x.measure = "fpr")
plot(perfLreg, col="Red", lwd=2, add = TRUE)

probBaggedTrees <- predict(BaggedTree, newdata=TestData[,-which(names(TestData) %in%
c("perinatal_death"))], type="prob")
predBaggedTrees <- prediction(probBaggedTrees[,2], TestData[,c("perinatal_death")])
perfBaggedTrees <- performance(predBaggedTrees, measure = "tpr", x.measure = "fpr")
plot(perfBaggedTrees, col="Orange", lwd=2, add = TRUE)

probBoosting <- predict(Boosting, newdata=TestData[,-which(names(TestData) %in%
c("perinatal_death"))], type="prob")
predBoosting <- prediction(probBoosting[,2], TestData[,c("perinatal_death")])
perfBoosting <- performance(predBoosting, measure = "tpr", x.measure = "fpr")
plot(perfBoosting, col="DarkGray", lwd=2, add = TRUE)

#all plots together
par(mar=c(5,5,1,1), cex=0.9) # cex = text font size, mar=margins (left, bottom, top, right)
plot(perfANN, col="Black", lwd=2)
plot(perfRF, col="Blue", lwd=2, add = TRUE)
plot(perfNB, col="Green", lwd=2, add = TRUE)
plot(perfLreg, col="Red", lwd=2, add = TRUE)
plot(perfBaggedTrees, col="Orange", lwd=2, add = TRUE)
plot(perfBoosting, col="DarkGray", lwd=2, add = TRUE)

legend(0.55,0.7, legend = c("Artificial neural networks", "Random forest", "Naive bayes",
                "Logistic regression", "Bagging", "Boosting"),
    col = c("Black", "Blue", "Green", "Red", "Orange", "DarkGray"),
    lty = 1,lwd = 3,bty = "n", y.intersp = 1.5, cex=0.8)
# ===========================================================================

#Compute AUC values
#===========================================================================
ANN_auc <- performance(predANN, measure = "auc")
ANN_auc <- ANN_auc@y.values[[1]]

RF_auc <- performance(predRF, measure = "auc")
RF_auc <- RF_auc@y.values[[1]]

NB_auc <- performance(predNB , measure = "auc")
NB_auc <- NB_auc@y.values[[1]]
```

For peer review only

```
Lreg_auc <- performance(predLreg , measure = "auc")
Lreg_auc <- Lreg_auc@y.values[[1]]

Bagged_auc <- performance(predBaggedTrees , measure = "auc")
Bagged_auc <- Bagged_auc@y.values[[1]]

Boosting_auc <- performance(predBoosting , measure = "auc")
Boosting_auc <- Boosting_auc@y.values[[1]]

#an alternative and short way compared to the above syntax
aucANN<-auc(as.numeric(TestData$perinatal_death), probANN[,2])#ANN
aucRF<-auc(as.numeric(TestData$perinatal_death), probRF[,2])#RF
aucNB<-auc(as.numeric(TestData$perinatal_death), probNB[,2])#NB
aucLreg<-auc(as.numeric(TestData$perinatal_death), probLreg[,2])#Lreg
aucBagging<-auc(as.numeric(TestData$perinatal_death), probBaggedTrees[,2])#Bagging
aucBoosting<-auc(as.numeric(TestData$perinatal_death), probBoosting[,2])#Boosting
#==============================================================================

#Compute CI for AUC
#==============================================================================
library(pROC)
ci.auc(as.numeric(TestData$perinatal_death), probANN[,2])#ANN
ci.auc(as.numeric(TestData$perinatal_death), probRF[,2])#RF
ci.auc(as.numeric(TestData$perinatal_death), probNB[,2])#NB
ci.auc(as.numeric(TestData$perinatal_death), probLreg[,2])#Lreg
ci.auc(as.numeric(TestData$perinatal_death), probBaggedTrees[,2])#Bagging
ci.auc(as.numeric(TestData$perinatal_death), probBoosting[,2])#Boosting
#==============================================================================

#Comparison of AUC for different machines using Delong's Test
library(ROCR)
roc.test(aucLreg, aucANN, method = "delong") #delong test is the default
roc.test(aucLreg, aucNB)
roc.test(aucLreg, aucBagging)
roc.test(aucLreg, aucBoosting)
roc.test(aucLreg, aucRF)
#==============================================================================
#Compute CT for Sens, Spec, Prevelance ... etc
#==============================================================================

library(epiR)
epi.tests(ANNModel.Conf$table, conf.level = 0.95)
epi.tests(RFModel.Conf$table, conf.level = 0.95)
epi.tests(NBModel.Conf$table, conf.level = 0.95)
```

For peer review only

```
epi.tests(lreg.Conf$table, conf.level = 0.95)
epi.tests(BaggedTree.Conf$table, conf.level = 0.95)
epi.tests(Boosting.Conf$table, conf.level = 0.95)

#================================================================================

# Summary statistics of the Results
# ==============================================================================
library(caret)
results <- resamples(list(ANN=ANNModel.tune, RF=RFModel.tune,
              NB=NBModel.tune, BaggedTree=BaggedTree, Lreg=lreg, Boosting= Boosting))
# Table comparison
summary(results)

# boxplot comparison
bwplot(results)
# Dot-plot comparison
dotplot(results)
# ==============================================================================

# Decision curve analysis
# DCA analysis
# ==============================================================================
library("tidyverse")
library("dplyr")

#Predict Perinatal death
ANNModel.pred <- predict(ANNModel.tune, TestData[,-which(names(TestData) %in%
c("perinatal_death"))], type = "prob")
RFModel.pred <- predict(RFModel.tune, TestData[,-which(names(TestData) %in% c("perinatal_death"))],
type = "prob")
NBModel.pred <- predict(NBModel.tune, TestData[,-which(names(TestData) %in%
c("perinatal_death"))], type = "prob")
lreg.pred <- predict(lreg, TestData[,-which(names(TestData) %in% c("perinatal_death"))], type = "prob")
BaggedTree.pred <- predict(BaggedTree, TestData[,-which(names(TestData) %in%
c("perinatal_death"))], type = "prob")
Boosting.pred <- predict(Boosting, TestData[,-which(names(TestData) %in% c("perinatal_death"))], type
= "prob")

dcadata <- TestData %>%select(c(perinatal_death))
source("D:/Documents/Innocent/PhD 2018/PhD project/Data/Results/Machine Learning Paper/dca.r")
  # This file is avaible at http://www.decisioncurveanalysis.org
library(reshape2)
dcadata$ANN<-as.numeric(ANNModel.pred$Died)
```

```
dcadata$RF<-as.numeric(RFModel.pred$Died)
dcadata$NB<-as.numeric(NBModel.pred$Died)
dcadata$Lreg<-as.numeric(lreg.pred$Died)
dcadata$Bagging<-as.numeric(BaggedTree.pred$Died)
dcadata$Boosting <- as.numeric(Boosting.pred$Died)
data.set <- dcadata

attach(data.set)
data.set$perinatal_death<-as.numeric(data.set$perinatal_death)
data.set$perinatal_death<-data.set$perinatal_death-1 #0=alive, 1=died (in the testing data)

#Plot the DCA curves
library(DCA)
dca(data=data.set, outcome="perinatal_death",
   predictors=c("ANN", "RF", "NB", "Lreg", "Bagging", "Boosting"), xstart=0, ymin=0)

dcaoutput <- dca(data=data.set, outcome="perinatal_death",
             predictors=c("ANN", "RF", "NB", "Lreg", "Bagging", "Boosting"), xstart=0,xstop=0.3, ymin=0)

dcadf <- data.frame(dcaoutput$net.benefit)
temp <- melt(dcadf, id="threshold",
           measure=c("ANN", "RF", "NB", "Lreg", "Bagging", "Boosting"))

library(ggplot2)
ggplot(temp,
      aes(x=threshold,
         y=value,
         colour=variable,
         group=variable)) + geom_line() +
 geom_line(size=0.8) + # Thicker line
 coord_cartesian(xlim = c(0, 0.075), ylim= c(0, 0.04)) +
 labs(x="Threshold probability (%)") + labs(y="Net benefit") +
 theme_minimal()  + theme(legend.position = c(0.8, 0.8),
                        text = element_text(size=12), #text size
                        panel.grid.major = element_blank(), #grid lines
                        panel.grid.minor = element_blank(),
                        axis.line = element_line(colour = "black"), # solid line
                        axis.ticks = element_line(size = 1), #tick marks
                        panel.border = element_rect(linetype = "solid",
                                                  fill = NA, colour = "black"), #axis boarders
                        axis.text = element_text(color = "black")) + #text color
 scale_color_manual(values = c("Black", "Blue", "Green", "Red", "Orange",
                             "DarkGray"), #Use same colors as with ROC curve
                  labels = c("Artificial neural networks",
```

```
                "Random forest",
                "Naive bayes",
                "Logistic regression",
                "Bagging",
                "Boosting")) + labs(color="")
# ==========================================================================

#Combining the a) ROC and b) DC plots on top of one another.

# install.packages("magick")
library(magick)

Figure3_roc<-image_read("D:/Documents/Innocent/PhD 2018/PhD project/Data/Results/Machine Learning Paper/Figure3_roc.tiff")
Figure3_roc<-image_annotate(Figure3_roc, "a", font = 'Times', size = 30)
Figure3_roc
Figure3_deccurve2<-image_read("D:/Documents/Innocent/PhD 2018/PhD project/Data/Results/Machine Learning Paper/Figure3_deccurve.tiff")
Figure3_deccurve2<-image_annotate(Figure3_deccurve2, "b", font = 'Times', size = 30)
Figure3_deccurve2

img <- c(Figure3_roc, Figure3_deccurve2) #combine the two images
# img <- image_scale(img) #size of the images
img
image_info(img)

img2<-image_append(image_scale(img), stack = TRUE) %>%
  image_border("grey", "1x1")
img2
image_info(img2)

#export the image to tiff format (better quality)
image_write(img2, path = "D:/Documents/Innocent/PhD 2018/PhD project/Data/Results/Machine Learning Paper/Figure3.tiff",
      format = "tiff")
# ==========================================================================
```

STROBE 2007 (v4) Statement—Checklist of items that should be included in reports of *cohort studies*

Section/Topic	Item #	Recommendation	Reported on page #
**Title and abstract**	1	(*a*) Indicate the study's design with a commonly used term in the title or the abstract	1
		(*b*) Provide in the abstract an informative and balanced summary of what was done and what was found	2
**Introduction**			
Background/rationale	2	Explain the scientific background and rationale for the investigation being reported	3-5
Objectives	3	State specific objectives, including any prespecified hypotheses	1, 4
**Methods**			
Study design	4	Present key elements of study design early in the paper	5
Setting	5	Describe the setting, locations, and relevant dates, including periods of recruitment, exposure, follow-up, and data collection	5
Participants	6	(*a*) Give the eligibility criteria, and the sources and methods of selection of participants. Describe methods of follow-up	5
		(*b*) For matched studies, give matching criteria and number of exposed and unexposed	NA
Variables	7	Clearly define all outcomes, exposures, predictors, potential confounders, and effect modifiers. Give diagnostic criteria, if applicable	6-7
Data sources/ measurement	8*	For each variable of interest, give sources of data and details of methods of assessment (measurement). Describe comparability of assessment methods if there is more than one group	5-6
Bias	9	Describe any efforts to address potential sources of bias	7
Study size	10	Explain how the study size was arrived at	9
Quantitative variables	11	Explain how quantitative variables were handled in the analyses. If applicable, describe which groupings were chosen and why	6-7
Statistical methods	12	(*a*) Describe all statistical methods, including those used to control for confounding	7-9
		(*b*) Describe any methods used to examine subgroups and interactions	NA
		(*c*) Explain how missing data were addressed	NA
		(*d*) If applicable, explain how loss to follow-up was addressed	NA
		(*e*) Describe any sensitivity analyses	9
**Results**			

For peer review only

Participants	13*	(a) Report numbers of individuals at each stage of study—eg numbers potentially eligible, examined for eligibility, confirmed eligible, included in the study, completing follow-up, and analysed	9
		(b) Give reasons for non-participation at each stage	NA
		(c) Consider use of a flow diagram	9
Descriptive data	14*	(a) Give characteristics of study participants (eg demographic, clinical, social) and information on exposures and potential confounders	9-10
		(b) Indicate number of participants with missing data for each variable of interest	9
		(c) Summarise follow-up time (eg, average and total amount)	9
Outcome data	15*	Report numbers of outcome events or summary measures over time	10
Main results	16	(*a*) Give unadjusted estimates and, if applicable, confounder-adjusted estimates and their precision (eg, 95% confidence interval). Make clear which confounders were adjusted for and why they were included	NA
		(*b*) Report category boundaries when continuous variables were categorized	10-12
		(*c*) If relevant, consider translating estimates of relative risk into absolute risk for a meaningful time period	NA
Other analyses	17	Report other analyses done—eg analyses of subgroups and interactions, and sensitivity analyses	13-15
**Discussion**			
Key results	18	Summarise key results with reference to study objectives	16
**Limitations**			
Interpretation	20	Give a cautious overall interpretation of results considering objectives, limitations, multiplicity of analyses, results from similar studies, and other relevant evidence	16-18
Generalisability	21	Discuss the generalisability (external validity) of the study results	18
**Other information**			
Funding	22	Give the source of funding and the role of the funders for the present study and, if applicable, for the original study on which the present article is based	19

*Give information separately for cases and controls in case-control studies and, if applicable, for exposed and unexposed groups in cohort and cross-sectional studies.

Note: An Explanation and Elaboration article discusses each checklist item and gives methodological background and published examples of transparent reporting. The STROBE checklist is best used in conjunction with this article (freely available on the Web sites of PLoS Medicine at http://www.plosmedicine.org/, Annals of Internal Medicine at http://www.annals.org/, and Epidemiology at http://www.epidem.com/). Information on the STROBE Initiative is available at www.strobe-statement.org.

For peer review only
